# Association of genetically predicted 486 blood metabolites on the risk of Alzheimer’s disease: a Mendelian randomization study

**DOI:** 10.3389/fnagi.2024.1372605

**Published:** 2024-04-12

**Authors:** Qiqi Yang, Xinyu Han, Min Ye, Tianxin Jiang, Baoguo Wang, Zhenfeng Zhang, Fei Li

**Affiliations:** ^1^Second Affiliated Hospital of Anhui University of Chinese Medicine, Hefei, China; ^2^The First Clinical Medical School, Anhui University of Chinese Medicine, Hefei, China; ^3^Intelligent Manufacturing Institute, Hefei University of Technology, Hefei, China

**Keywords:** metabolites, Alzheimer’s disease, Mendelian randomization, causality, genome-wide association study, epiandrosterone sulfate

## Abstract

**Background:**

Studies have reported that metabolic disturbance exhibits in patients with Alzheimer’s disease (AD). Still, the presence of definitive evidence concerning the genetic effect of metabolites on AD risk remains insufficient. A systematic exploration of the genetic association between blood metabolites and AD would contribute to the identification of new targets for AD screening and prevention.

**Methods:**

We conducted an exploratory two-sample Mendelian randomization (MR) study aiming to preliminarily identify the potential metabolites involved in AD development. A genome-wide association study (GWAS) involving 7,824 participants provided information on 486 human blood metabolites. Outcome information was obtained from a large-scale GWAS meta-analysis of AD, encompassing 21,982 cases and 41,944 controls of Europeans. The primary two-sample MR analysis utilized the inverse variance weighted (IVW) model while supplementary analyses used Weighted median (WM), MR Egger, Simple mode, and Weighted mode, followed by sensitivity analyses such as the heterogeneity test, horizontal pleiotropy test, and leave-one-out analysis. For the further identification of metabolites, replication and meta-analysis with FinnGen data, steiger test, linkage disequilibrium score regression, confounding analysis, and were conducted for further evaluation. Multivariable MR was performed to assess the direct effect of metabolites on AD. Besides, an extra replication analysis with EADB data was conducted for final evaluation of the most promising findings.

**Results:**

After rigorous genetic variant selection, IVW, complementary analysis, sensitivity analysis, replication and meta-analysis with the FinnGen data, five metabolites (epiandrosterone sulfate, X-12680, pyruvate, docosapentaenoate, and 1-stearoylglycerophosphocholine) were identified as being genetically associated with AD. MVMR analysis disclosed that genetically predicted these four known metabolites can directly influence AD independently of other metabolites. Only epiandrosterone sulfate and X-12680 remained suggestive significant associations with AD after replication analysis with the EADB data.

**Conclusion:**

By integrating genomics with metabonomics, this study furnishes evidence substantiating the genetic association of epiandrosterone sulfate and X-12680 with AD. These findings hold significance for the screening, prevention, and treatment strategies for AD.

## 1 Introduction

Alzheimer’s disease (AD) is the most common kind of dementia, affecting approximately 50 million individuals worldwide (GBD 2019 Dementia Forecasting Collaborators, 2022). Predictions indicate a rise to over 152 million by 2050 owing to the aging population. The financial impact of dementia is also escalating, with expected global costs nearing $9.12 trillion (Lord et al., 2021). Clearly, AD is rapidly becoming a significant financial and health burden, making it one of the most critical diseases of our century (Garcia-Argibay et al., 2023).

Disappointingly, AD remains incurable (Serrano-Pozo et al., 2017). From 2000 to 2012, over 400 AD clinical trials yielded a 99.6% failure rate, starkly contrasted by a roughly 80% failure rate in anti-cancer drugs (Cummings et al., 2014). One plausible explanation for such dismal outcomes is that recognized risk factors and medication targets may be consequences rather than root causes of AD. Another factor could be that clinical trials often focus on advanced-stage patients in trials. Considering that the effectiveness of treatments for AD may stem from early intervention, there is an imperative requirement for precisely identifying early-stage individuals only with underlying pathology (Varesi et al., 2022). Although positron emission tomography (PET) and cerebrospinal fluid (CSF) biomarkers are highly accurate and sensitive for AD diagnosis, their invasiveness and substantial costs hinder population-wide screenings on a large scale (Hampel et al., 2018). In this context, blood-based biomarkers (e.g., blood metabolites), being minimally invasive, offer promising avenues for early diagnosis and monitoring (Hampel et al., 2014; Blennow and Zetterberg, 2018). More importantly, its potential modifiability might lead to effective prevention or treatment strategies (Lord et al., 2021).

The National Institutes of Health Medical Library (NIH) currently reports 817 studies on AD in relation to blood metabolism. These studies underscored the potential role of blood metabolites in AD progression. A study involving 30 AD sufferers and 40 controls identified 11 metabolites applicable for the assessment and diagnosis of AD. Of these, 1,4-butanediamine and L-ornithine showed higher diagnostic potential (Sun et al., 2020). Additionally, recent evidence by Toledo et al. indicated a significant association between 26 metabolites, including sphingolipids, and biomarkers related to AD pathology, as well as memory scores in the brain and blood of preclinical AD (Varma et al., 2018). These findings suggest that specific metabolites possess the capacity to augment the precision of clinical diagnoses pertaining to AD, and facilitate the advancement of therapeutic interventions. However, the interpretation of blood biomarkers in AD pathogenesis is complex due to potential reverse causation, or other unmeasured potential confounders like high body fat mass or socioeconomic status, which might skew the association. Undoubtedly, this complexity highlights the need for cautious interpretation of these associations in understanding AD’s etiology.

Mendelian randomization (MR) is an emerging analytical technique commonly employed to deduce potential links between various exposures and their outcomes (Smith and Ebrahim, 2003). The utilization of the MR approach serves as a crucial alternative strategy in situations where randomized controlled trials (RCTs) are lacking, as it offers dependable evidence regarding the correlation between exposures and disease risks (Zuccolo and Holmes, 2017). To be specific, Single nucleotide polymorphisms (SNPs) are used in MR design as the unconfounded instrumental variables (IVs) to serve as proxies for the phenotypes of interest. This approach mimics RCTs due to the genetic variation’s random allocation during fertilization, thereby minimizing confounding bias in establishing causal relationships (Burgess et al., 2013). Moreover, the formation of genotypes occurs prior to approximate the desired phenotypes. The diseases and remains stable regardless of disease progression, directly avoiding the possibility of reverse causation in the analysis. While some MR studies have explored the associations between single or common exposure factors (e.g., such as educational attainment (Stern et al., 1994), smoking (Barnes and Yaffe, 2011), body mass index (Buchman et al., 2005; Kivipelto et al., 2005)) and AD, no large-scale study thoroughly examine the effect of global blood metabolites on AD to date.

Here, we leveraged genome-wide association study (GWAS) data to perform an extensive MR analysis of 486 blood metabolites. Our research attempts to shed light on the metabolism-related etiology of AD and might help make strategies for AD screening, prevention, and therapy.

## 2 Materials and methods

### 2.1 Study design

**Figure 1:**
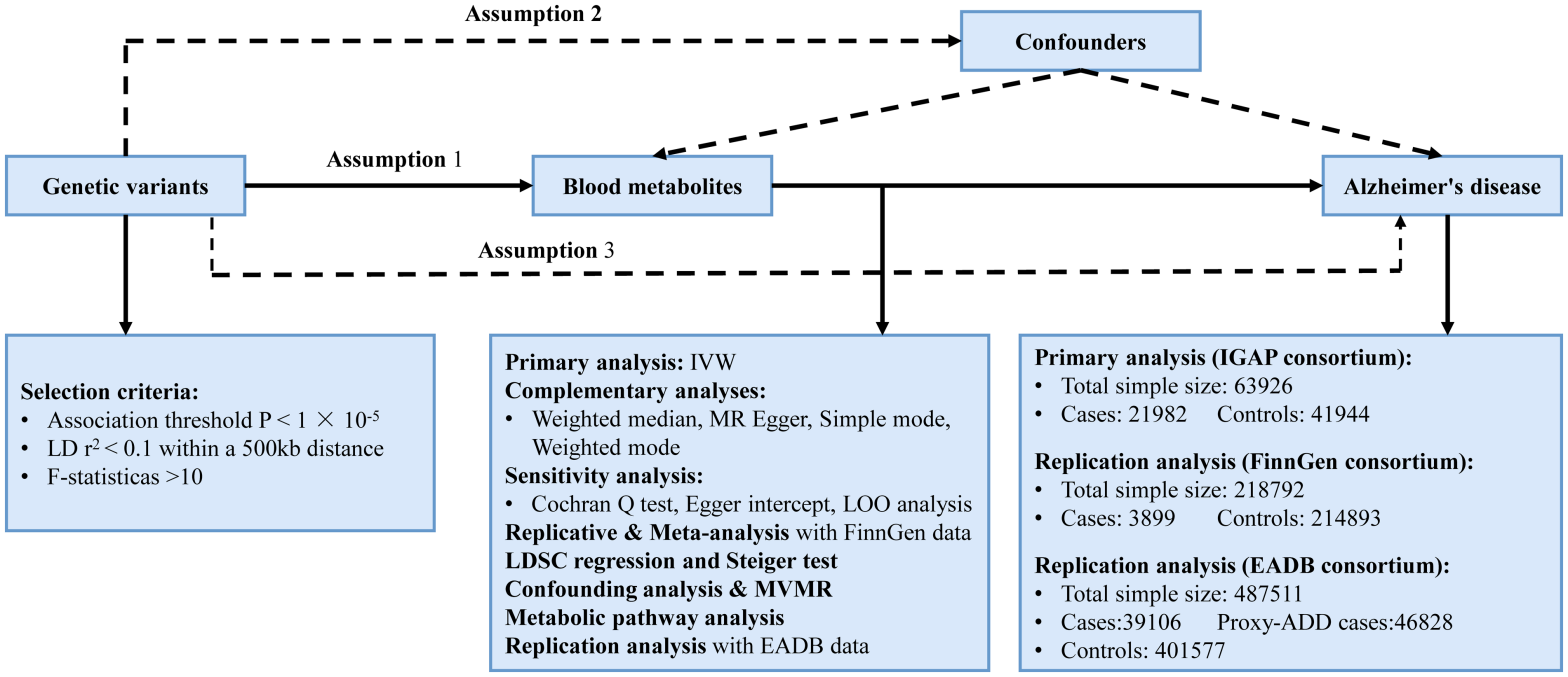
The overview of this MR study. Assumption 1. IVs exhibit strong correlation with the metabolites; Assumption 2. IVs must be free from confounding factors influencing AD; Assumption 3. The impact of IVs on AD is mediated exclusively through these metabolites. IVW, inverse variance weighted; LD, linkage disequilibrium; LDSC, linkage disequilibrium score; LOO analysis, leave-one-out analysis; MVMR, multivariable Mendelian randomization analysis; SNPs, single nucleotide polymorphisms; WM, weighted median; IGAP, the International Genomics of Alzheimer’s Project; EADB, the European Alzheimer & Dementia Biobank; proxy-ADD, proxy AD and related dementia.

Using a two-sample MR design, we carefully assessed the genetic links between 486 human blood metabolites and AD risk in this investigation. Adhering to MR’s three foundational hypotheses, we ensured that: (1) IVs exhibit strong correlation with the metabolites; (2) IVs must be free from confounding factors influencing AD; (3) The impact of IVs on AD is mediated exclusively through these metabolites (Boef et al., 2015). The design is derived from the research conducted by Cai et al. (2022). The overview of our study is presented in Figure 1.

All statistical analyses in this study were performed in R software (Version 4.2.1) by the “TwoSampleMR” package (Version 0.5.4), the “ieugwasr” package (Version 0.1.5), the “meta” package (Version 6.5.9), the “forestplot” package (Version 1.1.1), the “ggplot2” package (Version 3.4.3), the “ldscr” package (Version 0.1.0), the “MendelianRandomization” package (Version 0.9.0), and the “MVMR” package (Version 0.4.0).

### 2.2 GWAS data for blood metabolites

We utilized the GWAS dataset from the Metabolomics GWAS Server, which is the most comprehensive dataset for blood metabolites originating from Shin et al.’s (2014) study of 7,824 Europeans. The dataset comprises 2.1 million SNPs linked to 486 metabolites, of which 309 are known and 177 are yet to be classified (Shin et al., 2014). These known metabolites are categorized into 8 distinct classes according to the Kyoto Encyclopedia of Genes and Genomes (KEGG) database, including cofactors and vitamins, energy, amino acid, carbohydrate, lipid, nucleotide, peptide, and xenobiotic metabolism (Supplementary Table S1).

### 2.3 GWAS data for AD

Our primary GWAS data for AD was sourced from the International Genomics of Alzheimer’s Project (IGAP). This comprehensive two-stage study focuses on individuals of European descent (Kunkle et al., 2019). We utilized data from stage 1, totaling 63,926 participants (21,982 AD cases and 41,944 controls) that were available via the Integrative Epidemiology Unit (IEU) GWAS database, which is publicly available at the website: https://gwas.mrcieu.ac.uk. AD diagnoses were confirmed via autopsy or clinical records.

For replication and meta-analysis, we employed data from the FinnGen consortium’s G6 dataset^1^ with the phenocode G6_ALZHEIMER, including 218,792 European individuals with 3,899 AD cases and 214,893 controls. For FinnGen AD data, the diagnosis of AD was based on the International Classification of Diseases (ICD) diagnosis codes (version 10) or ICD (version 9). In addition, we also replicated our findings using AD data from the European Alzheimer & Dementia Biobank (EADB) stage I (Bellenguez et al., 2022), the largest AD genomic consortia. The data was based on 39,106 clinically diagnosed AD cases, 46,828 proxy AD and related dementia (proxy-ADD) cases, 401,577 controls, which can be obtained from GWAS Catalog^2^ with accession number GCST90027158. Proxy-ADD was only identified from the UK Biobank via questionnaire data asking if parents of the participants had AD.

### 2.4 Instruments selection

A sequential procedure was executed to screen IVs pertaining to blood metabolites. First, we chose a more lenient criterion of *p* < 1 × 10^−5^ [pairwise linkage disequilibrium (LD) r^2^ < 0.1 within a 500 kb distance], which was extensively utilized in prior studies, considering the small number of SNPs that achieved genome-wide significance (Choi et al., 2019; Sanna et al., 2019; Yang et al., 2020). Second, to minimize bias resulting from weak instruments, we assessed the statistical strength of each SNP using F statistics. SNPs with *F* < 10 were deemed weak instruments and were thus excluded, ensuring the remaining SNPs provided adequate variance for their respective metabolites. Exposure SNPs were then extracted from the outcome data, discarding any SNPs connected to the outcome (*p* < 1 × 10^−5^). For SNPs not present in the outcome, we identified high LD proxies (*r*^2^ > 0.8) using the European reference panel of the 1,000 Genomes Project, excluding SNPs without suitable proxies. Afterwards, we conducted harmonization to align the alleles of exposure-and outcome-SNPs, removing palindromic SNPs with high effect allele frequencies (EAF > 0.42) or conflicting alleles (e.g., A/G vs. A/C). Finally, our MR analysis only included metabolites represented by over 2 SNPs (Gill et al., 2019).

### 2.5 Primary analysis and sensitivity analysis

Assuming the validity of all SNPs, the random-effect inverse variance weighted (IVW) offers the most accurate assessment. The IVW methods were utilized as the primary analysis to evaluate the genetic link between blood metabolites and AD using *p* < 0.05 as the screening condition. Developed by Burgess et al. (2013), IVW method is particularly advantageous for its robust detection capability, with widespread application in MR studies. Though a Bonferroni correction used for multiple testing could effectively control false positives (*p* < 0.05/485 included metabolites (1.03 × 10^−4^)), it might also miss out potential metabolites involved in AD development. Considering the exploratory nature of this MR work, a value of *p* < 0.05 was used for as the threshold to determine the suggestive significant association. We believe this approach enables us to identify potential metabolites for further investigation without overly stringent criteria that might miss out on important findings.

For those estimates identified as suggestive significant (IVW *p* < 0.05) in the primary analysis, we performed sensitivity analyses to evaluate any potential biases against the MR assumptions. IVW assumes all the IVs are valid, and is prone to bias though it is the most sufficient method. Four other MR methods, including Weighted median (WM), MR Egger, Simple mode, Weighted mode, were utilized as complementary approaches. WM assumes that less than 1/2 of the IVs are invalid, whereas the weighted mode assumes that more than 1/2 of the IVs are invalid (Bowden et al., 2016). MR-Egger regression provides consistent estimates accounting for pleiotropy when all the instruments are invalid. A metabolite was considered a candidate metabolite if the estimates across these five MR models were consistent in direction and magnitude. Meanwhile, we conducted the Cochran Q test to detect any heterogeneity, acknowledging heterogeneity if the Cochran-Q test resulted in *p* < 0.05 and I^2^ > 25% (Greco et al., 2015). The assessment for horizontal pleiotropy was conducted using Egger intercepts (Bowden et al., 2015). To identify any influential data points affecting the combined IVW estimates, the analysis of Leave-One-Out (LOO) was implemented.

Hence, metabolites potentially implicated in the development of AD were determined using the following criteria: (1) IVW derived *p* < 0.05; (2) consistent directions and magnitudes across all five MR models; (3) no heterogeneity or pleiotropy; (4) no high-influence points identified in the LOO analysis.

### 2.6 Replication and meta-analysis with the FinnGen data

To ascertain the reliability of candidate metabolites, we conducted a duplicate IVW analysis by utilizing a distinct AD GWAS dataset that we acquired via the aforementioned FinnGen consortium. After replicating the study, we proceeded with a meta-analysis to consolidate and verify the definitive set of candidate metabolites.

### 2.7 Genetic correlation and direction validation

However, the estimates obtained through MR may exhibit discrepancies from the true effects in cases where a genetic association exists between exposure and outcome (O’Connor and Price, 2018). While we excluded AD-related SNPs in selecting instrumental variables (IVs), SNPs unrelated to AD might still influence the genetics of AD. To address potential effect violations due to genetic correlations, we used Linkage Disequilibrium Score (LDSC) regression. Coinheritance is evaluated in LDSC through the examination of Chi-squared statistics derived from SNPs pertaining to two traits.

Furthermore, the Steiger test was conducted to address potential bias resulting from reverse associations (Hemani et al., 2017). The accuracy of estimations may be compromised if the genetic risk of AD is primarily influenced by a combination of SNPs rather than blood metabolites.

### 2.8 Confounding analysis and multivariable MR analysis

Even after performing sensitivity studies to find any SNPs that go against the MR assumptions, residual confounding SNPs could still exist. To address this, we carefully scrutinized the IVs for metabolites using the Phenoscanner V2 website,^3^ to determine whether each SNP was linked to established risk factors of AD. According to the study conducted by Luo et al. (2023), the following risk factors for AD were considered, including educational attainment (Stern et al., 1994), HDL cholesterol (Reitz et al., 2010), smoking (Barnes and Yaffe, 2011), body mass index (Buchman et al., 2005), diastolic blood pressure (Qiu et al., 2003), and systolic blood pressure (Kivipelto et al., 2005). If SNPs exhibited a relationship with the potential confounders at a significance level of *p* < 1 × 10^−5^, we conducted an additional IVW analysis after excluding these SNPs to affirm the stability and accuracy of our results.

To ensure adherence to MR’s second and third assumptions, we ensured genetic variants were linked to only one risk factor. However, it’s common for some genetic variants to be relate to multiple risk factors, a phenomenon known as pleiotropy (White et al., 2016). In such instances, the utilization of multivariable Mendelian randomization (MVMR) can effectively account for interactions between genetic variations, thereby enabling the distinction between the direct impact of each exposure and the overall effect assessed through univariable MR. The MVMR approach was employed to account for the interactions of the identified metabolites (Bowden et al., 2015). Specifically, we combined the IVs of all the identified metabolites and then conducted LD clumping (LD *r*^2^ < 0.1 within a 500 kb distance) to obtain a novel set of independent IVs, which was then extracted from the AD dataset. Harmonization was conducted to align the effect alleles. The IVW method of multivariate MR is to regress all exposed SNPs with the outcome, weighting for the inverse variance of the outcome.

### 2.9 Metabolic pathway analysis

To figure out biological mechanism underlying identified metabolites, we carried out metabolic pathway analyses utilizing MetaboAnalyst 5.0, an online tool known for its user-friendly interface and streamlined approach to metabolomics data analysis. The tool can be accessed at https://www.metaboanalyst.ca/.

### 2.10 Replication analysis with the EADB data

To ascertain the reliability of identified metabolites, we made a duplicate IVW analysis using AD data from the EADB stage I, the largest AD genomic consortia. The metabolites which were consistent across all three MR-studies (IGAP-MR, FinnGen-MR and EADB-MR) were considered to be the most promising metabolites with genetic associations.

## 3 Results

After completing the instrument selection process, 485 metabolites were retained for MR estimation, excluding one due to having only two SNPs. The SNP count for each metabolite varies from 3 to 407, with all SNPs exhibiting F statistics exceeding 10, demonstrating the absence of weak instruments. Detailed information on IVs is available in Supplementary Table S2.

### 3.1 Primary analysis and sensitivity analysis

**Figure 2:**
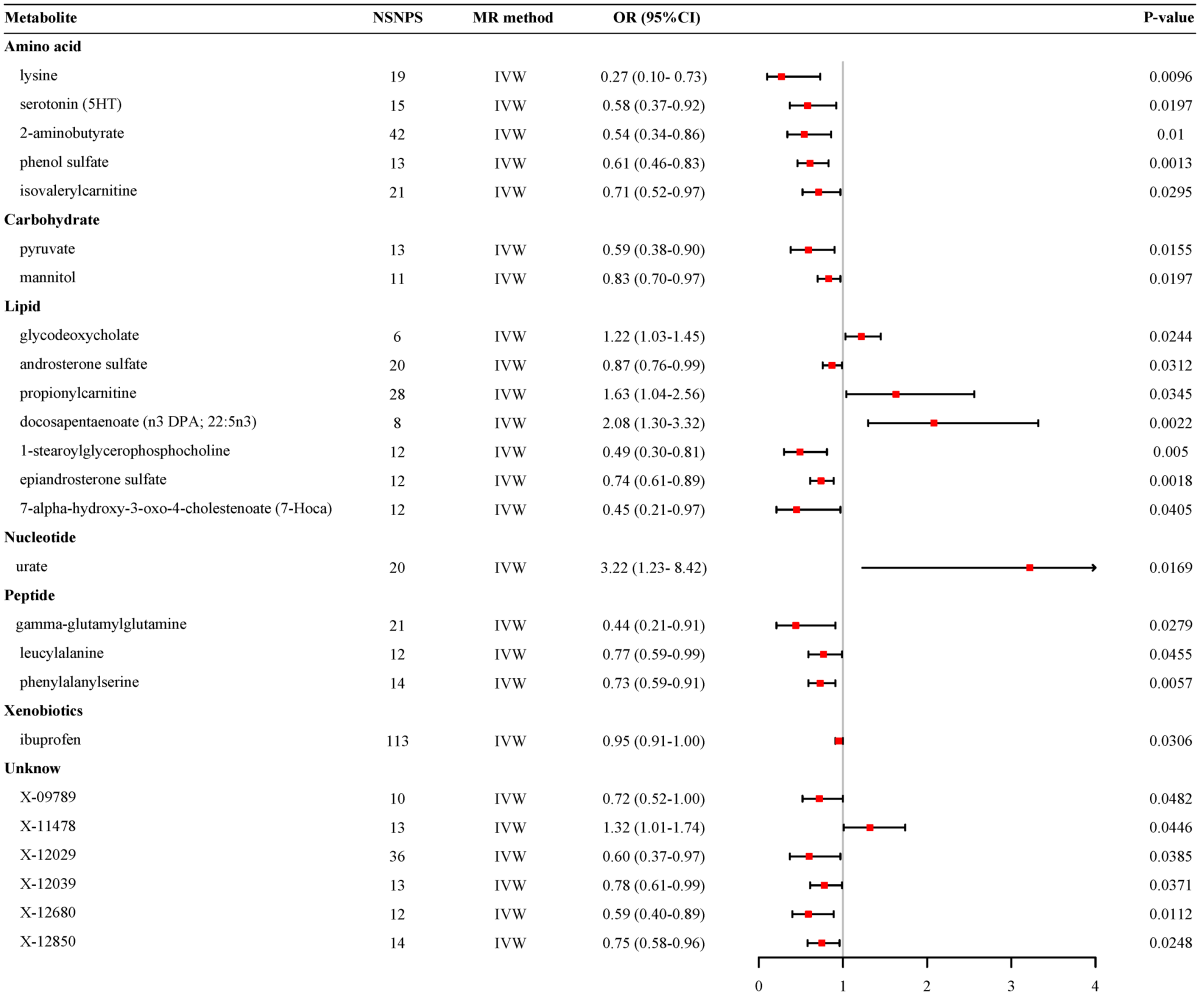
Forest plot for the genetic association of metabolites on the risk of Alzheimer’s disease derived from IVW. IVW, inverse variance weighted; OR, odds ratio; 95% CI, confidence interval; NSNPS, number of single nucleotide polymorphisms.

**Figure 3:**
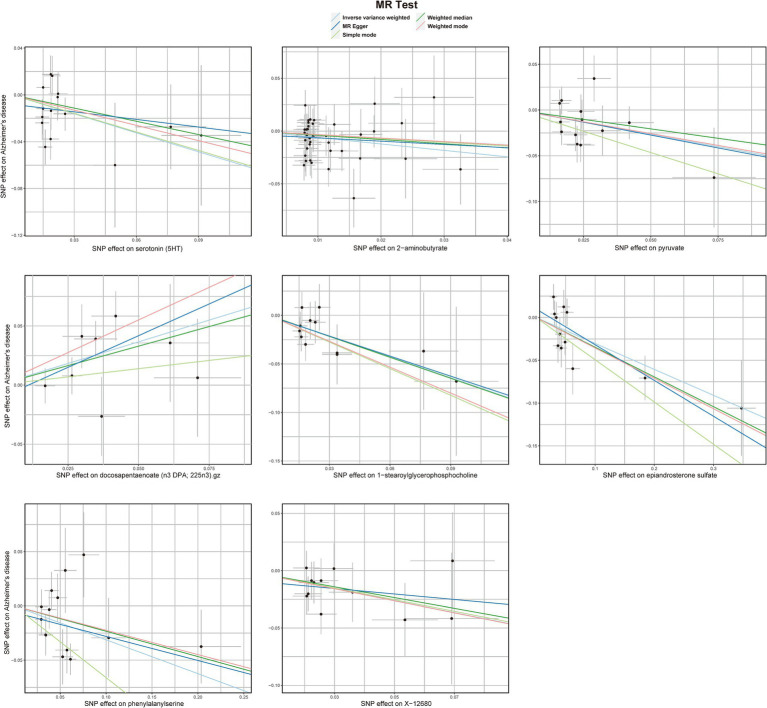
Scatterplot for the suggestive significant MR association (*p* < 0.05) between metabolites and Alzheimer’s disease. SNP, single nucleotide polymorphism.

The preliminary IVW analysis conducted in our study revealed a suggestively significant correlation between 25 metabolites and AD (Figure 2). Among these, 19 were identifiable and categorized into various groups such as amino acids, carbohydrates, lipids, nucleotides, peptides, and xenobiotics, while 6 remained chemically unknown. Further complementary analyses and sensitivity assessments narrowed down these metabolites to 8 candidates with potential involvement in AD development. These included serotonin (5HT) (OR = 0.58, 95%CI: 0.37–0.92, *p* = 0.0197), 2-aminobutyrate (OR = 0.54, 95% CI: 0.34–0.86, *p* = 0.0099), pyruvate (OR = 0.59, 95% CI: 0.38–0.90, *p* = 0.0155), docosapentaenoate (n3 DPA; 22:5n3) (OR = 2.08, 95% CI: 1.30–3.32, *p* = 0.0022), 1-stearoylglycerophosphocholine (OR = 0.49, 95% CI: 0.30–0.81, *p* = 0.0050), epiandrosterone sulfate (OR = 0.74, 95% CI: 0.61–0.89, *p* = 0.0018), phenylalanylserine (OR = 0.73, 95% CI: 0.59–0.91, *p* = 0.0057), and X-12680 (OR = 0.59, 95% CI: 0.40–0.89, *p* = 0.0112). Specifically, the robustness and validity of these associations were further corroborated through various MR models including Weighted Median (WM), MR-Egger, Simple Mode, and Weighted Mode, which demonstrated consistent direction and magnitude in their estimates (Figure 3). No significant heterogeneity was detected as indicated by Cochran Q-derived *p* values, and intercepts from MR-Egger suggested the absence of horizontal pleiotropy (Table 1). In addition, LOO analysis ensured that no high-influence SNPs biased the pooled effect estimates (Supplementary Figure S1). As a result, these eight metabolites have been identified as potential candidates for metabolites implicated in the pathogenesis of AD, warranting further investigation. We also visually judged the validity of the results through funnel plots (Supplementary Figure S2). Specifically, funnel plots for 2 − aminobutyrate, pyruvate, androsterone sulfate, 1 − stearoylglycerophosphocholine, phenylalanylserine, ibuprofen, X − 11,478, and epiandrosterone sulfate, were relatively symmetric. Some funnel plots of metabolites looked somewhat skewed, including lysine, serotonin (5HT), isovalerylcarnitine, docosapentaenoate, and so on, suggesting some kind of bias existing in the MR estimates. Some funnel plots looked discrete, with the points all over the place, including the funnel plots for mannitol, 7-alpha-hydroxy-3-oxo-cholestenoate, X-12039, X-12680, and so on, suggesting the corresponding MR estimates might be biased.

**Table 1:** Supplementary and sensitivity analyses for genetic association between blood metabolites and Alzheimer’s disease.

Metabolites	*N*	MR analysis			Heterogeneity		Pleiotropy	
		Method	OR (95%CI)	*p*-value	Q	*p*-value	Intercept	*p*-value
Amino acid								
Lysine	19	IVW	0.27 (0.10–0.73)	0.0096	18.65	0.41	0.00	0.98
		WM	0.46 (0.11–1.90)	0.2835				
		MR Egger	0.30 (0.00–122.75)	0.6989				
		Simple mode	0.36 (0.03–4.75)	0.4457				
		Weighted mode	0.49 (0.06–3.87)	0.5096				
Serotonin (5HT)	15	IVW	0.58 (0.37–0.92)	0.0197	17.99	0.21	−0.01	0.50
		WM	0.69 (0.38–1.23)	0.2050				
		MR Egger	0.81 (0.29–2.23)	0.6835				
		Simple mode	0.59 (0.24–1.43)	0.2633				
		Weighted mode	0.65 (0.32–1.33)	0.2548				
2-aminobutyrate	42	IVW	0.54 (0.34–0.86)	0.0100	38.29	0.59	0.00	0.65
		WM	0.67 (0.34–1.32)	0.2482				
		MR Egger	0.73 (0.19–2.87)	0.6581				
		Simple mode	0.70 (0.18–2.68)	0.6080				
		Weighted mode	0.72 (0.23–2.23)	0.5692				
Phenol sulfate	13	IVW	0.61 (0.46–0.83)	0.0013	8.20	0.77	−0.02	0.28
		WM	0.72 (0.48–1.08)	0.1072				
		MR Egger	1.05 (0.40–2.80)	0.9195				
		Simple mode	0.38 (0.19–0.77)	0.0197				
		Weighted mode	0.75 (0.46–1.22)	0.2628				
Isovalerylcarnitine	21	IVW	0.71 (0.52–0.97)	0.0295	14.26	0.82	−0.01	0.09
		WM	0.82 (0.53–1.29)	0.3968				
		MR Egger	1.16 (0.62–2.15)	0.6464				
		Simple mode	0.87 (0.39–1.92)	0.7311				
		Weighted mode	0.90 (0.51–1.57)	0.7147				
Pyruvate	13	IVW	0.59 (0.38–0.90)	0.0155	13.09	0.36	0.00	0.96
		WM	0.66 (0.35–1.24)	0.1970				
		MR Egger	0.57 (0.16–1.99)	0.3969				
		Simple mode	0.39 (0.14–1.08)	0.0949				
		Weighted mode	0.60 (0.27–1.34)	0.2335				
Mannitol	11	IVW	0.83 (0.70–0.97)	0.0197	9.62	0.47	0.01	0.63
		WM	0.81 (0.65–1.01)	0.0649				
		MR Egger	0.75 (0.51–1.12)	0.1994				
		Simple mode	0.74 (0.50–1.10)	0.1684				
		Weighted mode	0.76 (0.56–1.04)	0.1202				
Lipid								
Glycodeoxycholate	6	IVW	1.22 (1.03–1.45)	0.0244	5.80	0.33	0.05	0.22
		WM	1.13 (0.90–1.40)	0.2977				
		MR Egger	0.74 (0.37–1.48)	0.4495				
		Simple mode	1.11 (0.81–1.52)	0.5395				
		Weighted mode	1.12 (0.88–1.42)	0.3939				
Androsterone sulfate	20	IVW	0.87 (0.76–0.99)	0.0312	27.10	0.10	0.01	0.19
		WM	0.82 (0.71–0.95)	0.0075				
		MR Egger	0.80 (0.67–0.95)	0.0210				
		Simple mode	1.11 (0.81–1.51)	0.5242				
		Weighted mode	0.82 (0.72–0.94)	0.0112				
Propionylcarnitine	28	IVW	1.63 (1.04–2.56)	0.0345	24.54	0.60	0.00	0.61
		WM	1.71 (0.88–3.31)	0.1146				
		MR Egger	2.01 (0.80–5.05)	0.1495				
		Simple mode	2.38 (0.73–7.74)	0.1617				
		Weighted mode	1.76 (0.75–4.13)	0.2059				
Docosapentaenoate(n3 DPA; 22:5n3)	8	IVW	2.08 (1.30–3.32)	0.0022	7.85	0.35	−0.01	0.64
		WM	1.93 (1.05–3.55)	0.0329				
		MR Egger	2.92 (0.70–12.17)	0.1915				
		Simple mode	1.32 (0.48–3.67)	0.6098				
		Weighted mode	3.01 (1.19–7.64)	0.0534				
1-stearoylglycerophospho-choline	12	IVW	0.49 (0.30–0.81)	0.0050	5.54	0.90	0.00	0.96
		WM	0.49 (0.25–0.95)	0.0334				
		MR Egger	0.50 (0.15–1.64)	0.2804				
		Simple mode	0.40 (0.14–1.12)	0.1083				
		Weighted mode	0.41 (0.14–1.20)	0.1334				
Epiandrosterone sulfate	12	IVW	0.74 (0.61–0.89)	0.0018	15.03	0.18	0.01	0.34
		WM	0.71 (0.57–0.87)	0.0013				
		MR Egger	0.66 (0.49–0.88)	0.0196				
		Simple mode	0.61 (0.42–0.89)	0.0269				
		Weighted mode	0.70 (0.58–0.85)	0.0046				
7-alpha-hydroxy-3-oxo-4-cholestenoate (7-Hoca)	12	IVW	0.45 (0.21–0.97)	0.0405	4.95	0.93	−0.01	0.57
		WM	0.49 (0.19–1.26)	0.1408				
		MR Egger	0.82 (0.10–7.08)	0.8640				
		Simple mode	0.72 (0.15–3.53)	0.6980				
		Weighted mode	0.72 (0.14–3.65)	0.7036				
Nucleotide								
Urate	20	IVW	3.22 (1.23–8.42)	0.0169	23.10	0.23	−0.01	0.58
		WM	6.02 (1.79–20.31)	0.0038				
		MR Egger	9.62 (0.20–466.44)	0.2678				
		Simple mode	5.59 (0.65–47.76)	0.1322				
		Weighted mode	7.92 (1.18–53.23)	0.0466				
Peptide								
Gamma-glutamylglutamine	21	IVW	0.44 (0.21–0.91)	0.0279	34.13	0.03	0.01	0.61
		WM	0.31 (0.13–0.72)	0.0071				
		MR Egger	0.29 (0.05–1.72)	0.1875				
		Simple mode	0.38 (0.10–1.42)	0.1645				
		Weighted mode	0.49 (0.14–1.72)	0.2771				
Leucylalanine	12	IVW	0.77 (0.59–0.99)	0.0455	21.77	0.03	−0.01	0.40
		WM	0.86 (0.65–1.14)	0.2929				
		MR Egger	0.94 (0.56–1.59)	0.8276				
		Simple mode	1.02 (0.60–1.73)	0.9450				
		Weighted mode	0.89 (0.66–1.22)	0.4964				
Phenylalanylserine	14	IVW	0.73 (0.59–0.91)	0.0057	17.97	0.16	−0.01	0.64
		WM	0.79 (0.58–1.07)	0.1321				
		MR Egger	0.80 (0.52–1.24)	0.3399				
		Simple mode	0.52 (0.30–0.90)	0.0353				
		Weighted mode	0.80 (0.56–1.14)	0.2404				
Xenobiotics								
Ibuprofen	113	IVW	0.95 (0.91–1.00)	0.0306	99.57	0.79	−0.01	0.25
		WM	0.97 (0.90–1.03)	0.3075				
		MR Egger	1.02 (0.90–1.15)	0.7764				
		Simple mode	0.99 (0.85–1.15)	0.8988				
		Weighted mode	0.99 (0.87–1.12)	0.8791				
Unknown								
X-09789	10	IVW	0.72 (0.52–1.00)	0.0482	11.08	0.27	0.03	0.21
		WM	0.68 (0.45–1.03)	0.0688				
		MR Egger	0.36 (0.13–1.02)	0.0903				
		Simple mode	0.61 (0.32–1.18)	0.1756				
		Weighted mode	0.62 (0.36–1.06)	0.1169				
X-11478	13	IVW	1.32 (1.01–1.74)	0.0446	6.96	0.86	0.00	0.88
		WM	1.33 (0.92–1.94)	0.1338				
		MR Egger	1.28 (0.78–2.09)	0.3435				
		Simple mode	1.46 (0.86–2.48)	0.1911				
		Weighted mode	1.36 (0.90–2.05)	0.1667				
X-12029	36	IVW	0.60 (0.37–0.97)	0.0385	43.17	0.16	0.00	0.73
		WM	0.52 (0.25–1.09)	0.0822				
		MR Egger	0.53 (0.23–1.24)	0.1520				
		Simple mode	0.97 (0.24–4.00)	0.9669				
		Weighted mode	0.51 (0.27–0.98)	0.0494				
X-12039	13	IVW	0.78 (0.61–0.99)	0.0371	18.32	0.11	0.00	0.80
		WM	0.92 (0.69–1.22)	0.5689				
		MR Egger	0.82 (0.51–1.31)	0.4220				
		Simple mode	1.00 (0.64–1.55)	0.9921				
		Weighted mode	0.98 (0.62–1.53)	0.9141				
X-12680	12	IVW	0.59 (0.40–0.89)	0.0112	5.69	0.89	−0.01	0.61
		WM	0.63 (0.36–1.08)	0.0909				
		MR Egger	0.79 (0.25–2.46)	0.6895				
		Simple mode	0.60 (0.24–1.53)	0.3083				
		Weighted mode	0.59 (0.25–1.41)	0.2606				
X-12850	14	IVW	0.75 (0.58–0.96)	0.0248	7.27	0.89	0.00	0.76
		WM	0.76 (0.54–1.08)	0.1218				
		MR Egger	0.81 (0.46–1.41)	0.4666				
		Simple mode	0.76 (0.44–1.31)	0.3361				
		Weighted mode	0.76 (0.46–1.26)	0.3122				

*N*, number of single nucleotide polymorphisms; IVW, Inverse variance weighted; WM, Weighted median; OR, odds ratio; 95% CI, 95% confidence interval.

### 3.2 Replication and meta-analysis with the FinnGen data

**Figure 4:**
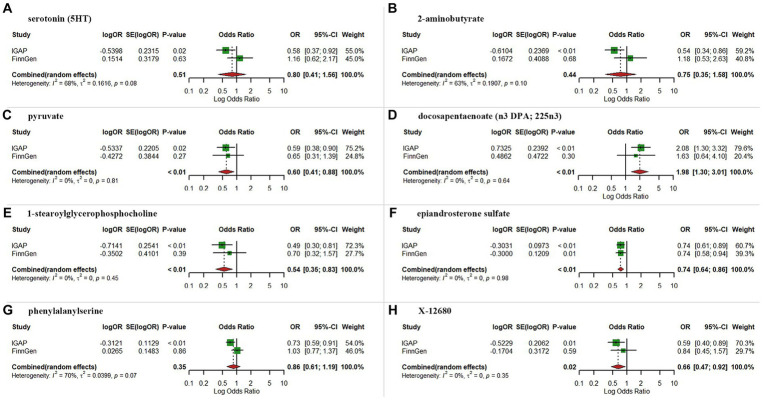
Meta-analysis of suggestive significantly associated (IVW derived *p* < 0.05) between metabolites and Alzheimer’s disease. **(A)** serotonin (5HT); **(B)** 2-aminobutyrate; **(C)**, pyruvate; **(D)** docosapentaenoate; **(E)** 1-stearoylglycerophosphocholine; **(F)** epiandrosterone sulfate; **(G)** phenylalanylserine; **(H)** X-12680; OR, odds ratio; 95% CI, 95% confidence interval.

As expected, replication analysis utilizing AD GWAS dataset from the FinnGen consortium showed analogous trends for certain metabolites (Figure 4). A meta-analysis of data from IGAP and FinnGen further indicated higher levels of pyruvate (OR = 0.60, 95% CI: 0.41–0.88, *p* < 0.01), 1-stearoylglycerophosphocholine (OR = 0.54, 95% CI: 0.35–0.83, *p* < 0.01), epiandrosterone sulfate (OR = 0.74, 95% CI: 0.64–0.86, *p* < 0.01) and X-12680 (OR = 0.66, 95% CI: 0.47–0.92, *p* = 0.02) were linked to a lower AD risk, and higher levels of docosapentaenoate (n3 DPA; 22:5n3) (OR = 1.98, 95% CI:1.30–3.01, *p* < 0.01) were linked to a higher AD risk. However, the results of meta-analysis did not yield statistically significant effects for serotonin (5HT), 2-aminobutyrate, and phenylalanylserine.

### 3.3 Genetic correlation and direction validation

LDSC analysis revealed an insignificant genetic correlation between AD and pyruvate (R_g_ = 0.1840, se = 0.1649, *p* = 0.2644), docosapentaenoate (n3 DPA; 22:5n3) (R_g_ = −0.0277, se = 0.2615, *p* = 0.9157), 1-stearoylglycerophosphocholine (R_g_ = 0.1883, se = 0.3617, *p* = 0.6027) and epiandrosterone sulfate (R_g_ = 0.0249, se = 0.3119, *p* = 0.9364). This implies that the absence of confounding shared genetic components in MR estimates. Additionally, there was no indication of reverse causation between metabolites and AD according to the Steiger test results (Table 2).

**Table 2:** Steiger direction test from blood metabolites to Alzheimer’s disease.

Exposure	Pyruvate	Docosapentaenoate (n3 DPA; 22:5n3)	1-stearoylglycerophosphocholine	Epiandrosterone sulfate
Direction	TRUE	TRUE	TRUE	TRUE
Steiger P	1.19E-58	9.00E-55	7.23E-67	1.15E-155

### 3.4 Confounding analysis and MVMR

Although sensitivity analysis was conducted to exclude SNPs that deviated from the estimated values in this study, a manual examination of the second traits (educational attainment, HDL cholesterol, smoking, BMI, diastolic blood pressure, and systolic blood pressure) of the metabolite-associated SNPs was conducted. Looking over the Phenoscanner, we found that SNPs associated with pyruvate, epiandrosterone sulfate and X-12680 were not associated with any of the modifiable risk factors for AD. For docosapentaenoate (n3 DPA; 22:5n3), one SNP (rs174538) was associated with HDL cholesterol-related phenotypes. Similar for 1-stearoylglycerophosphocholine, the SNP (rs4082919) was associated with HDL cholesterol-related phenotypes (Supplementary Table S3). As anticipated, the associations remained suggestive significant for docosapentaenoate (n3 DPA; 22:5n3) (OR = 2.24, 95% CI: 1.33–3.79, *p* = 0.0026), and 1-stearoylglycerophosphocholine (OR = 0.49, 95% CI: 0.29–0.82, *p* = 0.0068), even after these SNPs were eliminated.

**Figure 5:**
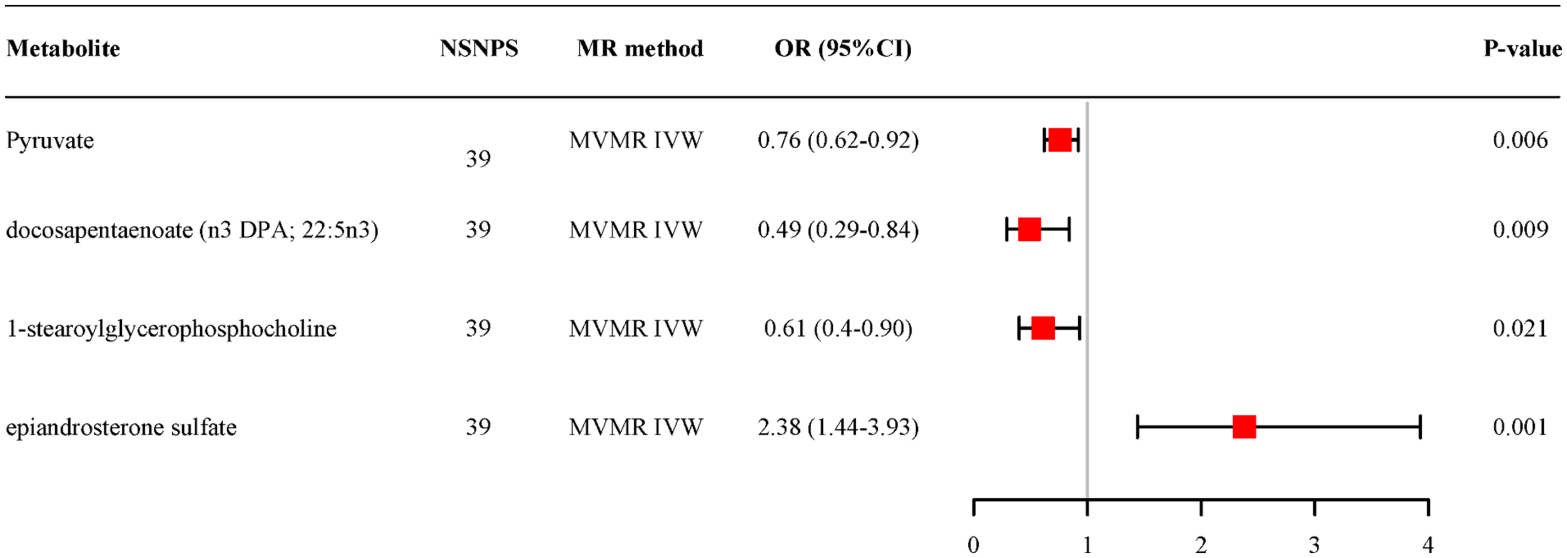
Multivariable MR analysis of the final identified blood metabolites. 95% CI, 95% confidence interval; IVW, inverse variance weighted; MVMR, Multivariable Mendelian randomization; OR, odds ratio; 95% CI, 95% confidence interval; NSNPS, number of single nucleotide polymorphisms.

MVMR analysis based on IVW for all candidate metabolites provided evidence of separate effects for pyruvate (OR = 0.76, 95% CI: 0.62–0.92, *p* = 0.006), docosapentaenoate (n3 DPA; 22:5n3) (OR = 0.49, 95% CI: 0.29–0.84, *p* = 0.009), 1-stearoylglycerophosphocholine (OR = 0.61, 95% CI:0.40–0.90, *p* = 0.021) and epiandrosterone sulfate (OR = 2.38, 95% CI: 1.44–3.93, *p* = 0.001) on AD (Figure 5). And the adjusted effects align with those observed in the univariable MR analyses.

### 3.5 Metabolic pathway analysis

Through analysis of four established metabolites, we identified six metabolic pathways potentially implicated in AD pathogenesis (all *p* < 0.1) (Supplementary Table S4). These pathways include involve Cysteine and methionine metabolism, Citrate cycle (TCA cycle), Pyruvate metabolism, Glycolysis/Gluconeogenesis, Alanine, aspartate and glutamate metabolism, and Glyoxylate and dicarboxylate metabolism, suggesting their potential involvement in the biological processes underlying AD development. Notably, pyruvate is a constituent of all identified metabolic pathways, underscoring its pivotal role in AD’s development.

### 3.6 Replication analysis with the EADB data

We conducted an extra replication analysis of our findings using the data from EADB stage 1. For the metabolites identified in the replication and meta-analysis with the FinnGen data, only two blood metabolites were confirmed in the replicative analysis, including epiandrosterone sulfate (OR = 0.85, 95% CI: 0.78–0.93, *p* = 6e−04) and X-12680 (OR = 0.79, 95% CI: 0.66–0.95, *p* = 0.0104). Pyruvate, docosapentaenoate, and 1-stearoylglycerophosphocholine showed no suggestive significant associations with AD. The replicative results were presented in Supplementary Figure S3.

Taken together, we found that epiandrosterone sulfate was the most promising metabolite associated with AD development, with consistent estimates across three MR studies, passing all the quality control of sensitivity analysis, as well as a relatively symmetric funnel plot. The following relatively promising metabolite is X-12680, for which the estimated associations were consistent across all MR studies, and the sensitivity analysis confirmed the robustness of the results, whereas the funnel plot was discrete. For pyruvate and 1-stearoylglycerophosphocholine, the associations with AD risk were not confirmed in the EADB dataset, with stable results in sensitivity analyses and relatively symmetric funnel plots. For docosapentaenoate, the association with AD was also only confirmed in IGAP and FinnGen datasets, and the funnel plot was skewed.

## 4 Discussion

In the current work, we employed a stringent MR design to examine the genetic associations between 486 blood metabolites and AD by integrating two independent AD GWAS datasets. We determined that genetic predisposition to high levels of pyruvate, 1-stearoylglycerophosphocholine, epiandrosterone sulfate, and X-12680 were associated with a reduced risk of AD, whereas a genetic predisposition to high levels of docosapentaenoate (n3 DPA; 22:5n3) increased risk of AD. MVMR estimates suggested that these known metabolites can directly affect AD in independent of other metabolites. Subsequently, our study identified 6 metabolic pathways potentially involved in AD’s biological mechanisms. In addition, we also replicated our findings with EADB data. We found that epiandrosterone sulfate and X-12680 were confirmed in the replicative analysis, whereas pyruvate, docosapentaenoate, and 1-stearoylglycerophosphocholine failed to replicate the associations in the EADB dataset. To our knowledge, this MR study is the first to utilize the most comprehensive blood metabolite GWAS data to explore genetic association with AD to date.

In the National Institute on Aging and Alzheimer’s Association Research Framework, the definition of AD has evolved from a syndromal to a biological construct centered on biomarkers categorized as β-amyloid deposition, pathologic tau (Jack et al., 2018). The conventional method of identifying *in vivo* biomarkers is typically employed after the manifestation of clinical symptoms, and is constrained by its high expenses and invasive nature. From this, effective interventions for early detection may substantially decrease underdiagnosis and potentially offer patients extended time by delaying the progression of AD. Blood metabolites, with the noninvasive detection, amenability to intervention, and the capability of many to traverse the blood–brain barrier, are regarded as valuable markers to fulfill this goal (Enche et al., 2017; Lord et al., 2021). An increasing amount of evidence indicates that abnormalities in blood metabolism could be related to the degree of neuropathology of AD and affected the eventual manifestation of symptoms (Varma et al., 2018; Kuehn, 2020; Peng et al., 2020; Yu et al., 2022). Nevertheless, the uncertain link between these abnormalities and AD limits their utility in early detection. Our MR study aims to elucidate this relationship, potentially guiding future AD screening and treatment strategies.

In this study, we observed that susceptibility to elevated epiandrosterone sulfate levels and X-12680 levels are linked to a reduced AD risk in all three MR-studies. Regarding epiandrosterone sulfate, it is among the metabolites associated with the metabolism of androgens. Previous researches have elucidated the neuroprotective function of androgen supplementation in mitigating amyloid beta deposition and tau hyperphosphorylation (Gouras et al., 2000; Pike et al., 2009). The sex difference in AD risk has also been reported, and women exhibit a greater lifetime susceptibility (Nebel et al., 2018). Again, our research revealed that individuals who possess a genetic predisposition towards heightened levels of epiandrosterone sulfate exhibited a protective effect against AD. This assertion was substantiated in a subsequent study investigating the correlation between sex hormones and AD risk. However, caution is advised in clinical practice, as a Phe-MR analysis by Sun et al. suggested potential detrimental effects of epiandrosterone sulfate on respiratory and digestive diseases (Sun et al., 2022). Thus, while epiandrosterone sulfate may hold promise as a therapeutic target for AD, careful consideration of potential adverse side effects is essential. Regrettably, though X-12680 has represented potential protective effects on AD in the study, the functional structure of the metabolite was unknown and it has not been previously reported to be associated with AD risk. Future studies still need to increase the exploration of unknown metabolites.

Two other metabolites, pyruvate and 1-stearoylglycerophosphocholine, were also identified with potential protective effects on AD in IGAP-MR and FinnGen-MR. This finding aligns with several prior research studies that have also highlighted the opportunity of pyruvate in the management of AD (Isopi et al., 2015; Wang et al., 2015). For a long time, abnormal brain metabolism has been considered a pivotal pathophysiological characteristic of AD, preceding cognitive dysfunction by decades (Reiman et al., 1996; Jack et al., 2010; Cunnane et al., 2011). Neurons, which are central to brain function, heavily rely on oxidative metabolism and exhibit a preference for the uptake of lactate, which is subsequently metabolized into pyruvate by the enzyme lactate dehydrogenase (LDH) for their energy requirements (Bolanos et al., 2010; Gray et al., 2014). On the other hand, according to the astrocyte–neuron lactate shuttle (ANLS) hypothesis (Brooks, 2018), lactate is shuttled from glial cells to neurons, converting to pyruvate and entering the TCA cycle to produce ATP. It follows that an increase in pyruvate may provide an important fuel for compromised neurons. Second, pyruvate also exhibits anti-inflammatory and antioxidant properties (Tauffenberger et al., 2019), and these effects give rise to a microenvironment that is unfavorable for the development of AD (Das, 2006). At last, pyruvate can contribute to reducing body fat and alleviating insulin resistance (Nellemann et al., 2012; Kang et al., 2021). Adequate pyruvate levels may indirectly reduce fat synthesis, ameliorating obesity and lowering the risk of AD. In summary, pyruvate emerges as a metabolite with significant potential for AD prevention and treatment. For 1-stearoylglycerophosphocholine, there exists a scarcity of research pertaining to the role of 1-stearoylglycerophosphocholine in AD. One study has shown that 1-stearoylglycerophosphocholine is a natural product with potential PPAR-gamma activity, which plays hypoglycemic and hypolipidemic effect with lower liver toxicity and cardiotoxicity in db/db mice (Ma et al., 2021). Another study also reported that 1-stearoylglycerophosphocholine can improve HFD-induced obese mouse model, and may be an ideal product for preventing and treating obesity as well as associated metabolic disorders (Han et al., 2021). Given the established correlation between obesity, type 2 diabetes mellitus (T2DM), and AD risk, we hypothesize that 1-stearoylglycerophosphocholine may control AD progression by mitigating obesity and diabetes. In addition, 1-stearoylglycerophocholine, as a type of lysophosphatidylcholine (lysoPCs). Several studies have reported a reduction in lysoPC levels within the brain, CSF, and plasma in individuals suffering from AD (Schmerler et al., 2012; González-Domínguez et al., 2014). Lin et al. further found that seven lysoPCs were decreased simultaneously in AD plasma (Lin et al., 2017), which also seems to suggest an association between 1-steroylglycophorine and AD risk. Despite these, considering the associations of pyruvate and 1-stearoylglycerophosphocholine with AD failed to replicate in the EADB dataset, future investigation is warranted to further verify their roles in AD development.

Notably, the association of docosapentaenoate (n3 DPA; 22:5n3) with the risk of AD remains highly controversial. Prior researches have explored the beneficial role of AD in neurological function, reducing cardiovascular events, and diabetes risk (Kaur et al., 2011; Mozaffarian and Wu, 2012; Igarashi et al., 2013). Kelly L et al. further found that the administration of n-3 DPA to aged rats for a duration of 56 days resulted in the manifestation of neuroprotective effects (Kelly et al., 2011). This work showed that n-3 DPA can attenuate the age-associated increases in caspase 3 activity and microglial activation, while simultaneously restoring cognitive function and synaptic plasticity in aged rat models. However, docosapentaenoate might pose a risk for AD in IGAP-MR and FinnGen-MR, which is contrary to the previously reported facts. This discrepancy may be attributed to variations in sample size and population demographics in our study. Consequently, a more comprehensive and meticulous examination is warranted to elucidate the association between docosapentaenoate and AD.

Despite these, considering the existence of blood–brain barrier (BBB), the biology of blood metabolites involved in AD development still warrants further investigation. Some blood metabolites, like pyruvate, could pass through BBB and exert the impact on the central nervous system (CNS), consequently influence the development of AD. However, some blood metabolites might be blocked by BBB, which would limit the effects on CNS, for which we hypothesize genetically determined levels of metabolites might influence the development of AD. Future studies should focus on the relationship between CNS metabolism and AD to validate this hypothesis.

Our study presents several strengths. Firstly, a significant advantage of this MR study lies in its comprehensive coverage of a wide array of blood metabolites. To be specific, a total of 486 metabolites were meticulously incorporated for MR analysis, marking it as the most comprehensive and systematic investigation to date of the metabolic profiles associated with AD. Secondly, a rigorous MR analysis was conducted to effectively mitigate the inherent limitations identified in prior studies, encompassing the issues of reverse association and confounding interference. Thirdly, the application of replication and meta-analyses served to reinforce the genetic impact of specific metabolites on AD. Even though estimates in the replication analysis from the FinnGen GWAS and EADB GWAS did not attain statistical significance, the steadfast alignment of effect estimates provided reassurance, suggesting their occurrence was not merely coincidental. Lastly, we evaluated the heritability of IVs and the genetic correlation between metabolites and AD using LDSC, further improving the confidence of MR Estimates.

This study necessitates the recognition of numerous constraints. One primary concern is the reliance on European population data. Since all of the GWAS data exclusively originated from European populations, necessitating further validation in diverse populations, and larger sample sizes are required to confirm findings. Additionally, we revealed that four metabolites are nominally genetically associated with AD via a two-sample MR approach. But still, this association remains theoretical, as we were unable to substantiate it mechanistically. Besides, though over 400 metabolites were tested in this work, we did not conduct multiple testing correction for the MR results as we consider that multiple testing correction might miss out potential findings. Instead, we replicated the MR results in another independent cohort and conducted a meta-analysis to obtain candidate metabolites involved in AD risk, which is similar to a previous MR analysis conducted by Cai et al. (2022). Last, despite valuable insights offered by MR analysis in understanding etiology, triangulation of evidence is needed to enhance the findings of this work. Therefore, it is imperative to validate our findings through RCTs and fundamental research prior to their clinical application.

## 5 Conclusion

In conclusion, this current MR analysis provides evidence that certain blood metabolites are involved in AD development, and suggests that epiandrosterone sulfate and X-12680 might be the most promising metabolic biomarkers for AD screening, prevention and treatment in clinical settings. These finding offer a basis and new insight for future mechanistic investigations, although further studies are needed for validation.

## Data availability statement

Publicly available datasets were analyzed in this study. Data about the blood metabolites can be found at: https://metabolomips.org/gwas/index.php?task=download. AD data from IGAP consortia can be found at: https://gwas.mrcieu.ac.uk/datasets/ieu-b-2/. AD data from FinnGen consortia can be found at: https://r9.finngen.fi/pheno/G6_ALZHEIMER. AD data from EADB consortia can be found at: https://www.ebi.ac.uk/gwas/studies/GCST90027158.

## Ethics statement

This study is based on large-scale GWAS datasets, and not individual-level data. Ethical approval is not applicable.

## Author contributions

QY: Conceptualization, Data curation, Formal analysis, Methodology, Visualization, Writing – original draft, Writing – review & editing. XH: Conceptualization, Formal analysis, Software, Writing – original draft. MY: Data curation, Supervision, Validation, Writing – review & editing. TJ: Data curation, Supervision, Validation, Writing – review & editing. BW: Formal analysis, Methodology, Validation, Writing – review & editing. ZZ: Data curation, Methodology, Validation, Writing – review & editing. FL: Conceptualization, Funding acquisition, Methodology, Supervision, Validation, Writing – review & editing.

## Footnotes

^1^ https://www.finngen.fi/fi

^2^ https://www.ebi.ac.uk/gwas/

^3^ http://www.phenoscanner.medschl.cam.ac.uk/
