# Supplementary material for: Association of genetically predicted 486 blood metabolites on the risk of Alzheimer’s disease: a Mendelian randomization study
Source: Front Aging Neurosci. 2024 Apr 12;16:1372605. doi: 10.3389/fnagi.2024.1372605 (PMC11047179; doi:10.3389/fnagi.2024.1372605)
Supplement: Supplementary file 1 [file Data_Sheet_1.docx]

Supplementary Material

# Supplementary Figures and Tables

## Supplementary Tables

**Supplementary Table S1.** List of the identification for each of the 486 blood metabolites.

**Supplementary Table S2.** Harmonization data of 485 blood metabolites and Alzheimer's disease.

**Supplementary Table S3.** Confounders identified from Phenoscanner.

**Supplementary Table S4.** Metabolic pathways with suggestive significant enrichment of blood metabolites.

## Supplementary Figures

**Supplementary Figure S1.** Forest plots for the Mendelian randomization leave-one-out analysis of the suggestive significant IVW estimates. The solid lines represent 95% confidence interval.

**Supplementary Figure S2.** Funnel plots for the suggestive significant IVW estimates between metabolites and Alzheimer's disease.

**Supplementary Figure S3.** Forest plot for the genetic association derived from IVW of the metabolites identified in the replication and meta-analysis on the risk of Alzheimer's disease in EADB stage I. EADB, the European Alzheimer & Dementia Biobank; IVW, inverse variance weighted; OR, odds ratio; 95% CI, confidence interval; NSNPS, number of single nucleotide polymorphisms.

**Supplementary Figure S1.** Forest plots for the Mendelian randomization leave-one-out analysis of the suggestive significant IVW estimates.

**
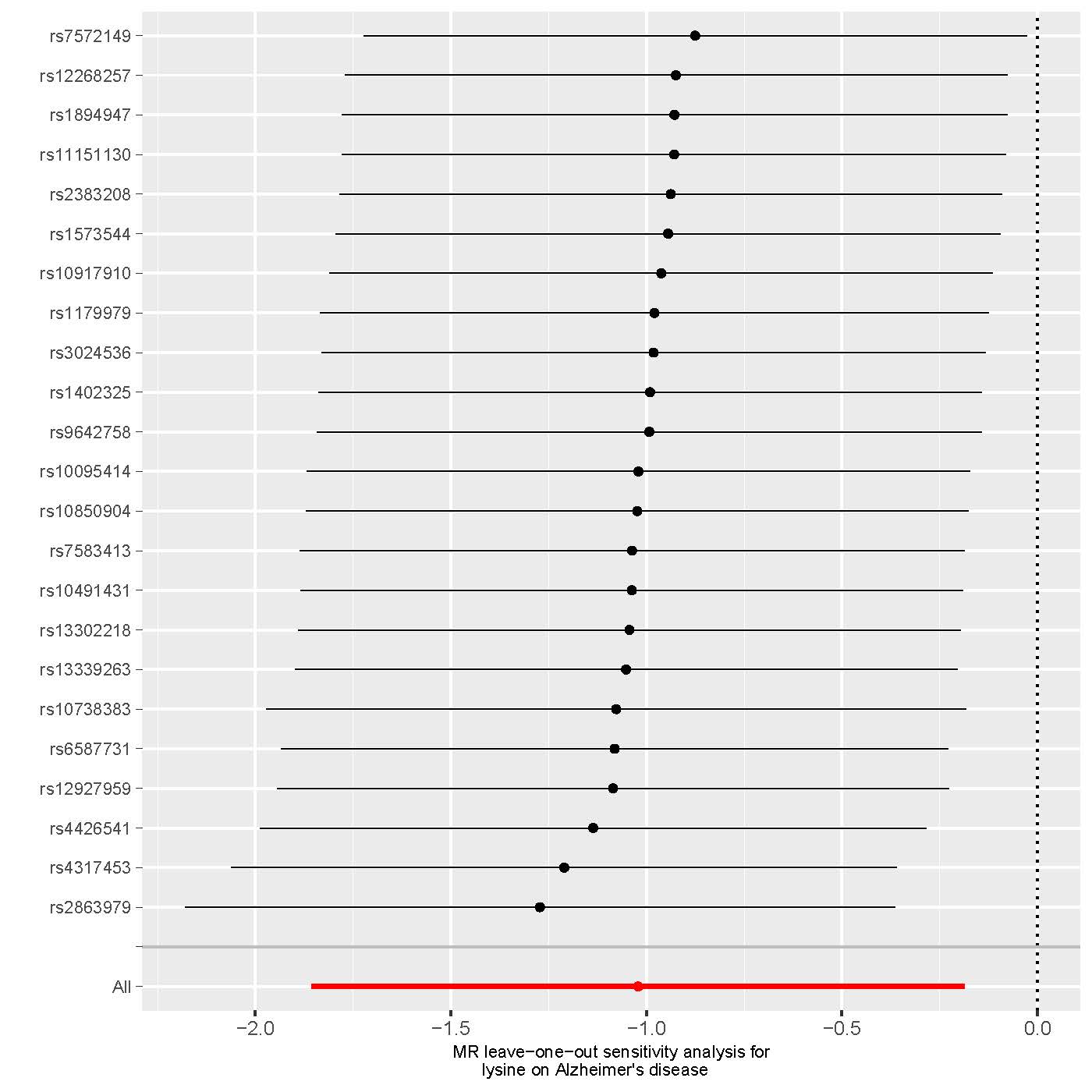

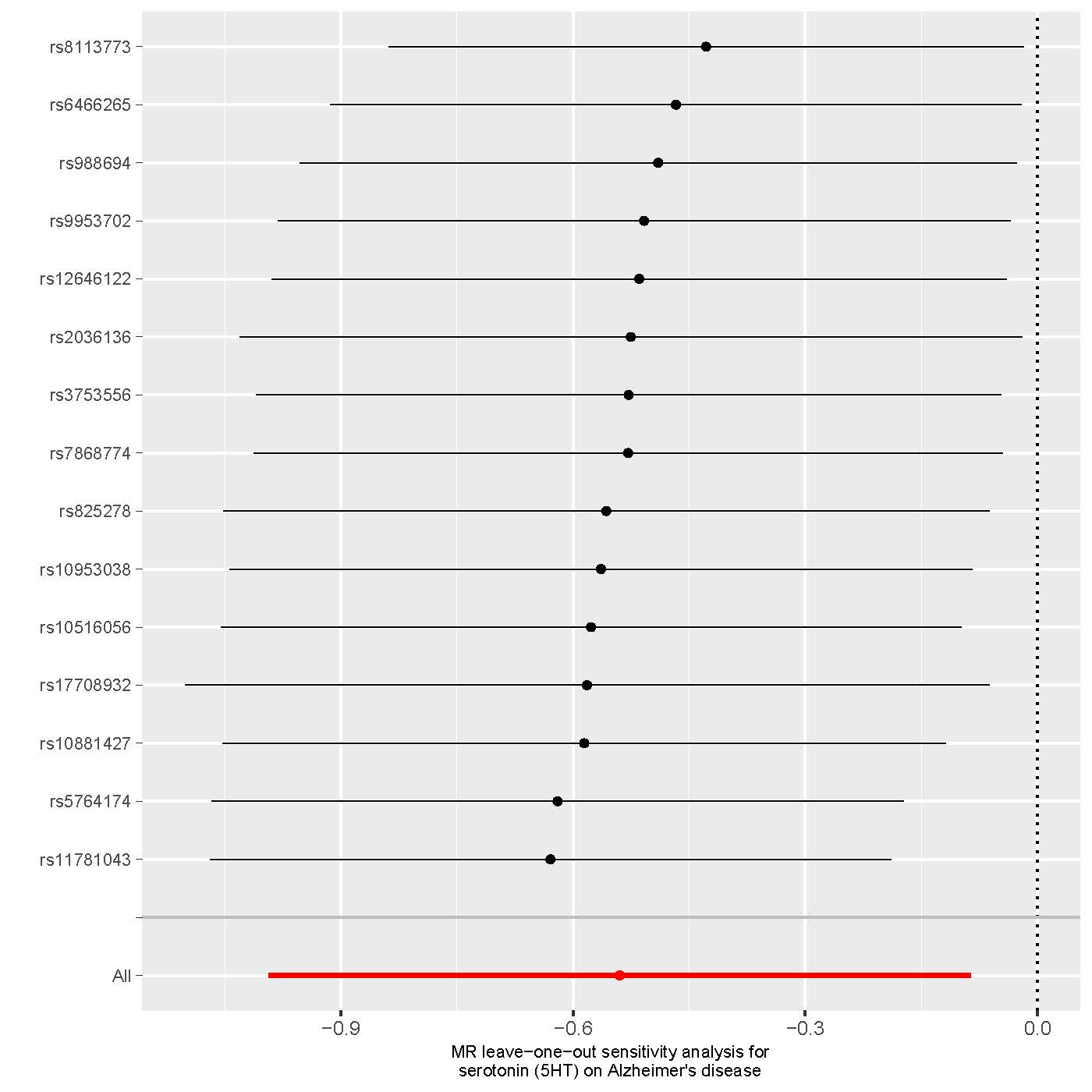
**

**
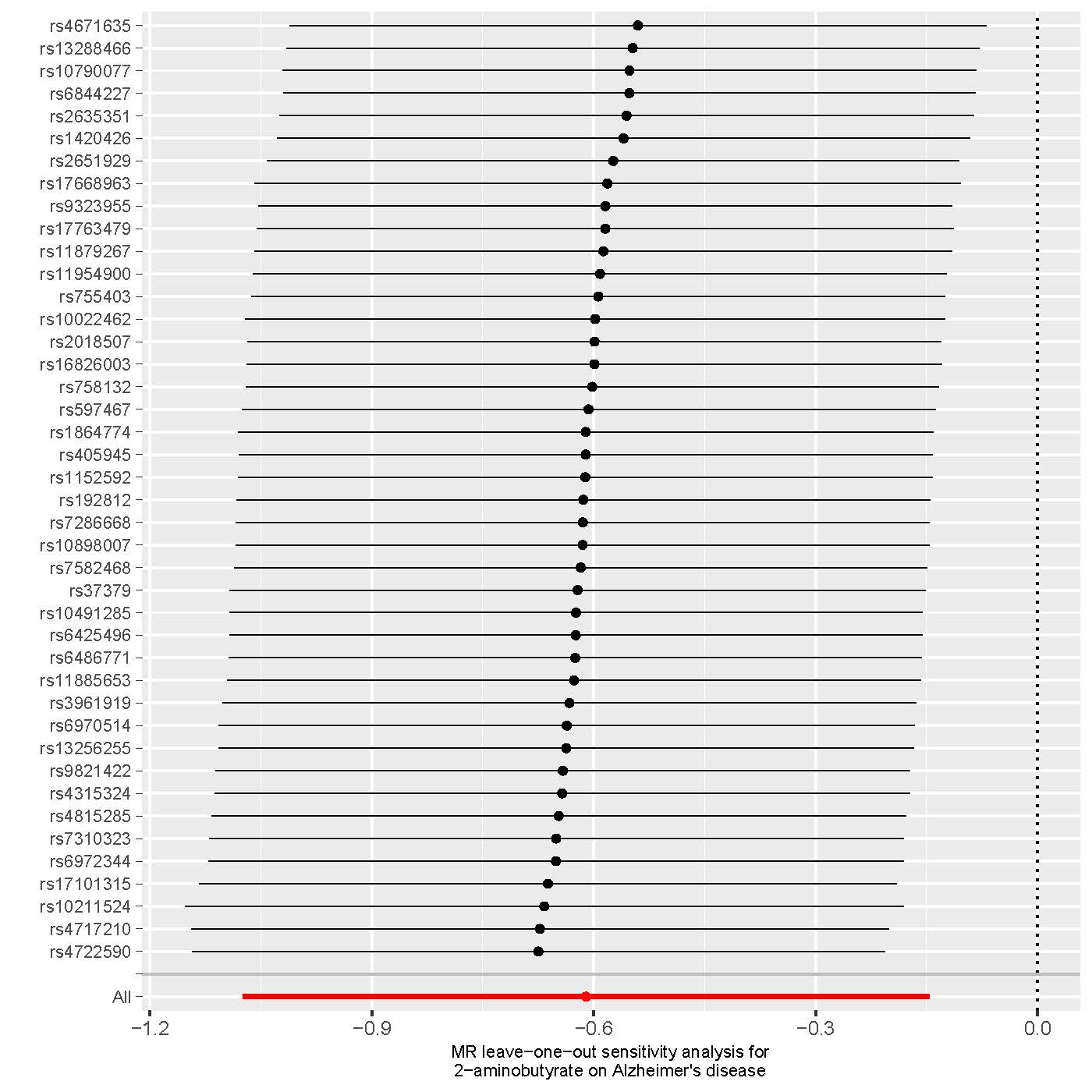

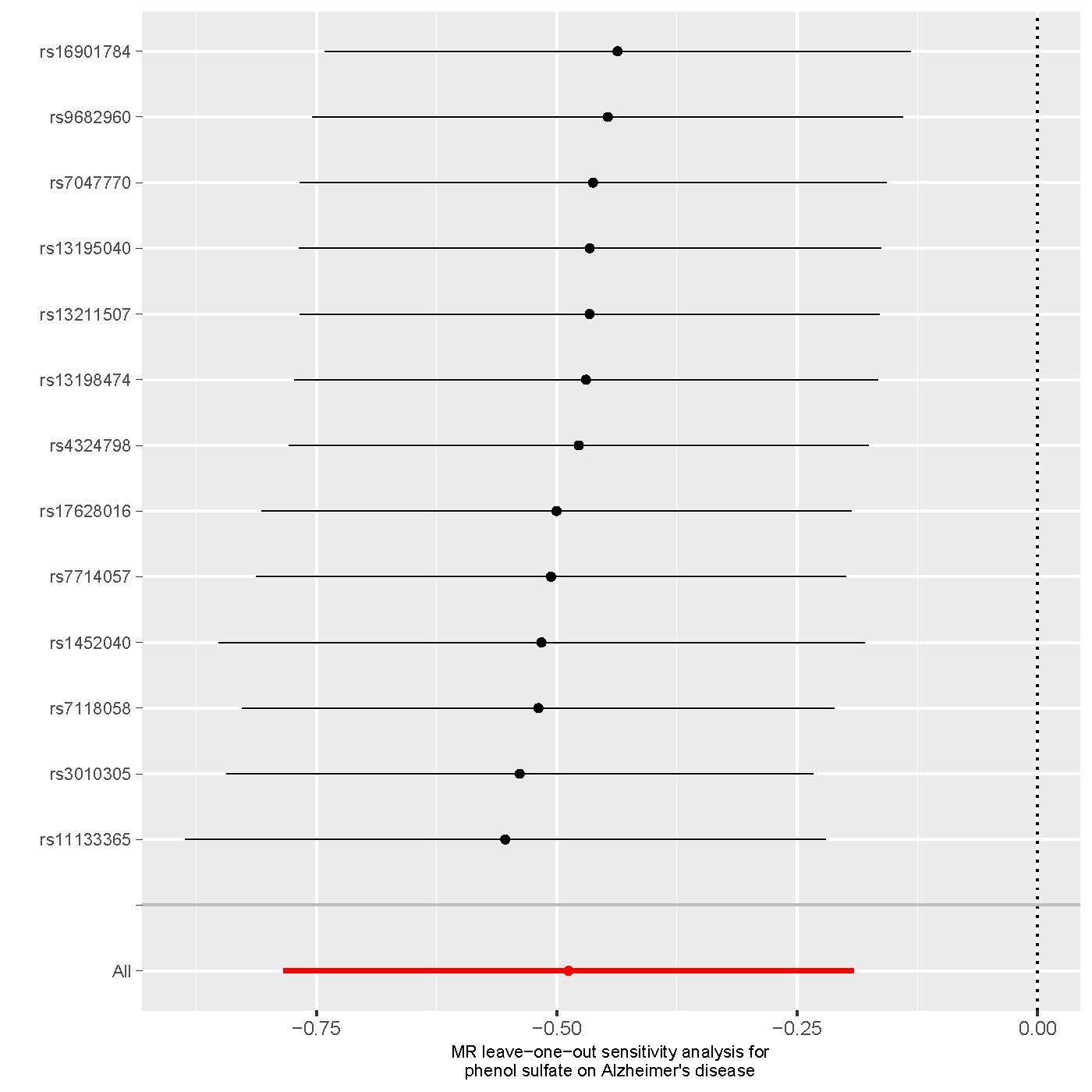
**

**
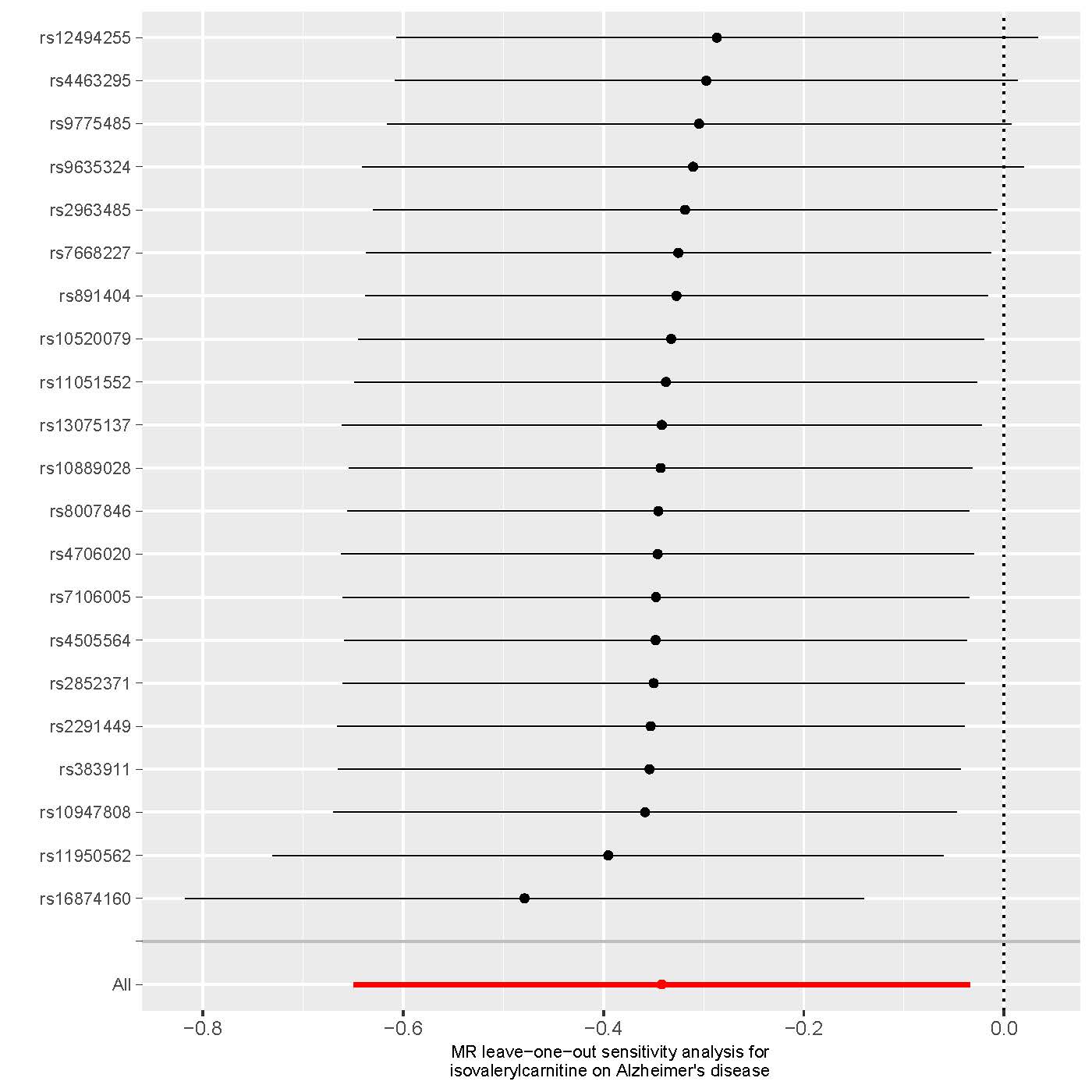

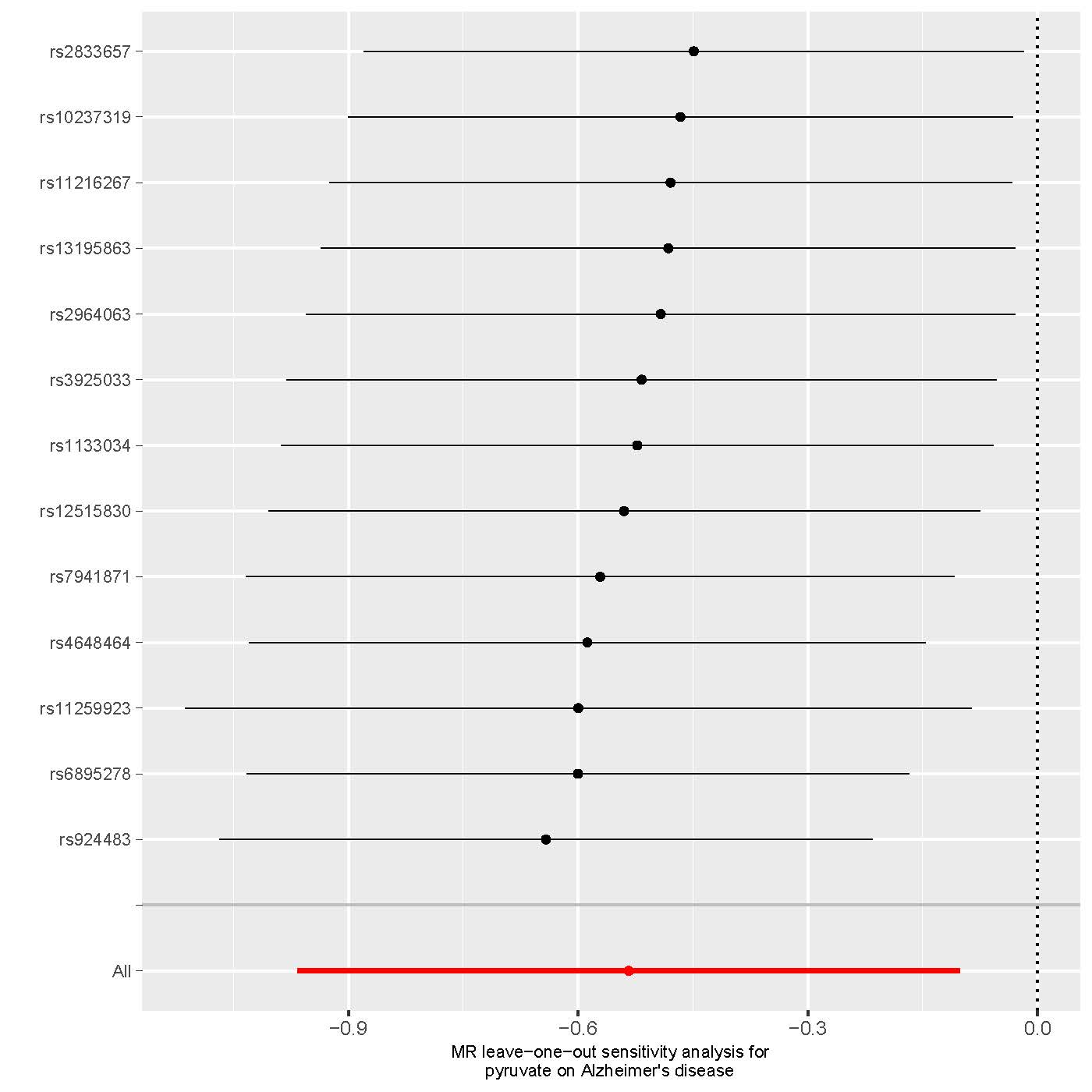
**

**
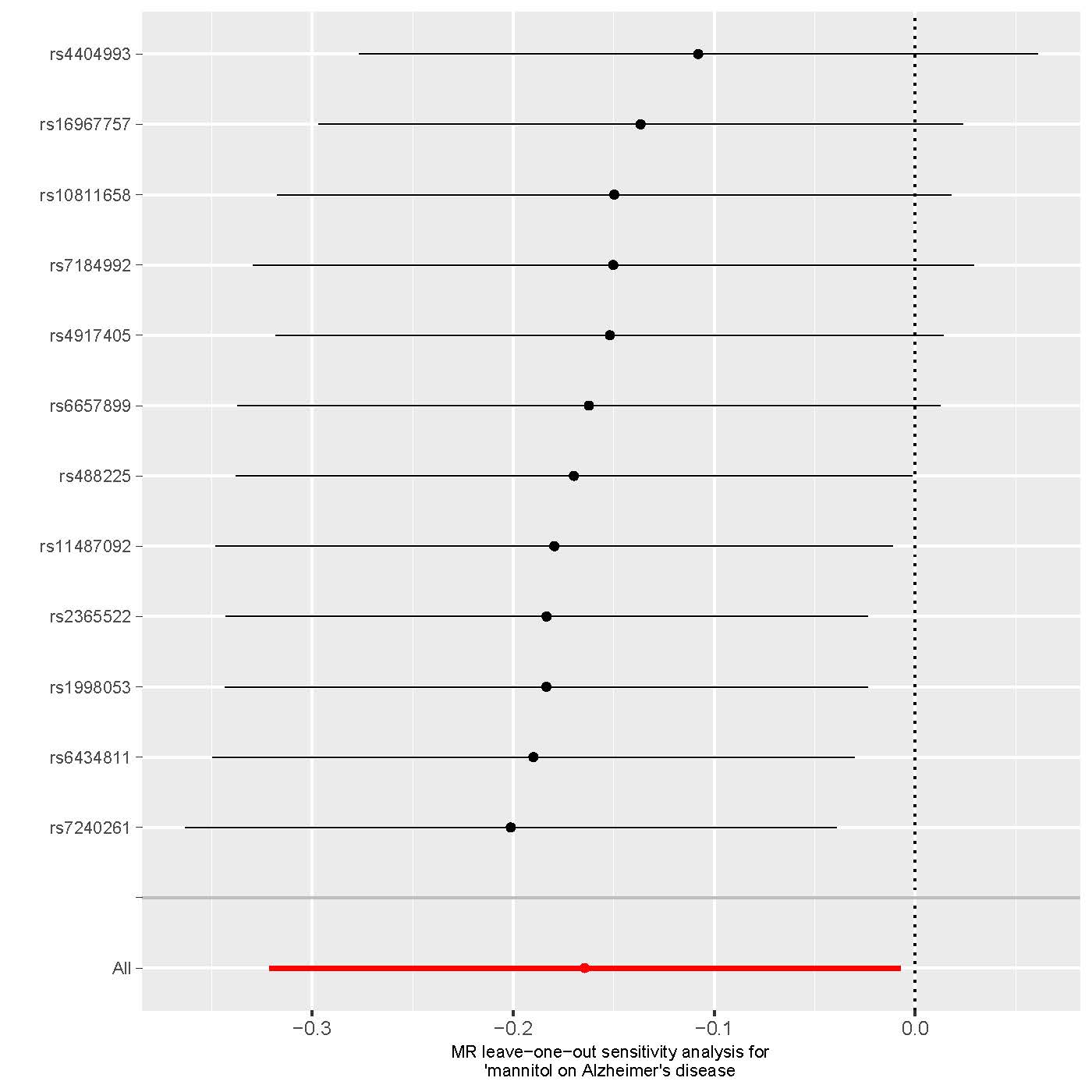

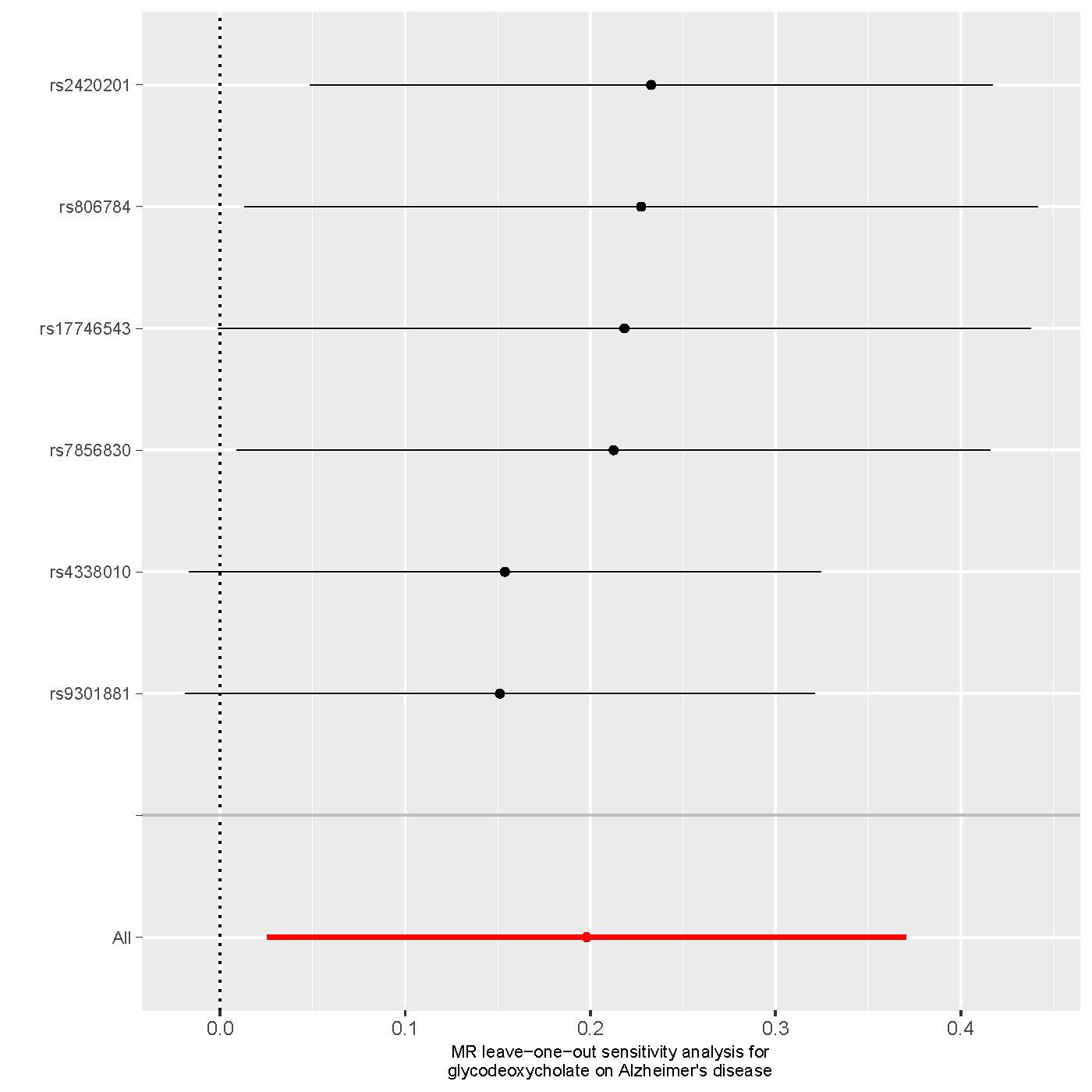
**

**
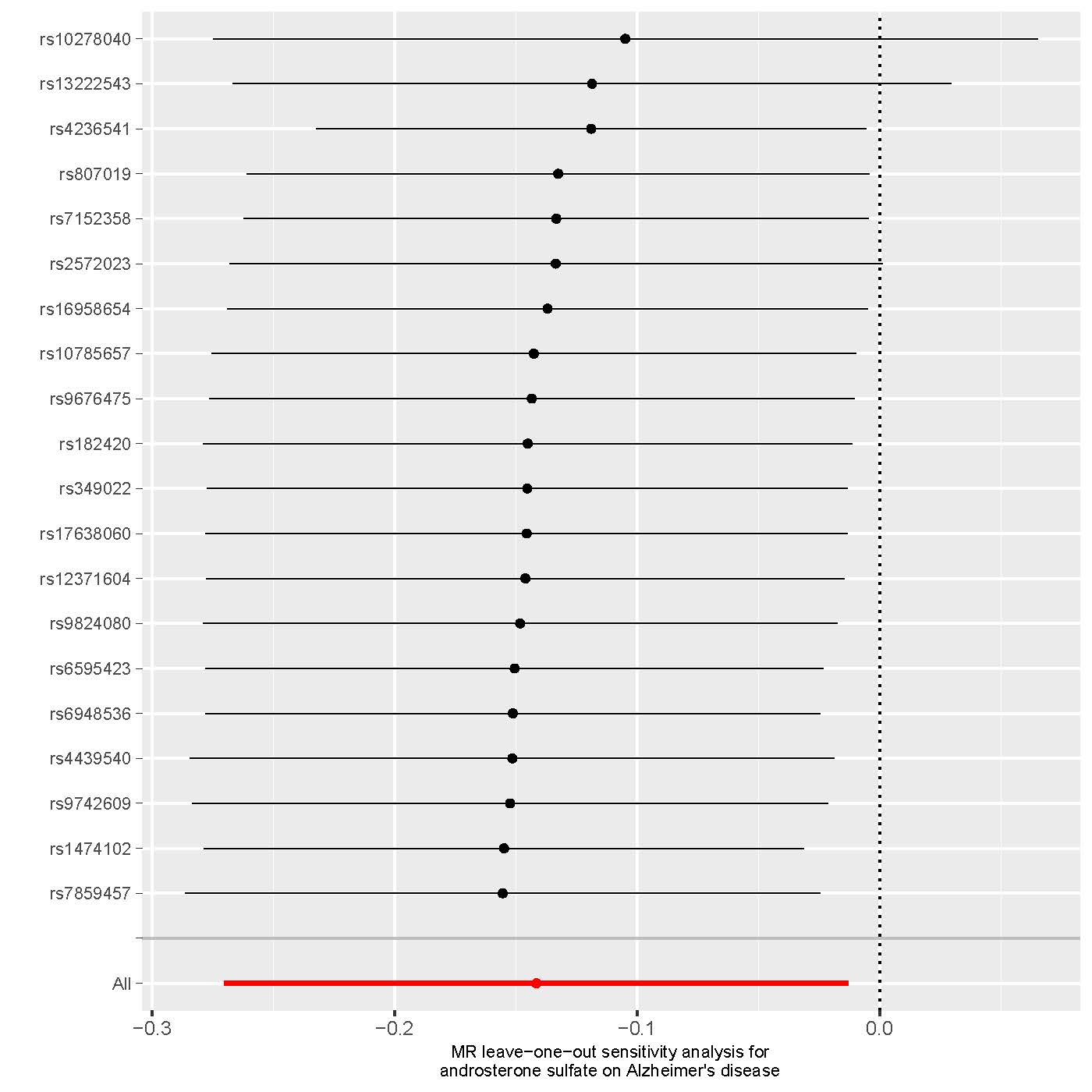

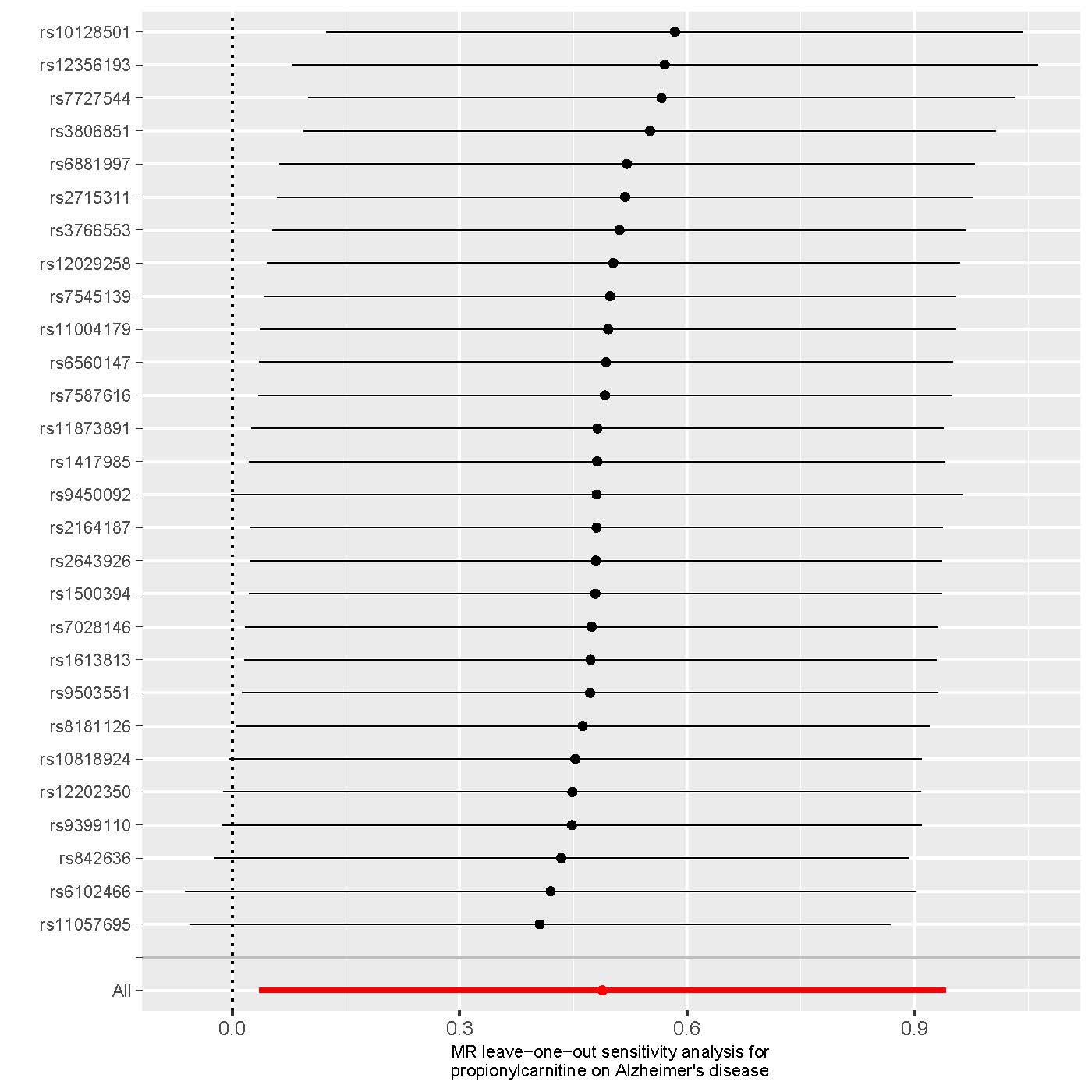
**

**
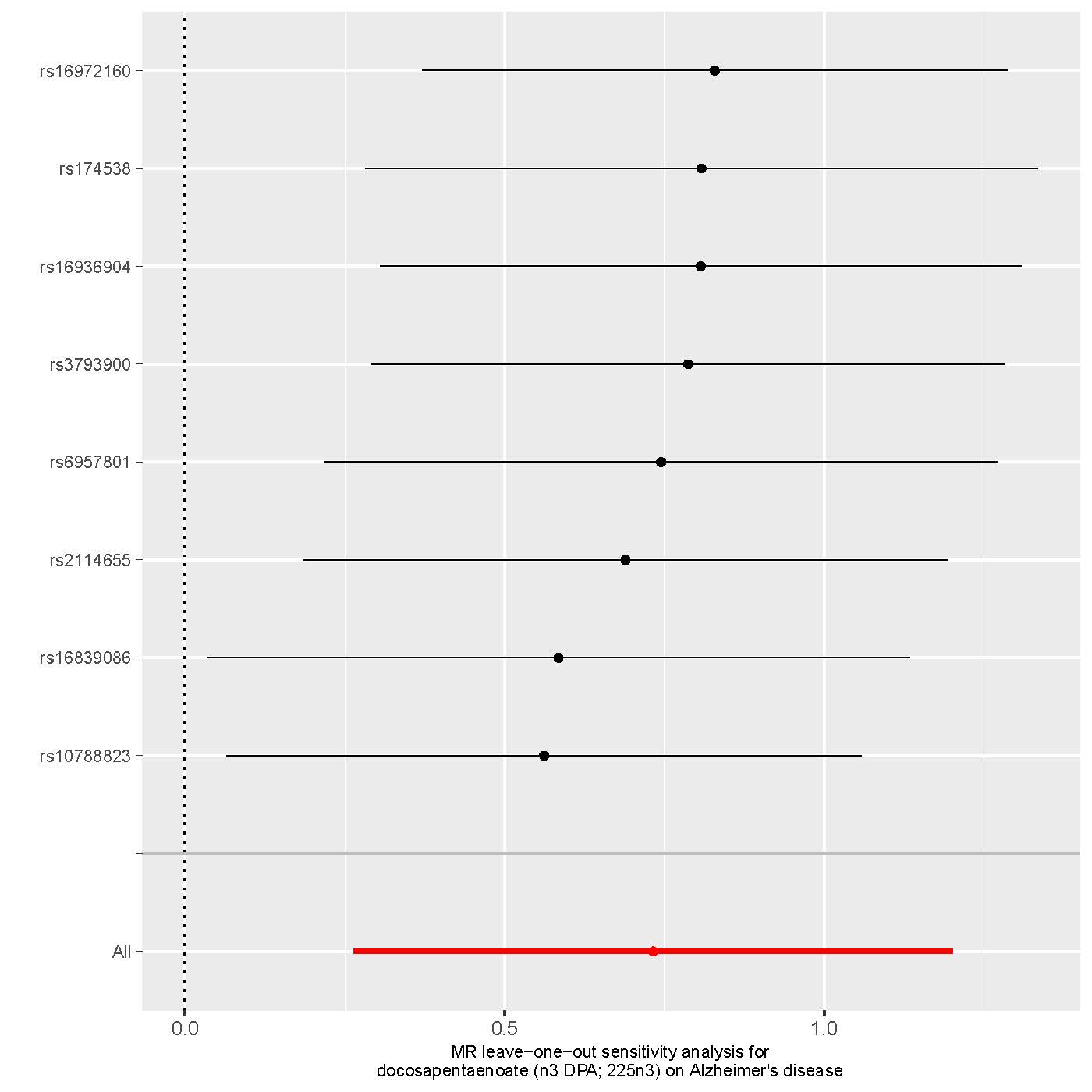

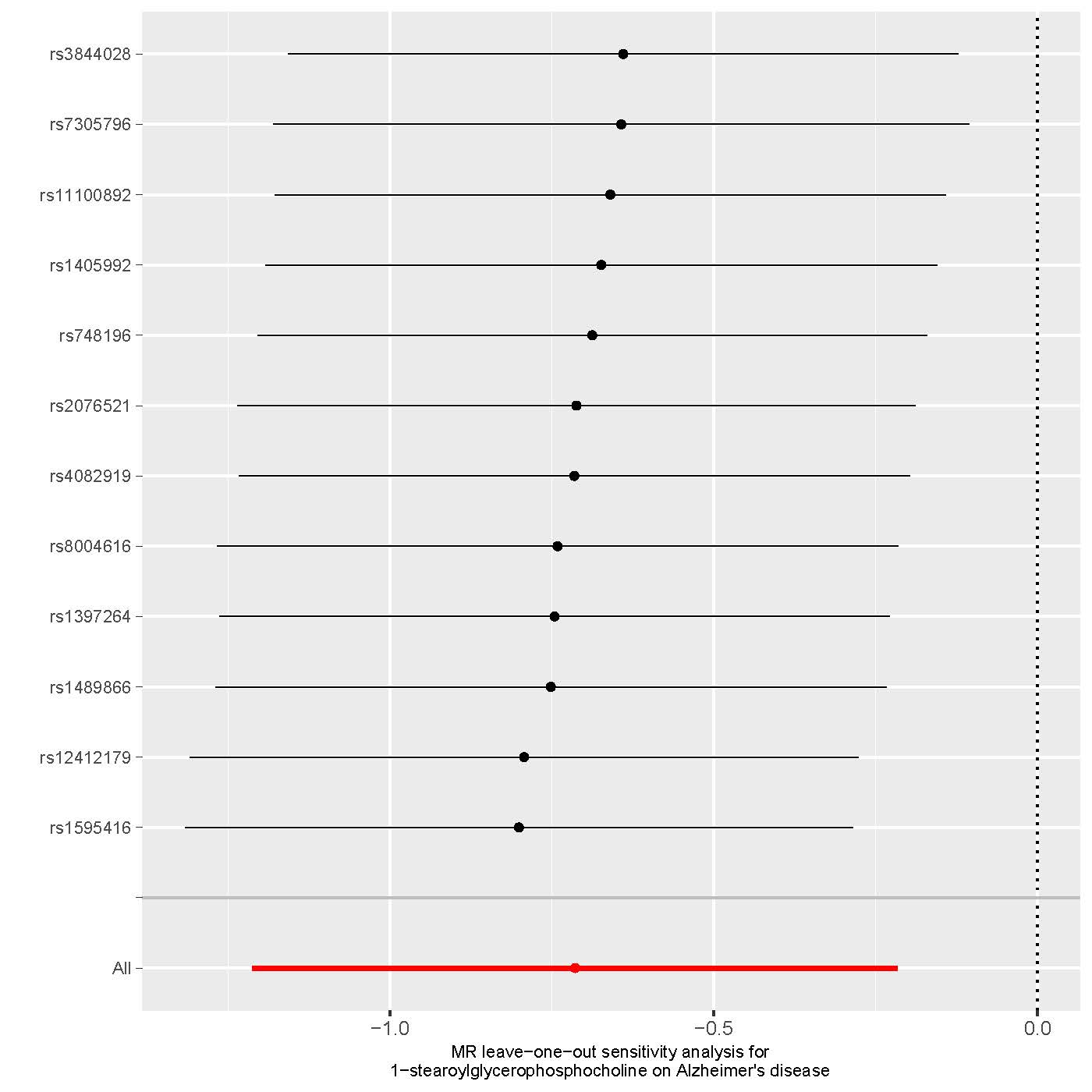
**

**
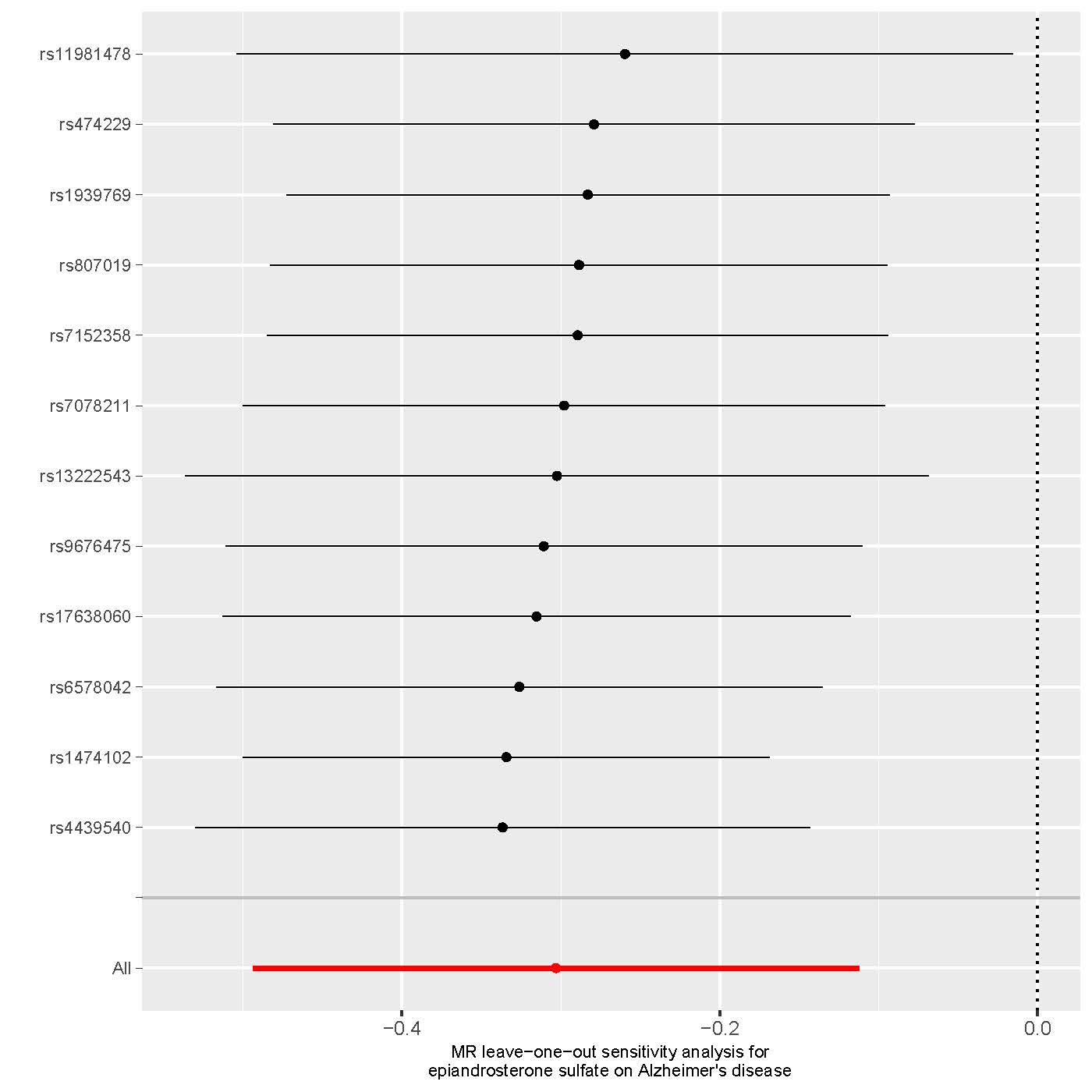

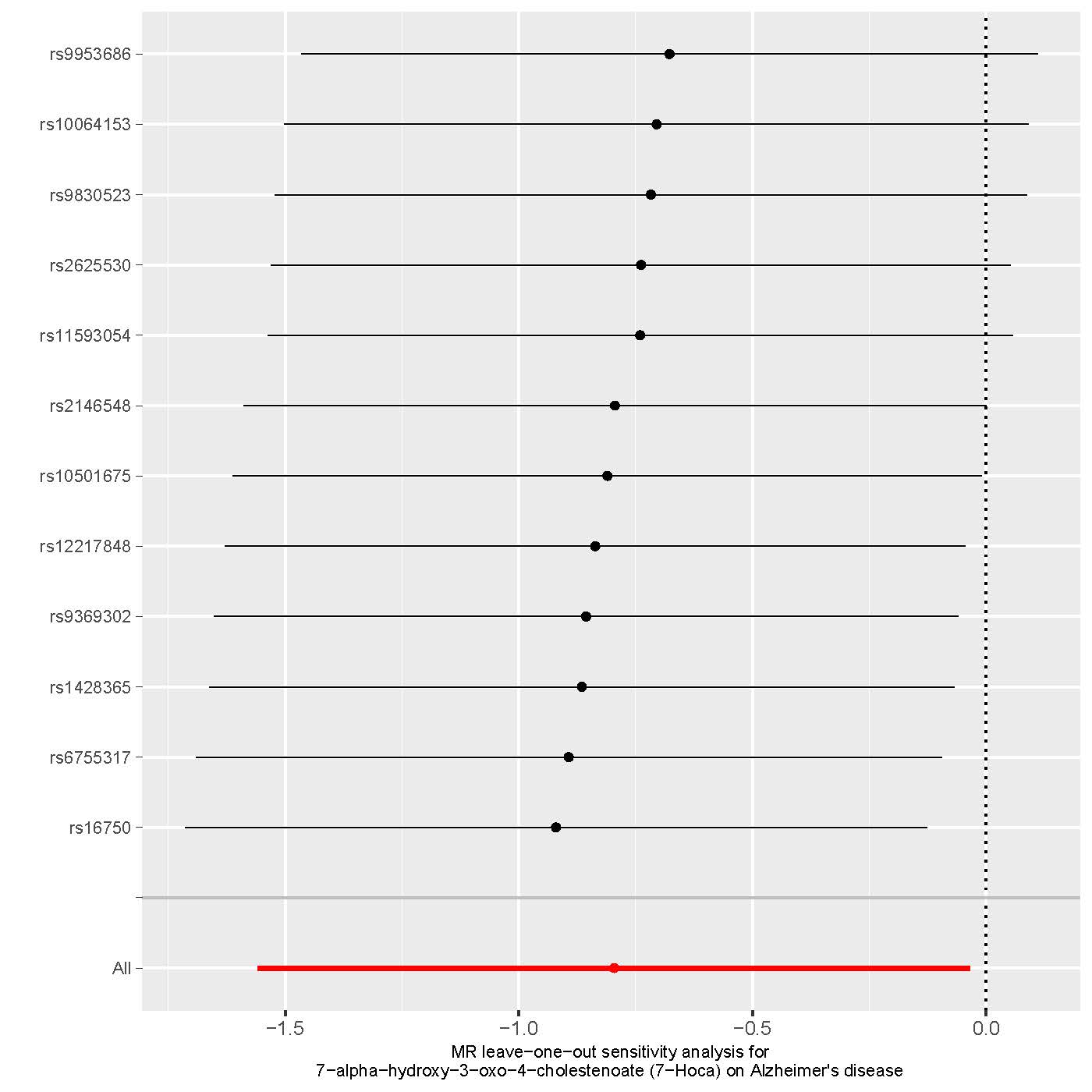
**

**
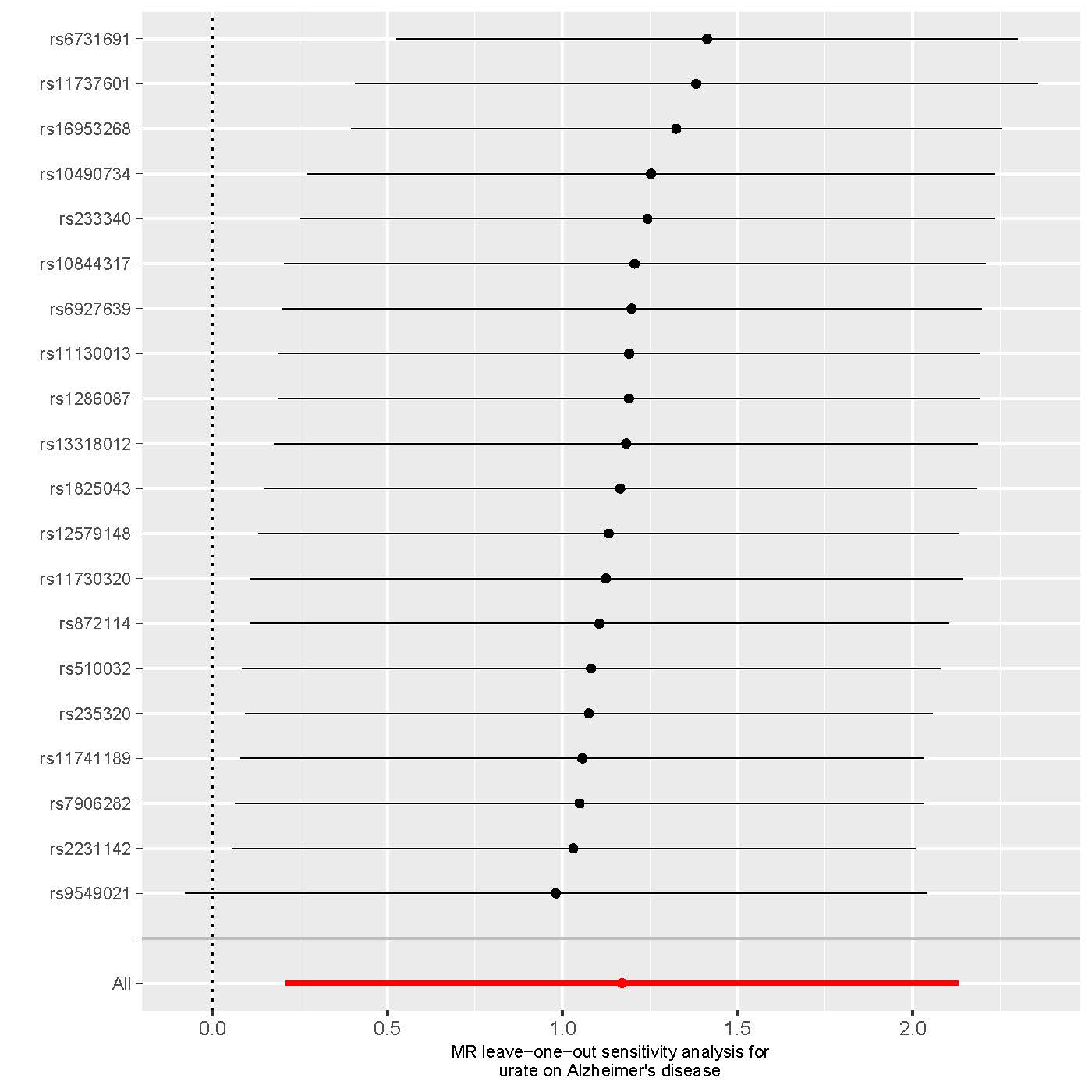

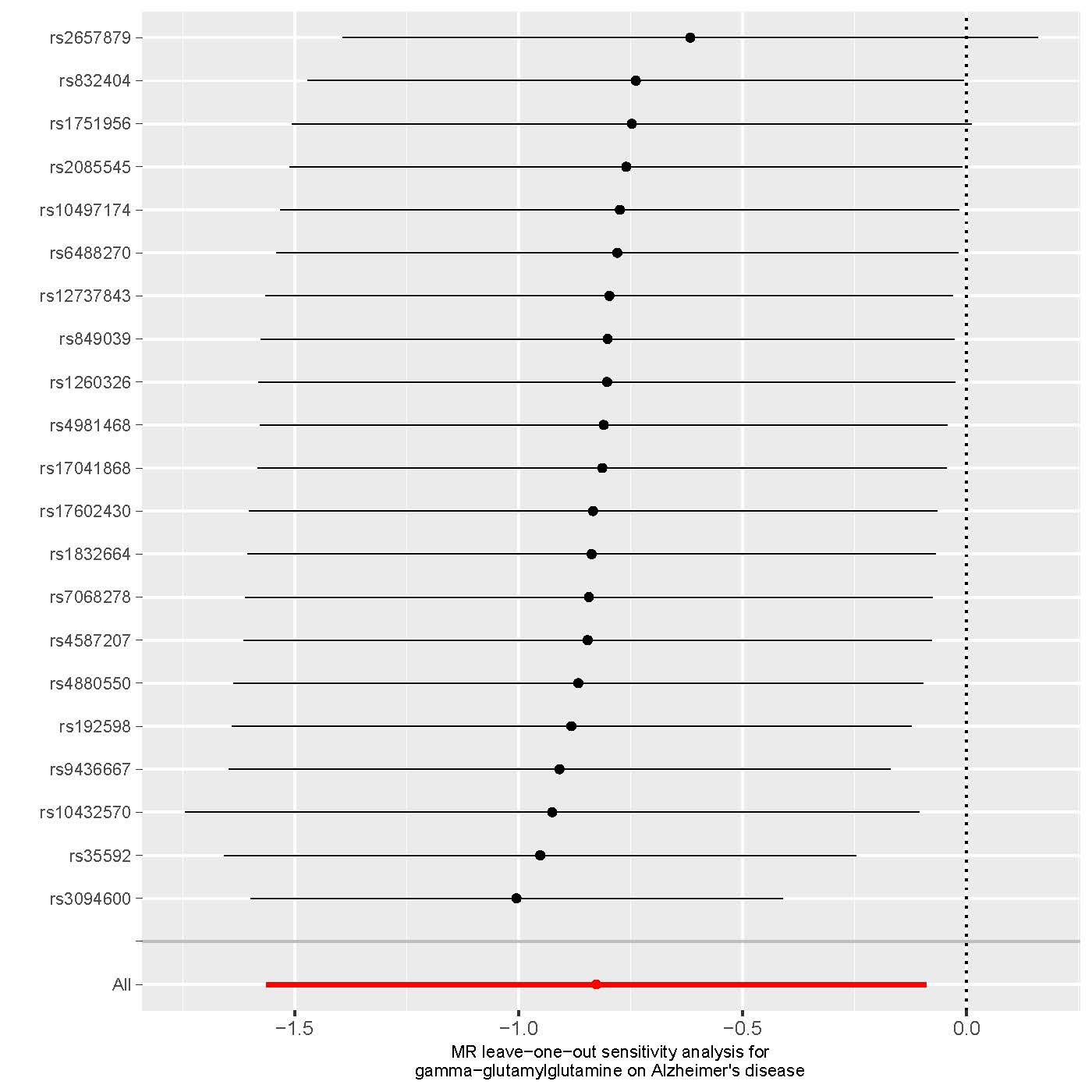
**

**
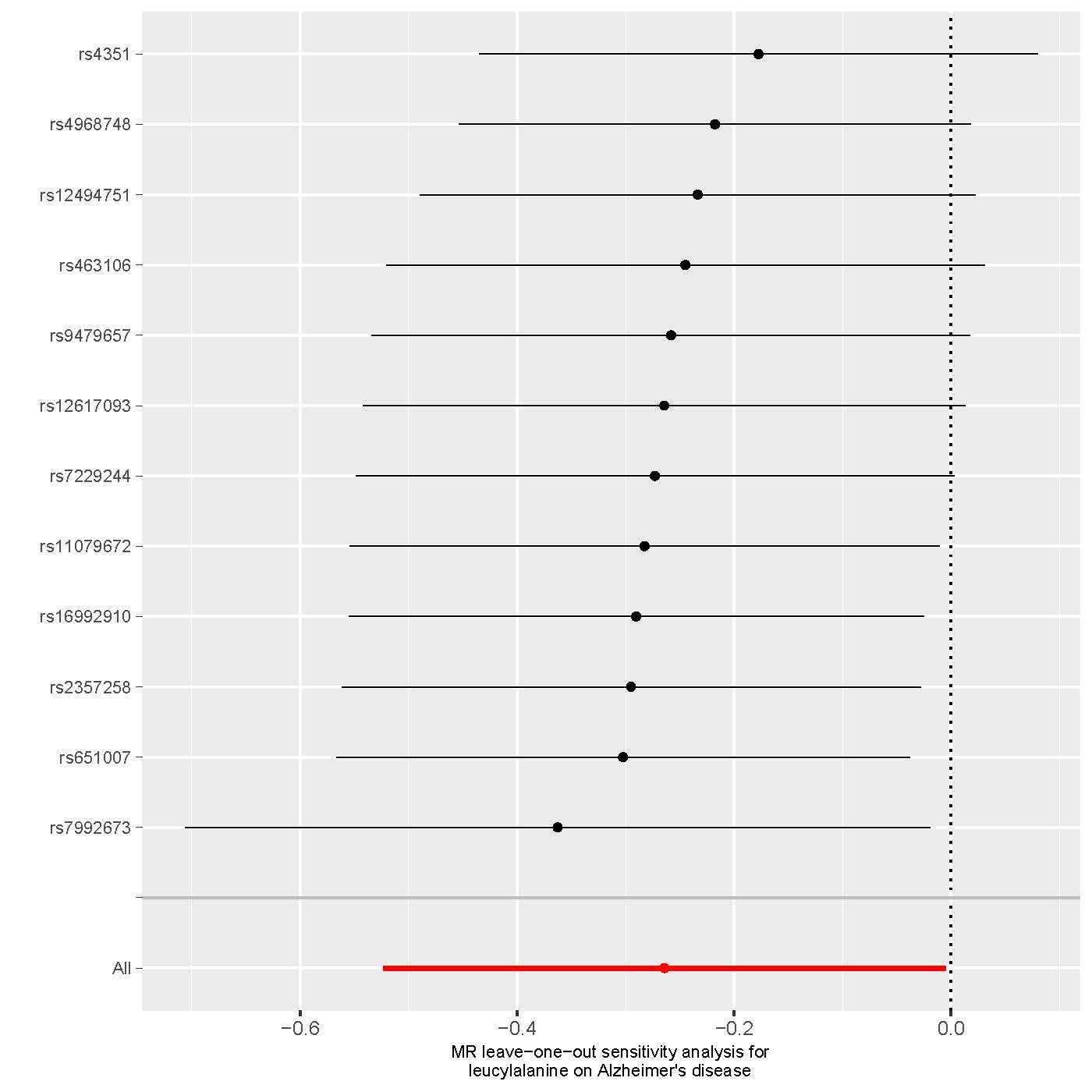

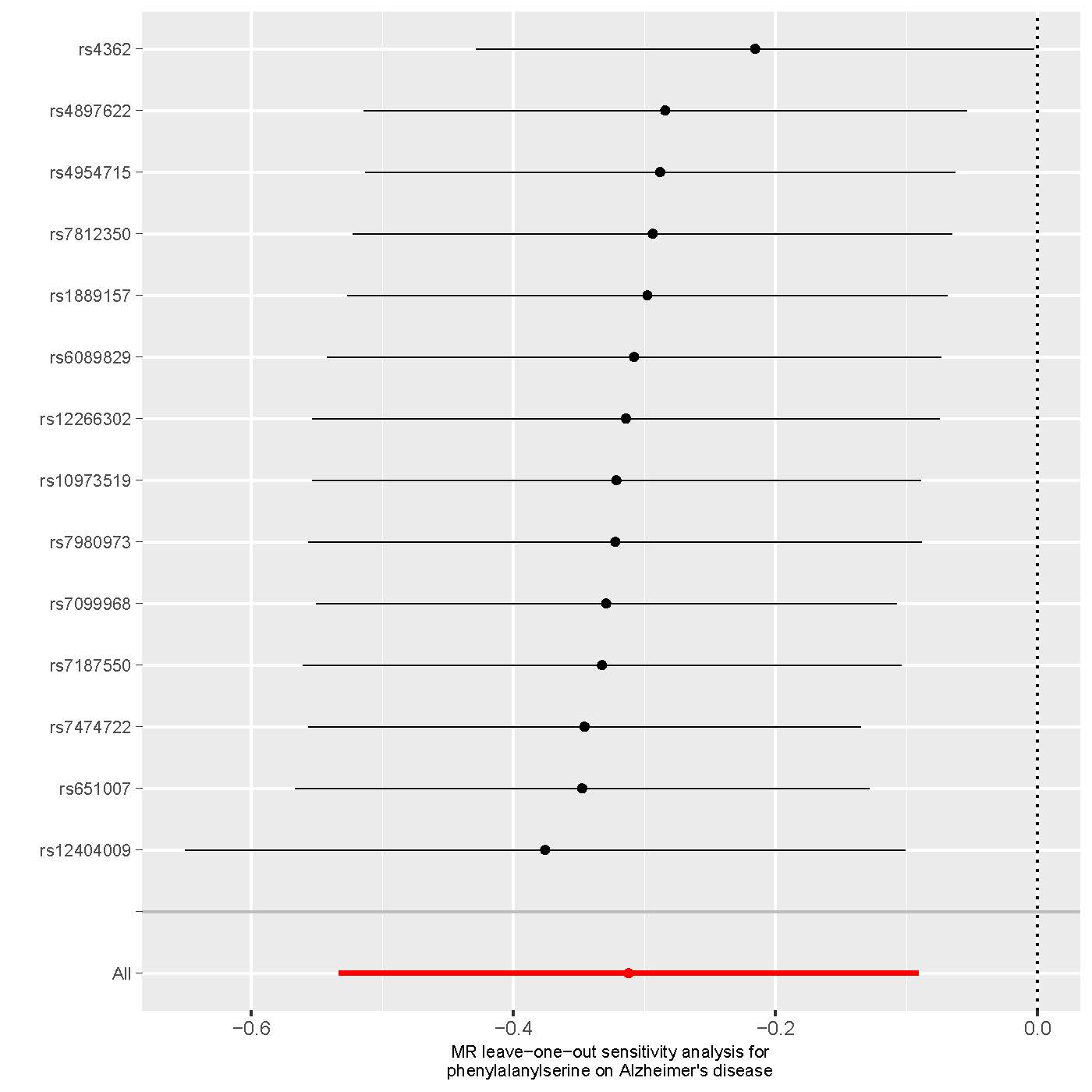
**

**
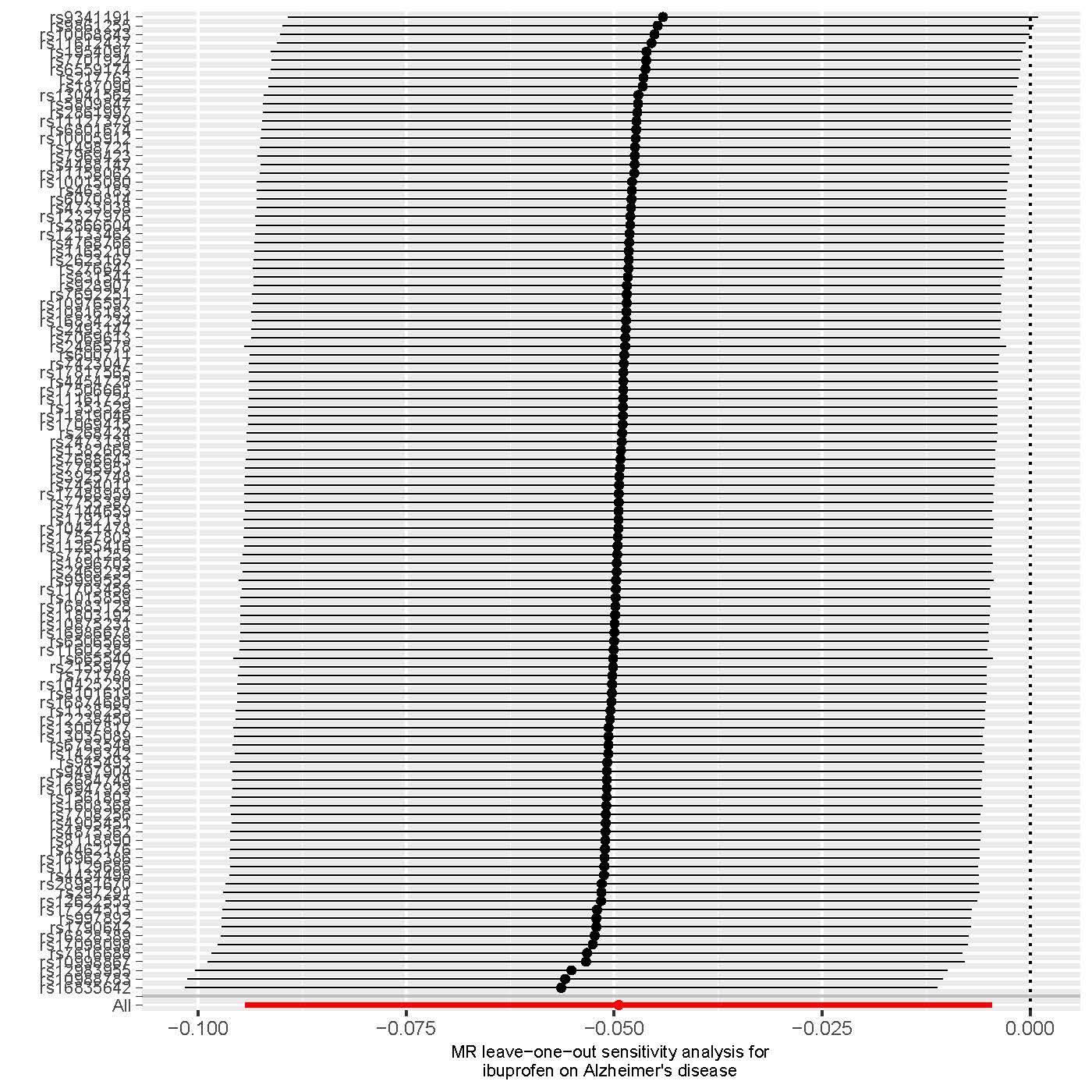

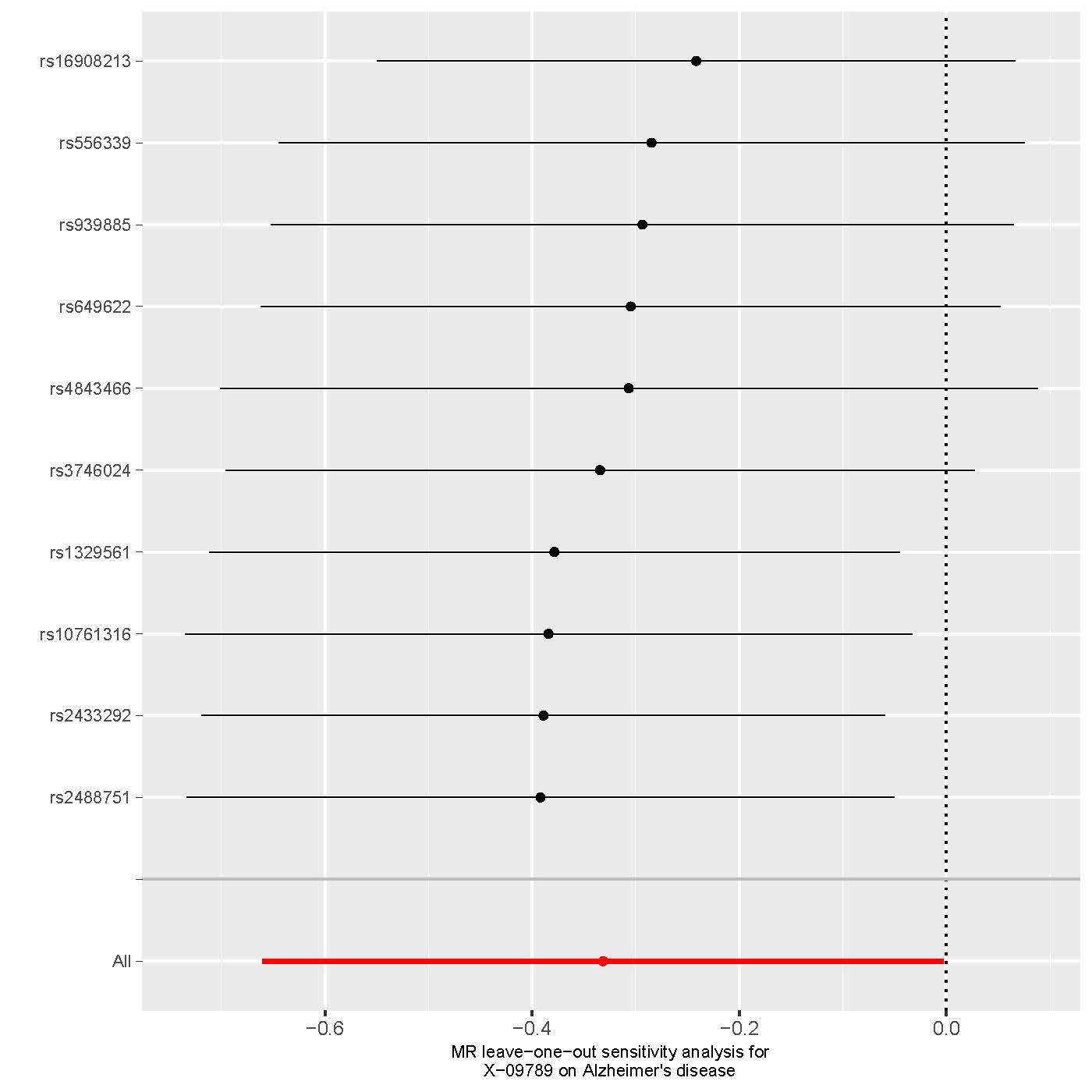
**

**
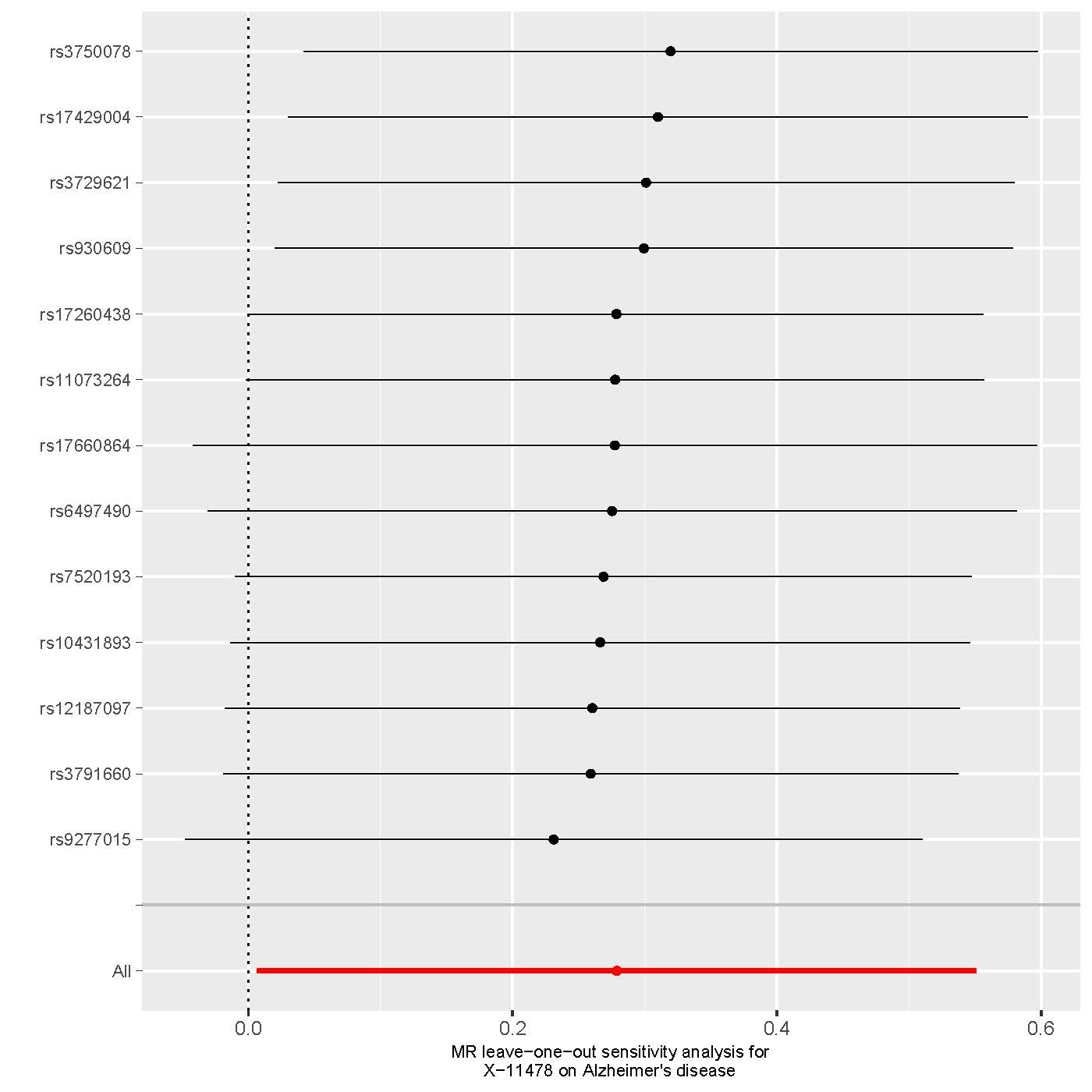

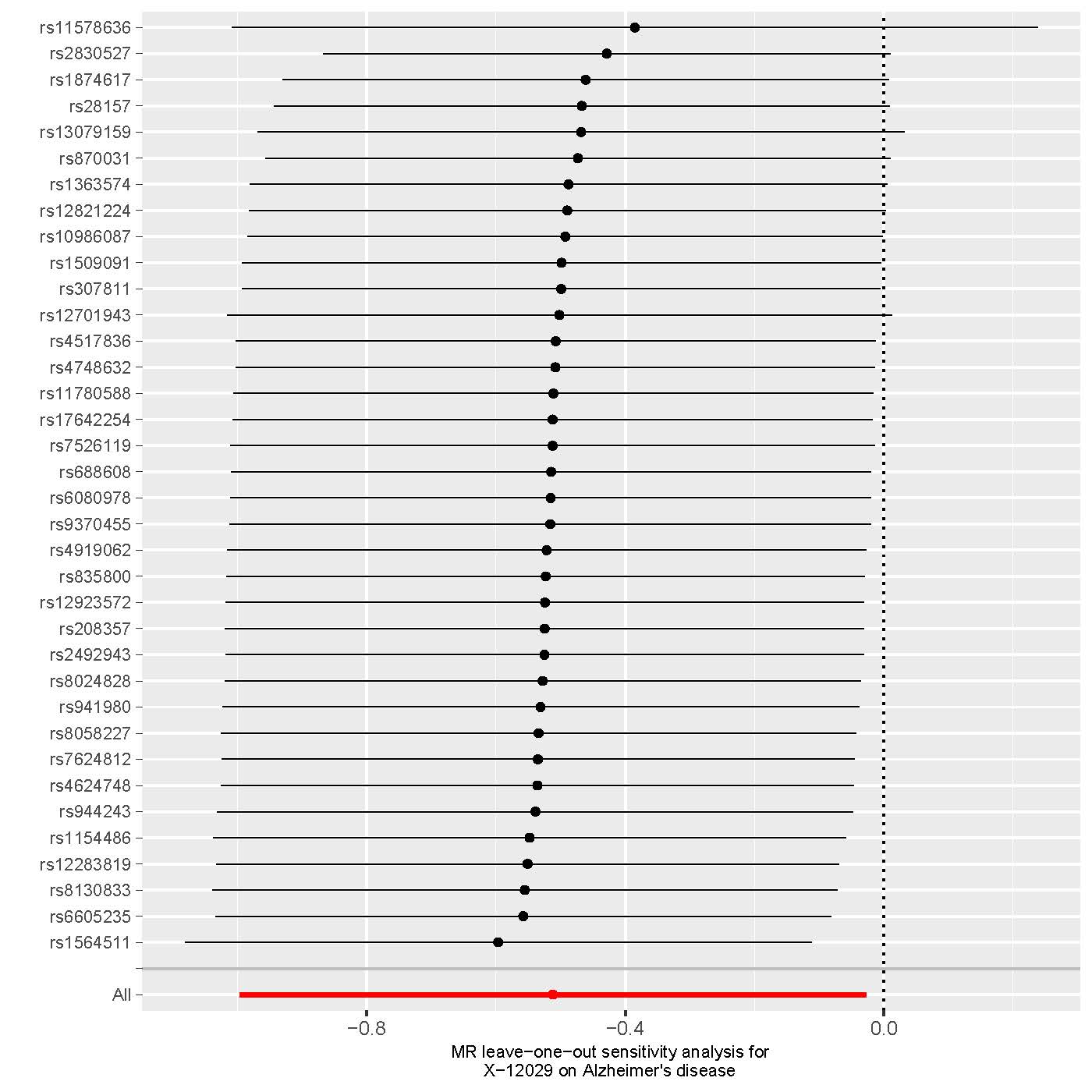
**

**
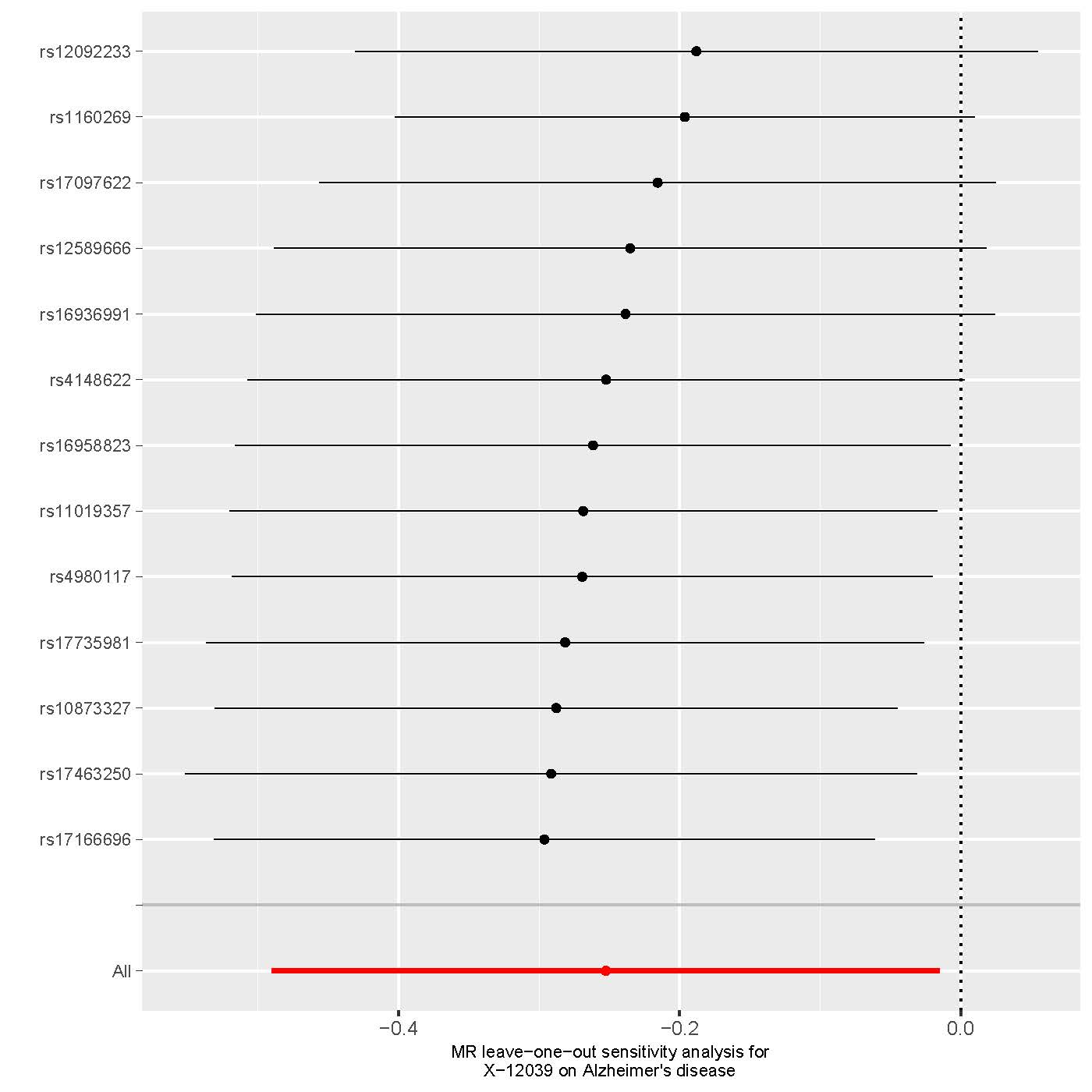

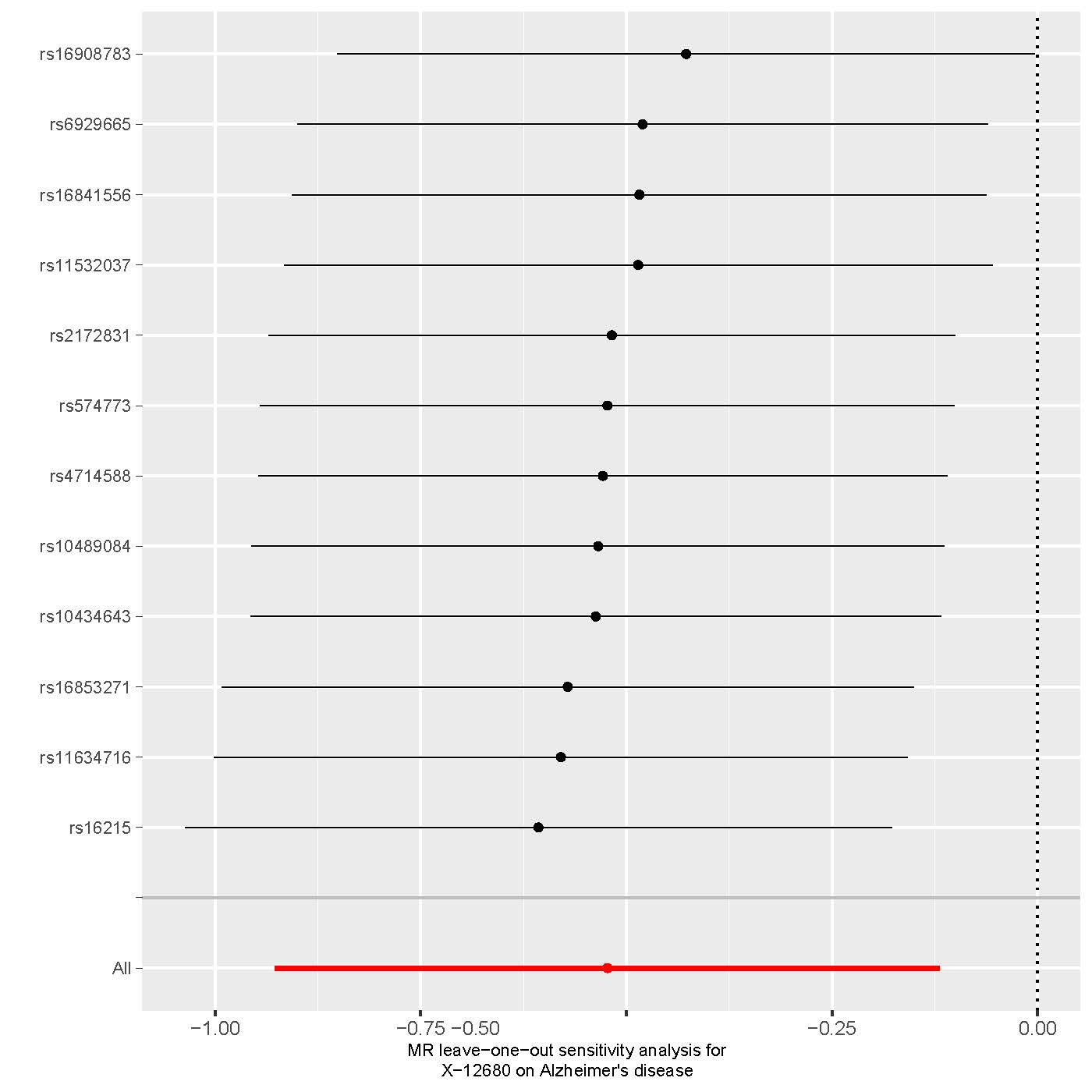
**

**
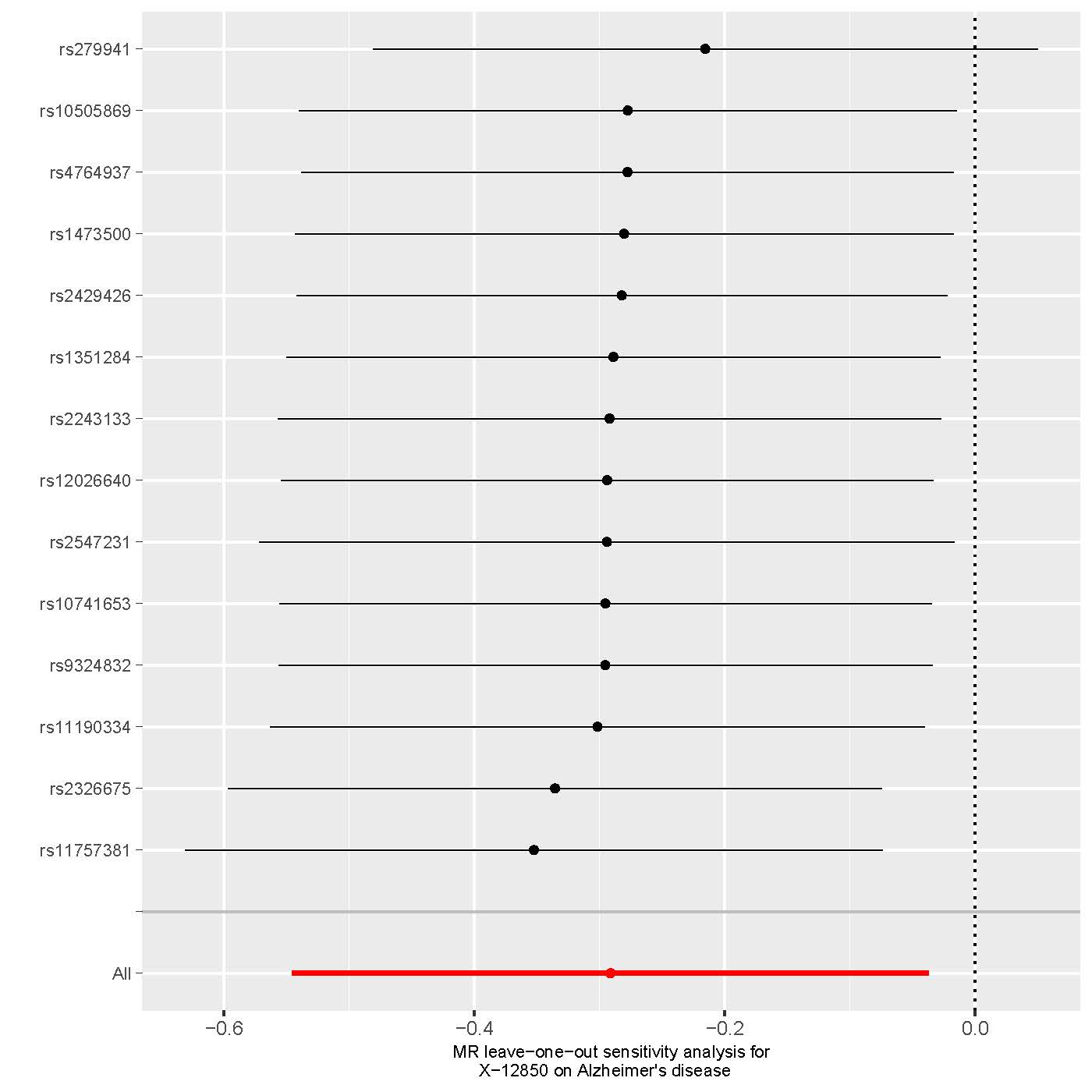
**

**Supplementary Figure S2.** Funnel plots for the suggestive significant IVW estimates between metabolites and Alzheimer's disease.


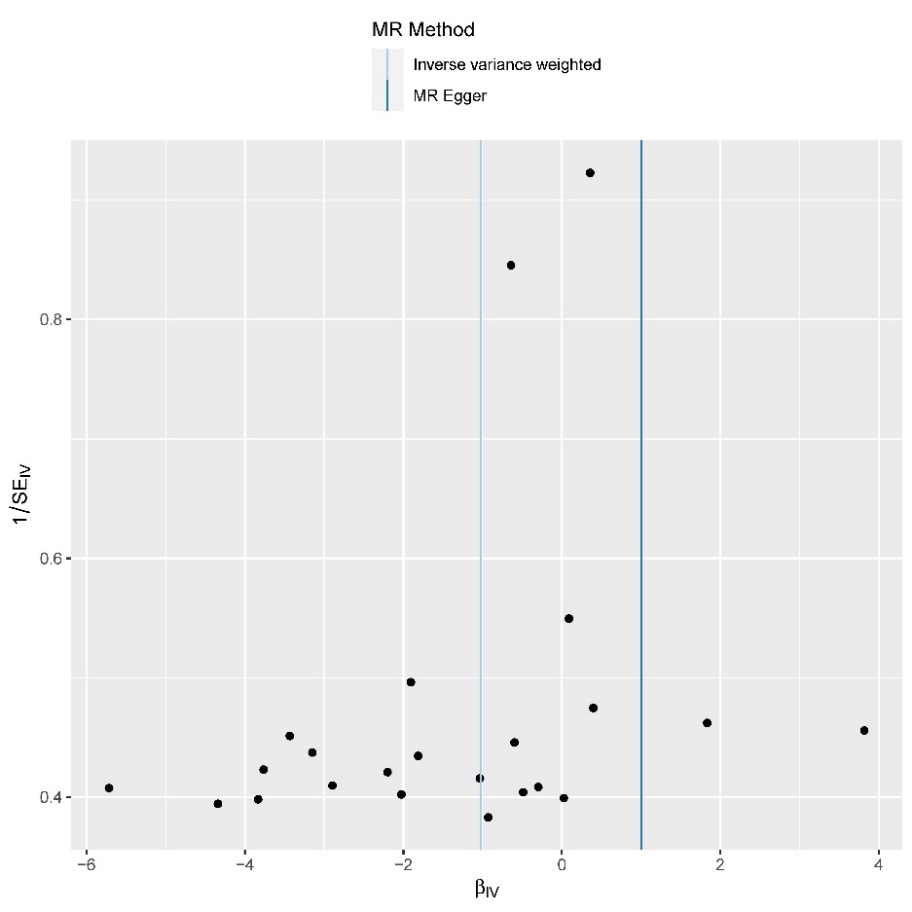


Funnel plot of genetic association estimates for lysine on Alzheimer's disease


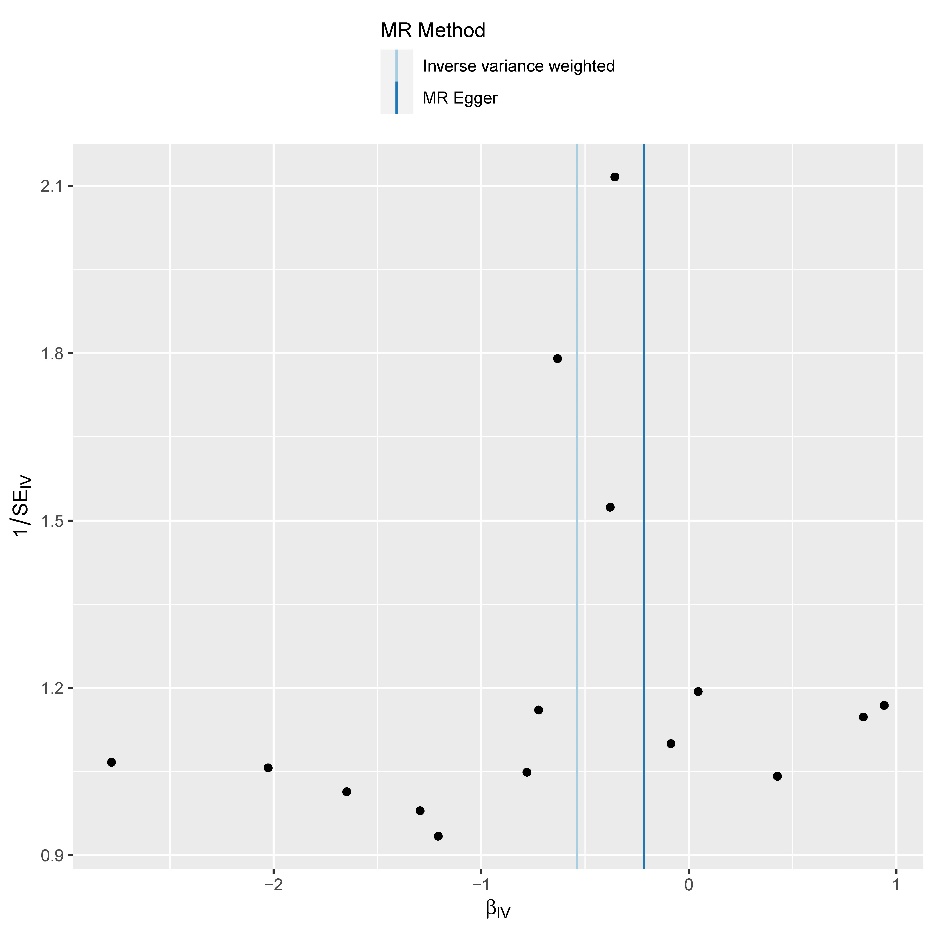


Funnel plot of genetic association estimates for serotonin (5HT) on Alzheimer's disease


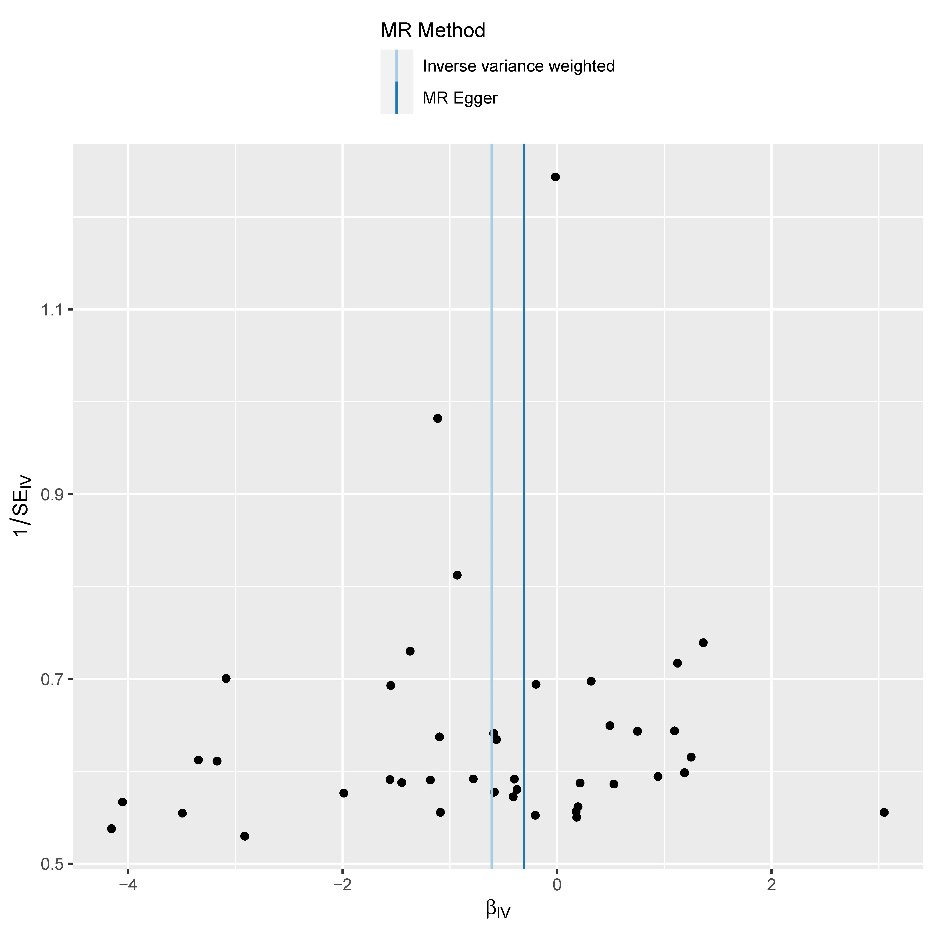


Funnel plot of genetic association estimates for 2−aminobutyrate on Alzheimer's disease
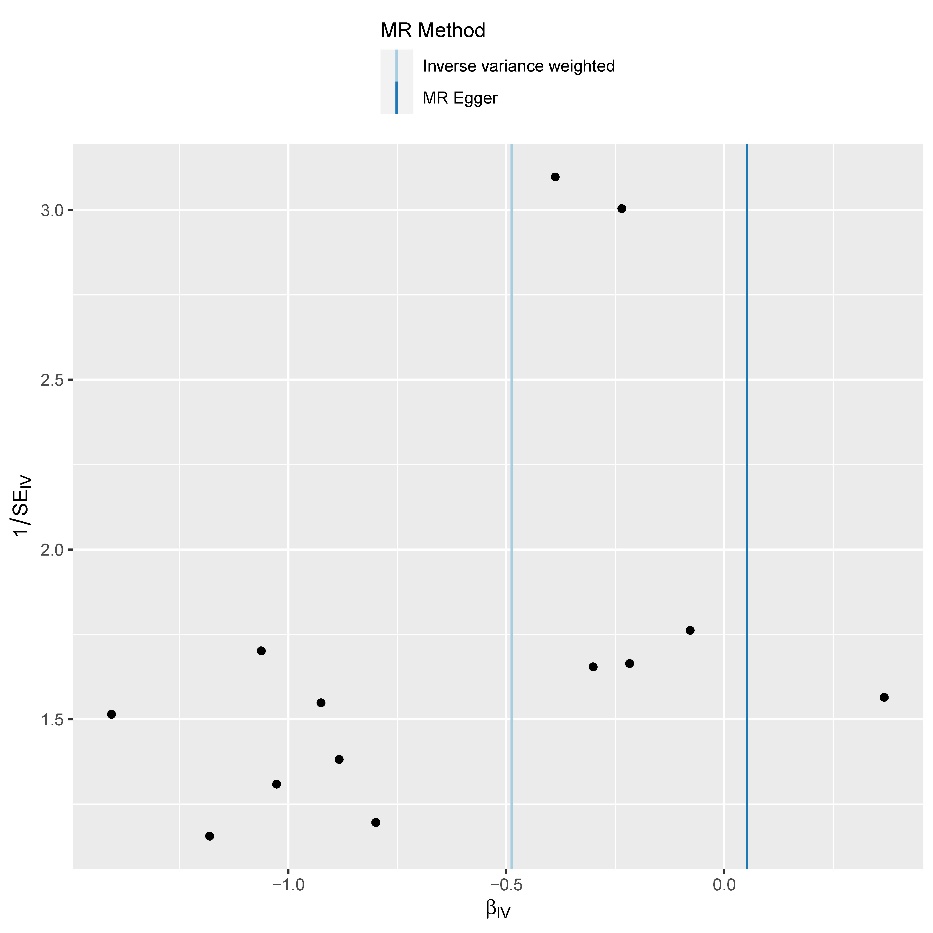


Funnel plot of genetic association estimates for phenol sulfate on Alzheimer's disease
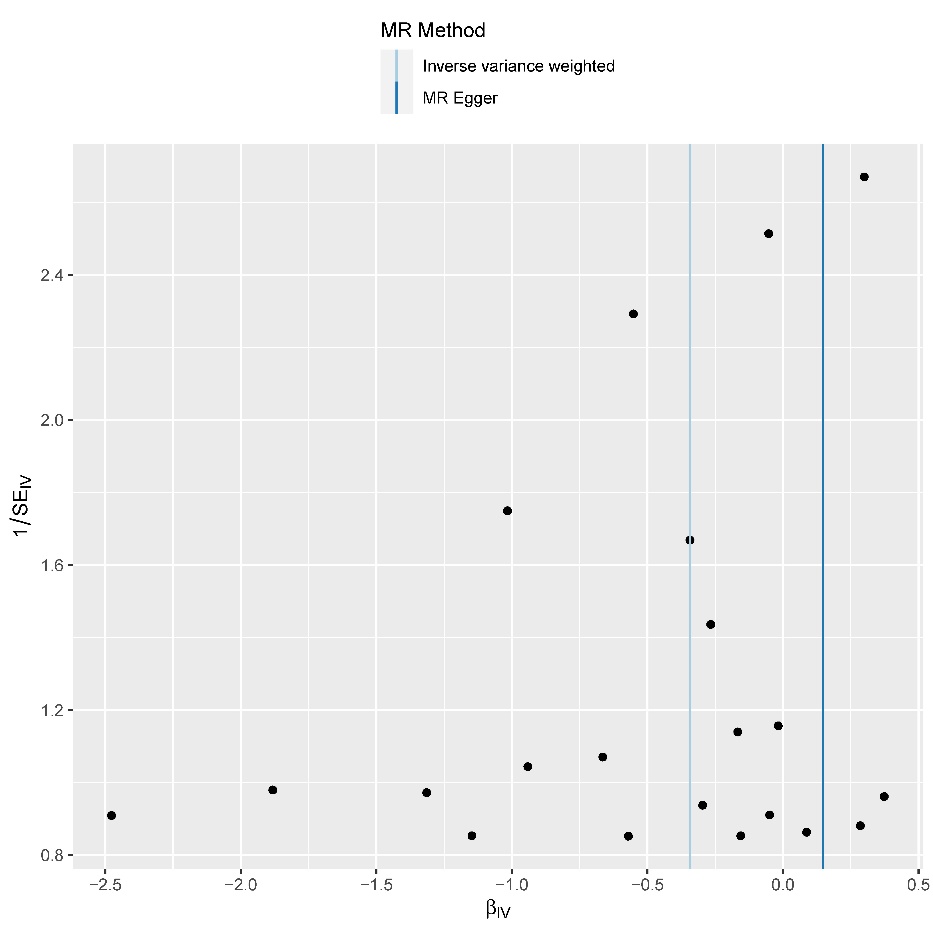


Funnel plot of genetic association estimates for isovalerylcarnitine on Alzheimer's disease
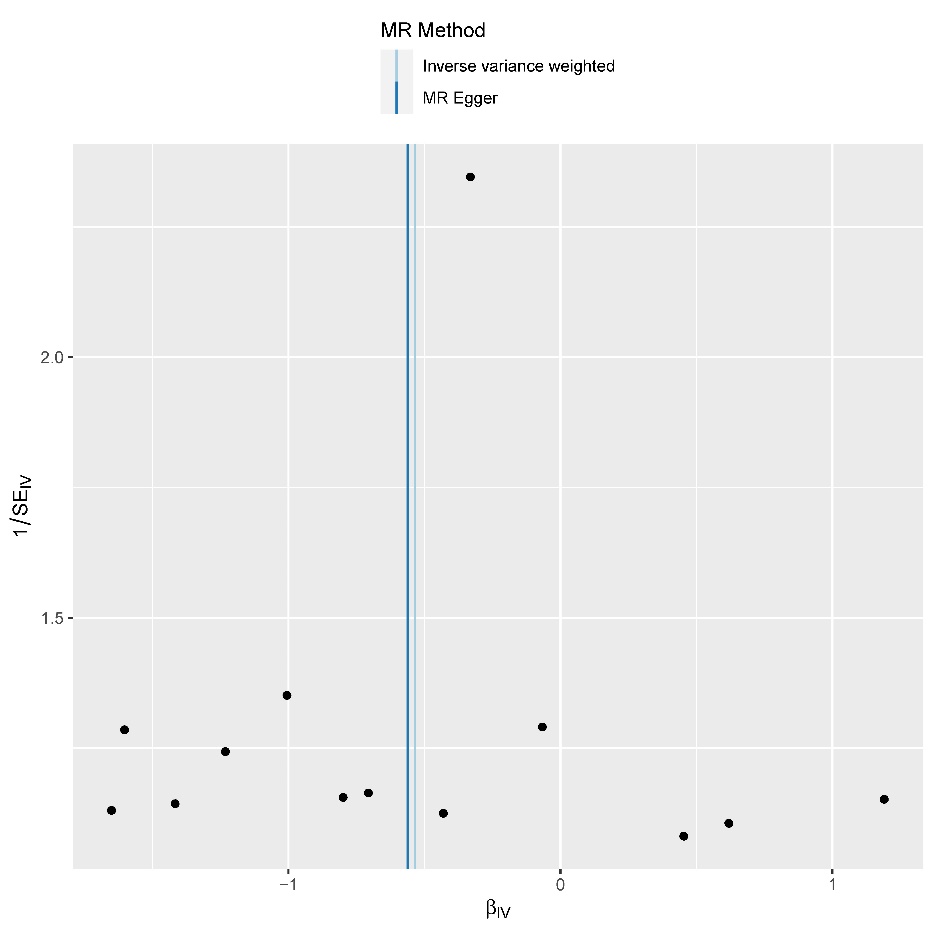


Funnel plot of genetic association estimates for pyruvate on Alzheimer's disease
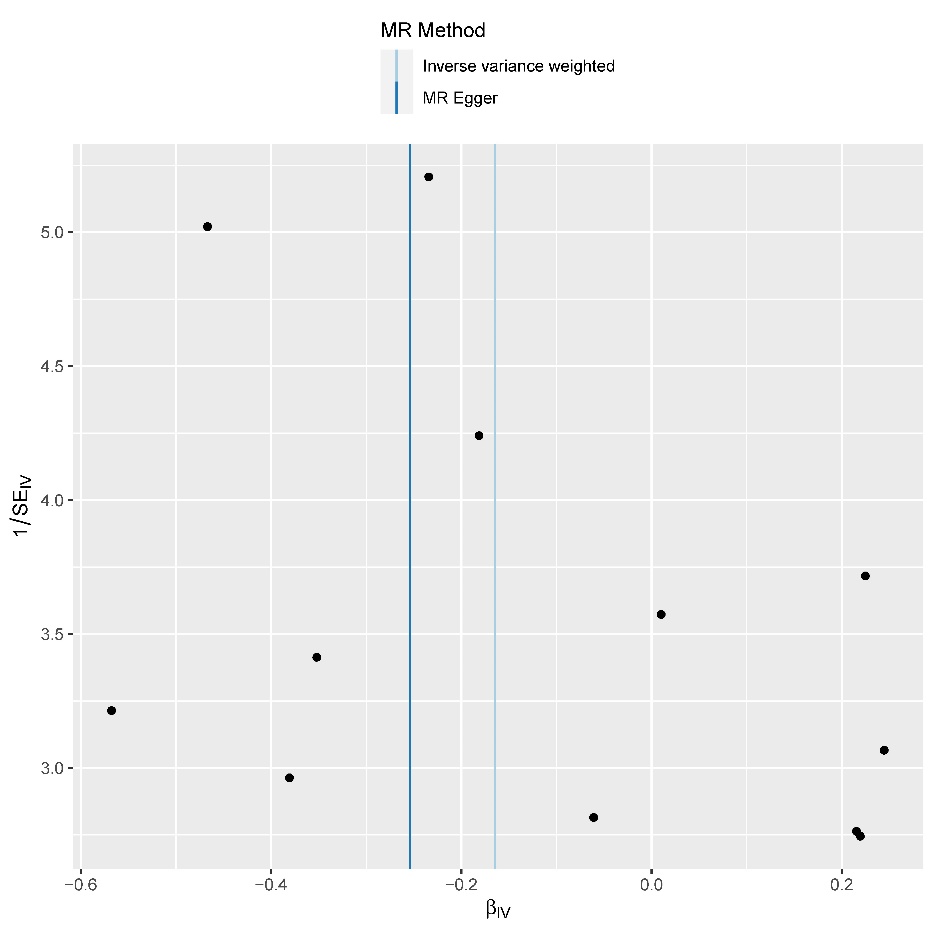


Funnel plot of genetic association estimates for mannitol on Alzheimer's disease
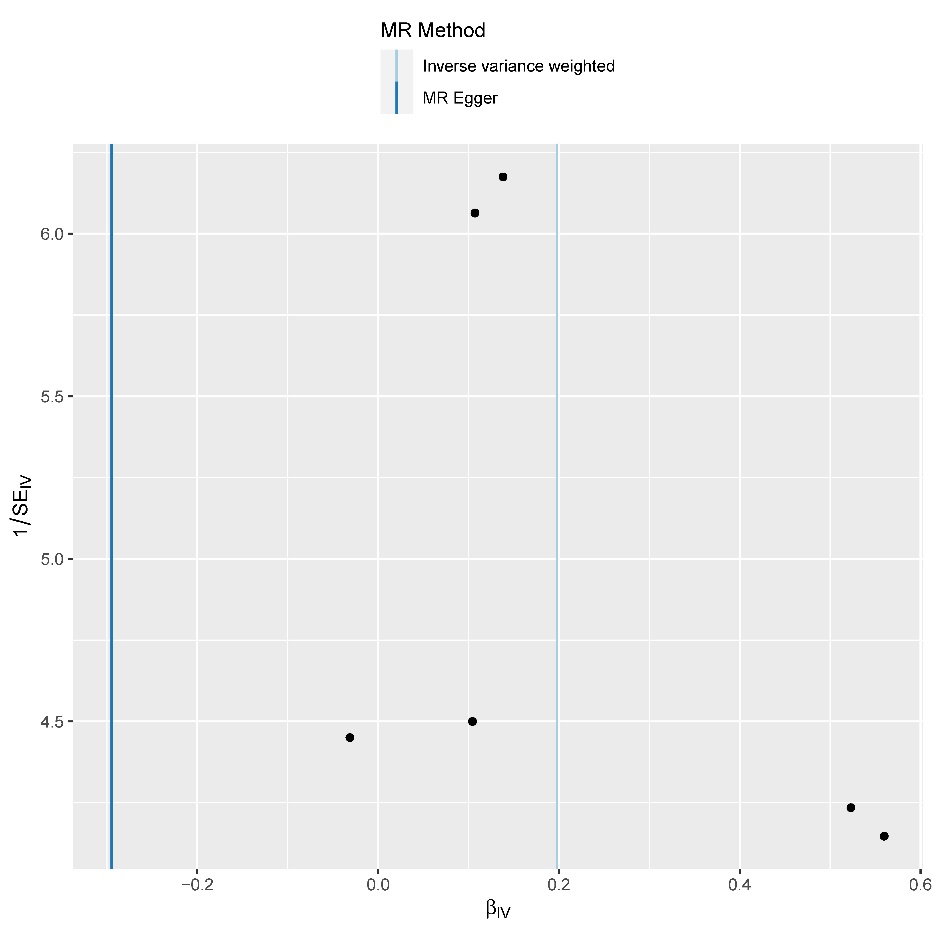


Funnel plot of genetic association estimates for glycodeoxycholate on Alzheimer's disease
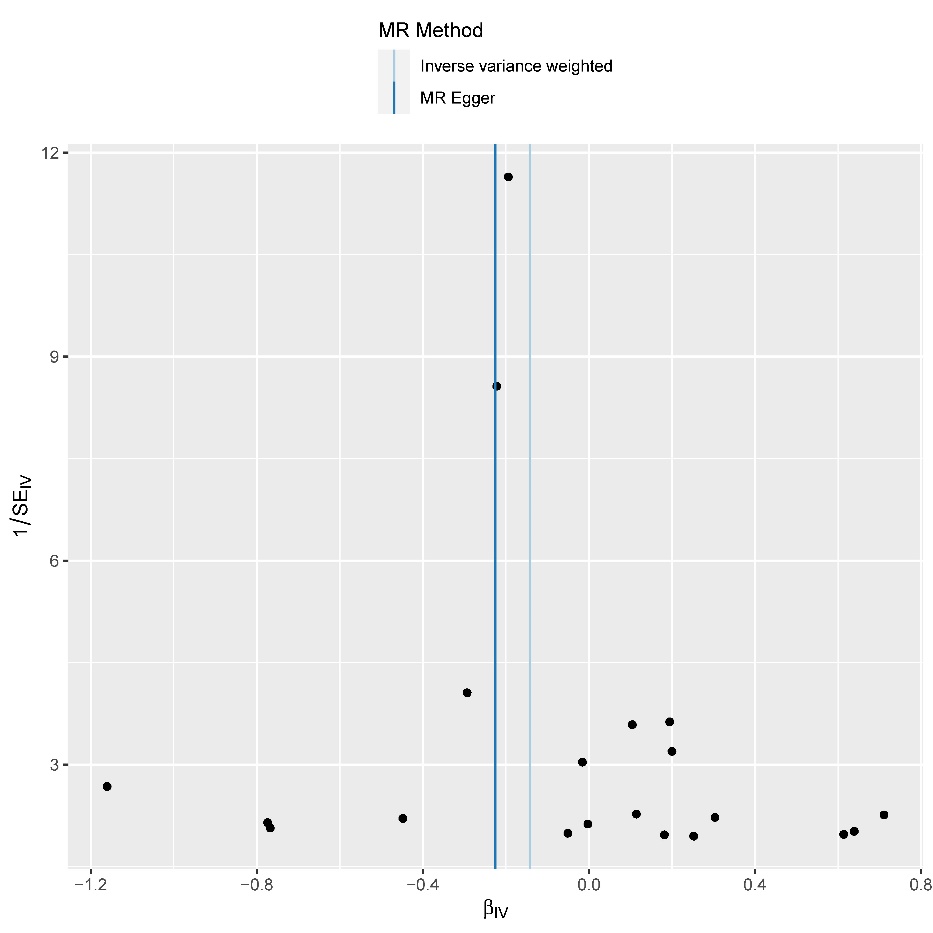


Funnel plot of genetic association estimates for androsterone sulfate on Alzheimer's disease
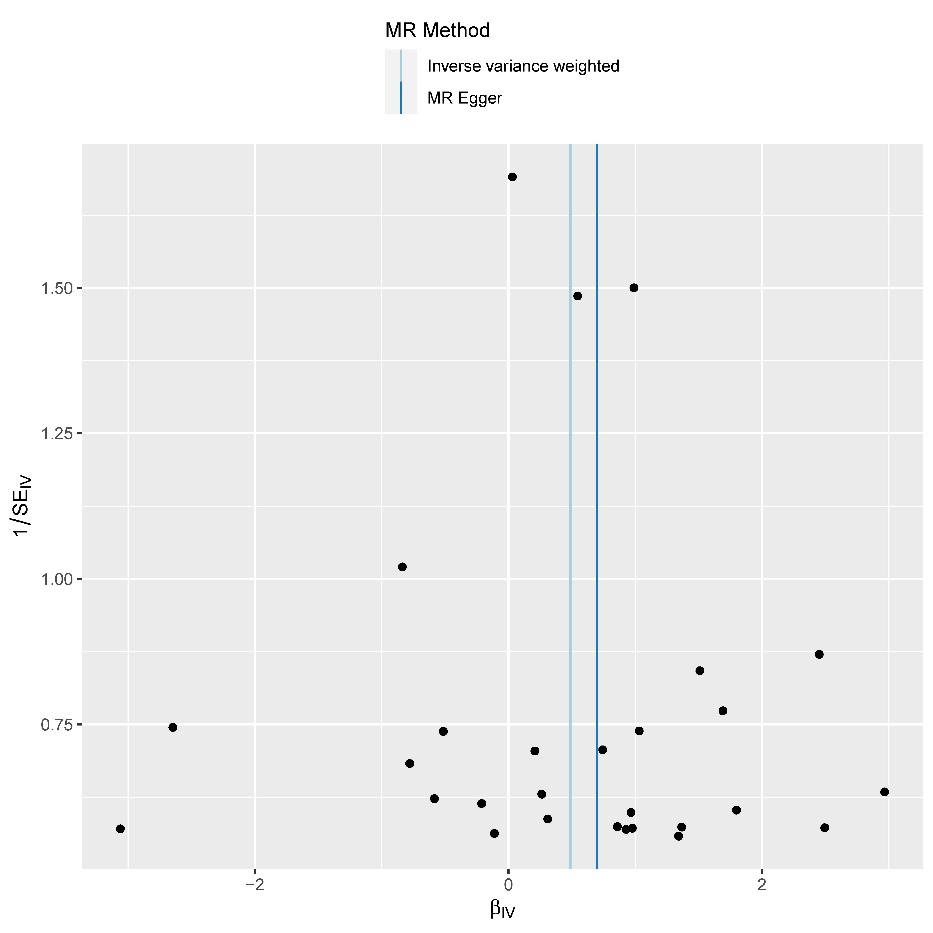


Funnel plot of genetic association estimates for propionylcarnitine on Alzheimer's disease
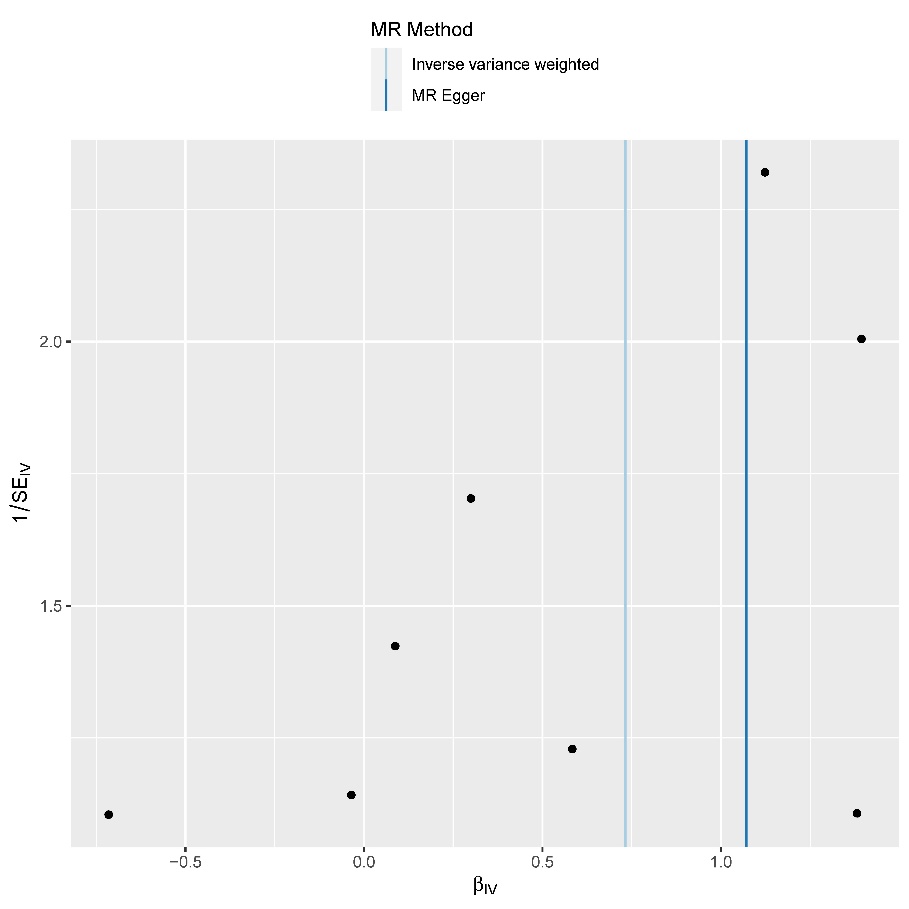


Funnel plot of genetic association estimates for docosapentaenoate (n3 DPA; 22:5n3) on Alzheimer's disease


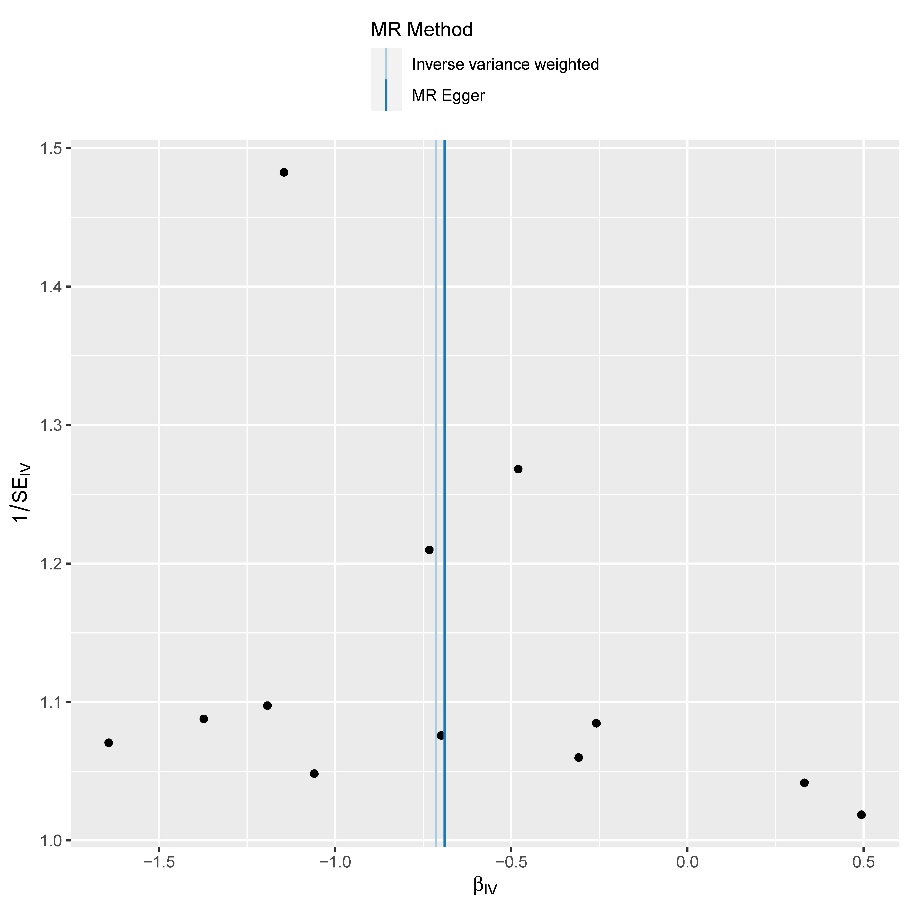


Funnel plot of genetic association estimates for 1−stearoylglycerophosphocholine on Alzheimer's disease


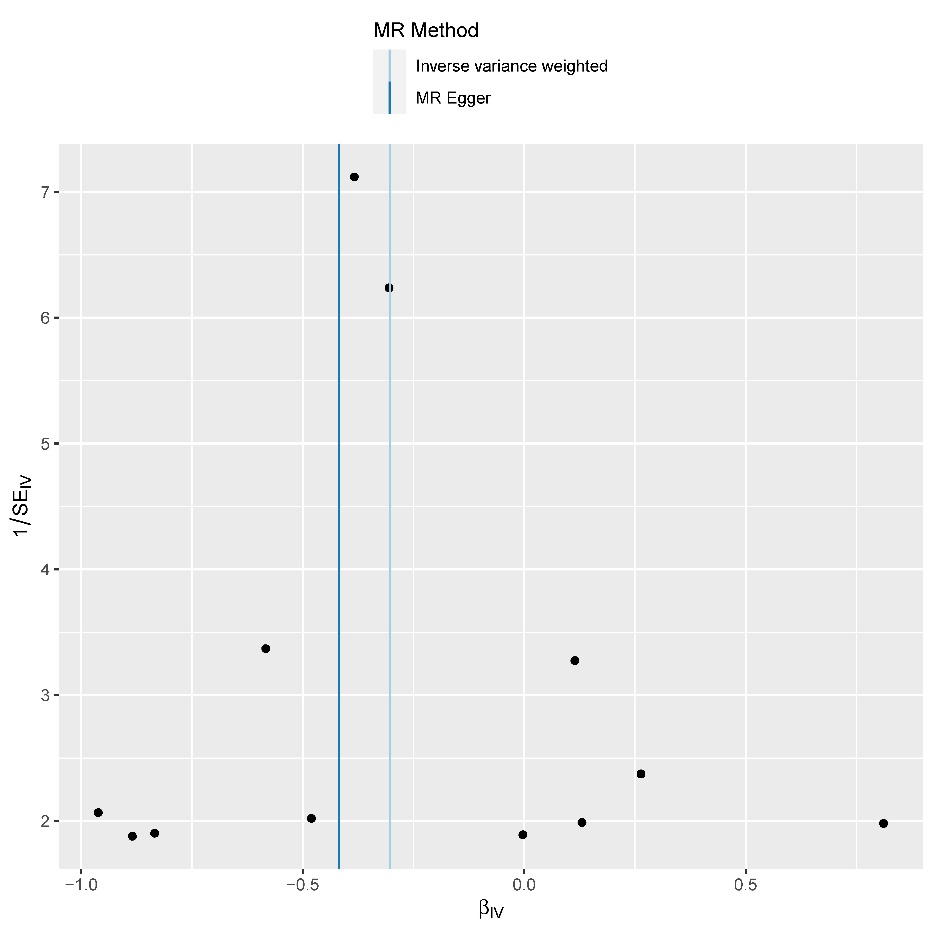


Funnel plot of genetic association estimates for epiandrosterone sulfate on Alzheimer's disease
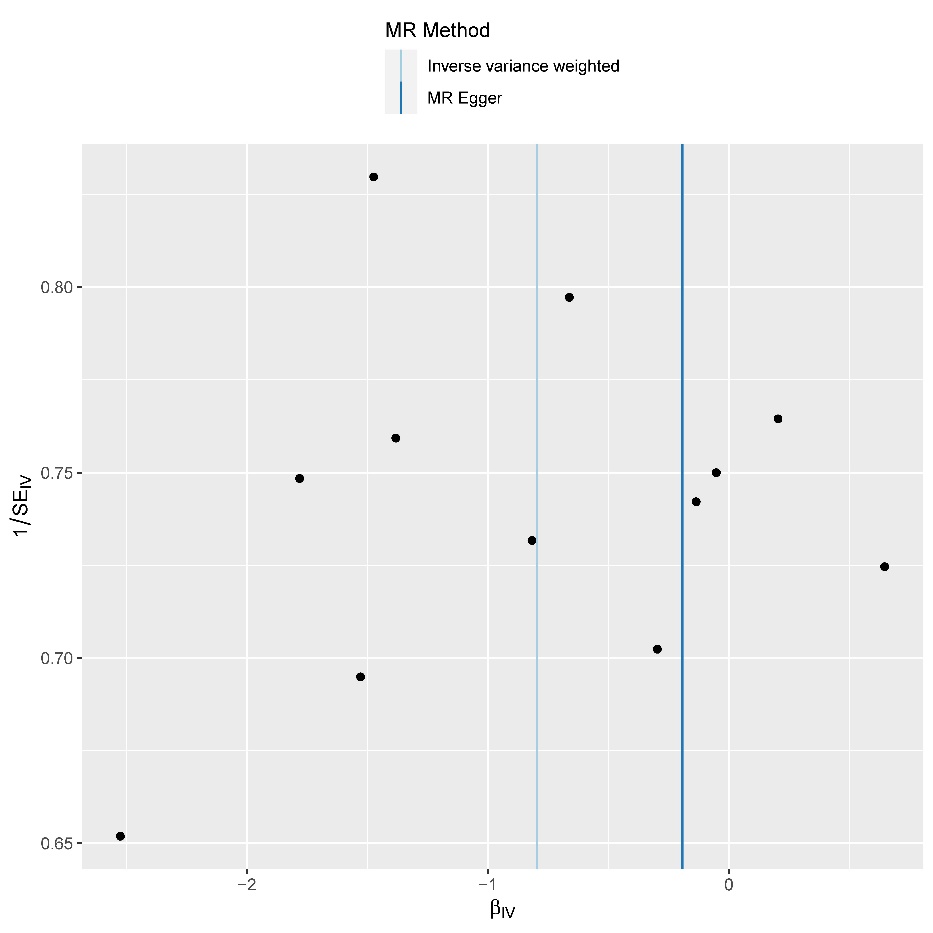


Funnel plot of genetic association estimates for 7−alpha−hydroxy−3−oxo−4−cholestenoate (7-Hoca) on Alzheimer disease
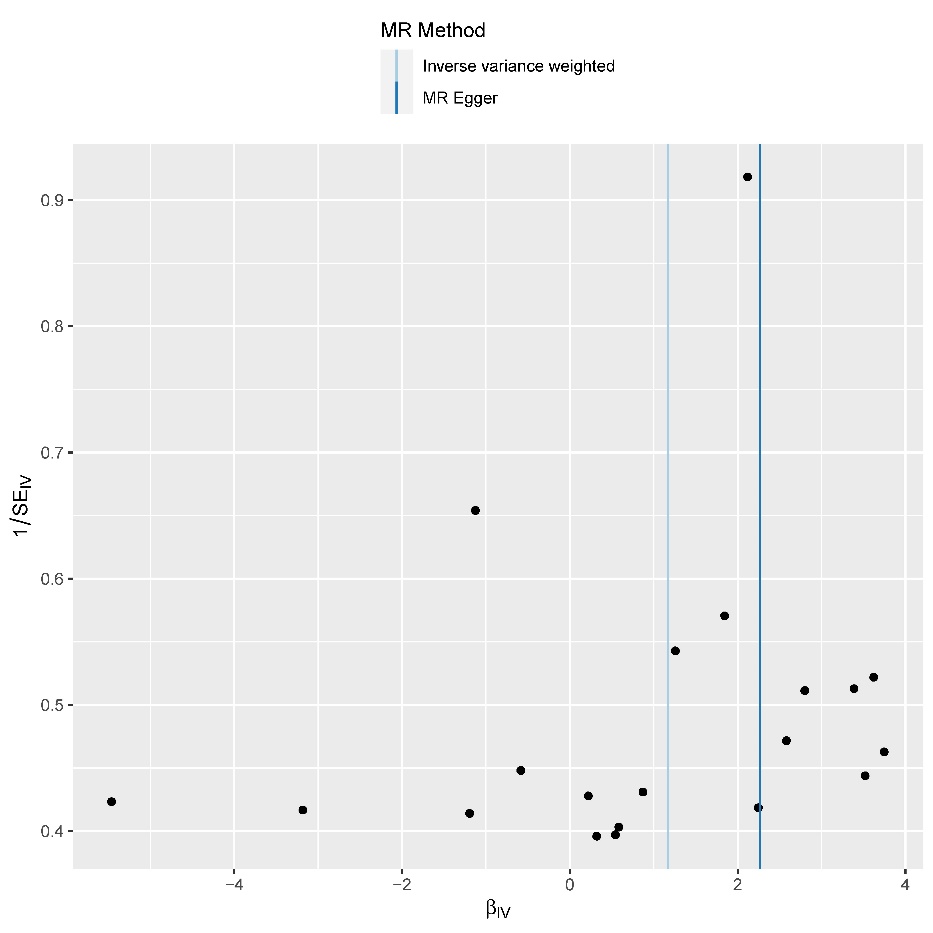


Funnel plot of genetic association estimates for urate on Alzheimer's disease
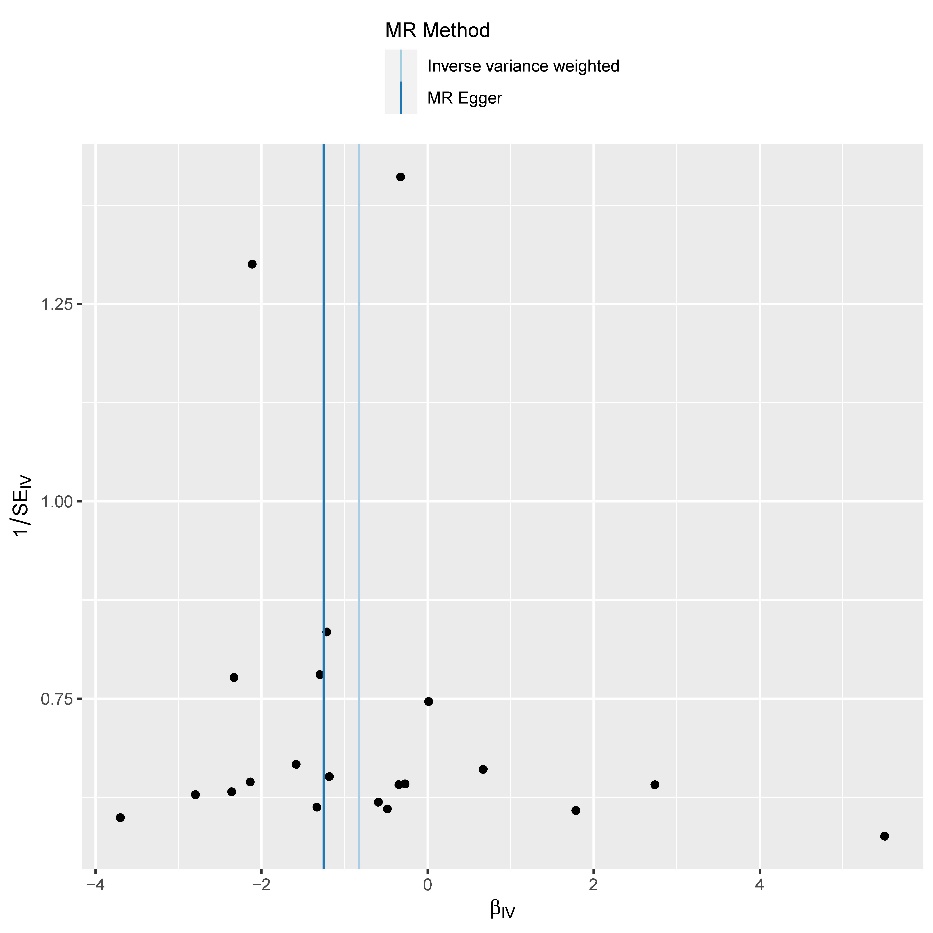


Funnel plot of genetic association estimates for gamma−glutamylglutamine on Alzheimer's disease


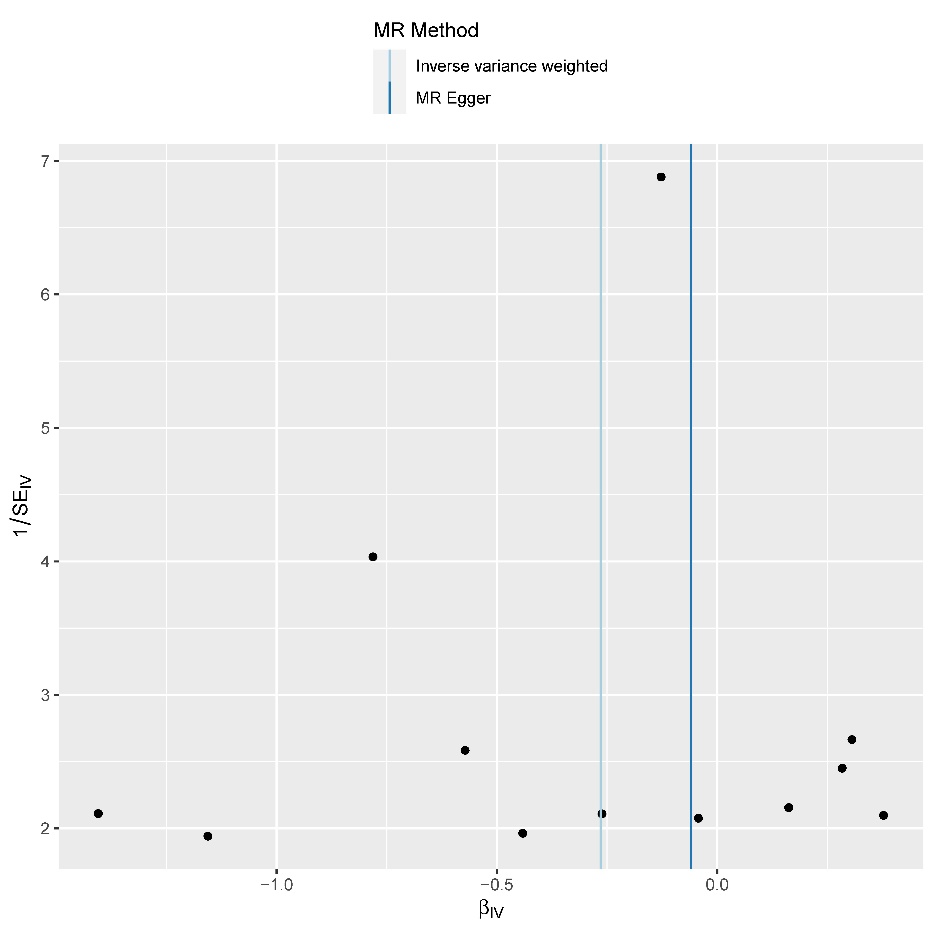


Funnel plot of genetic association estimates for leucylalanine on Alzheimer's disease


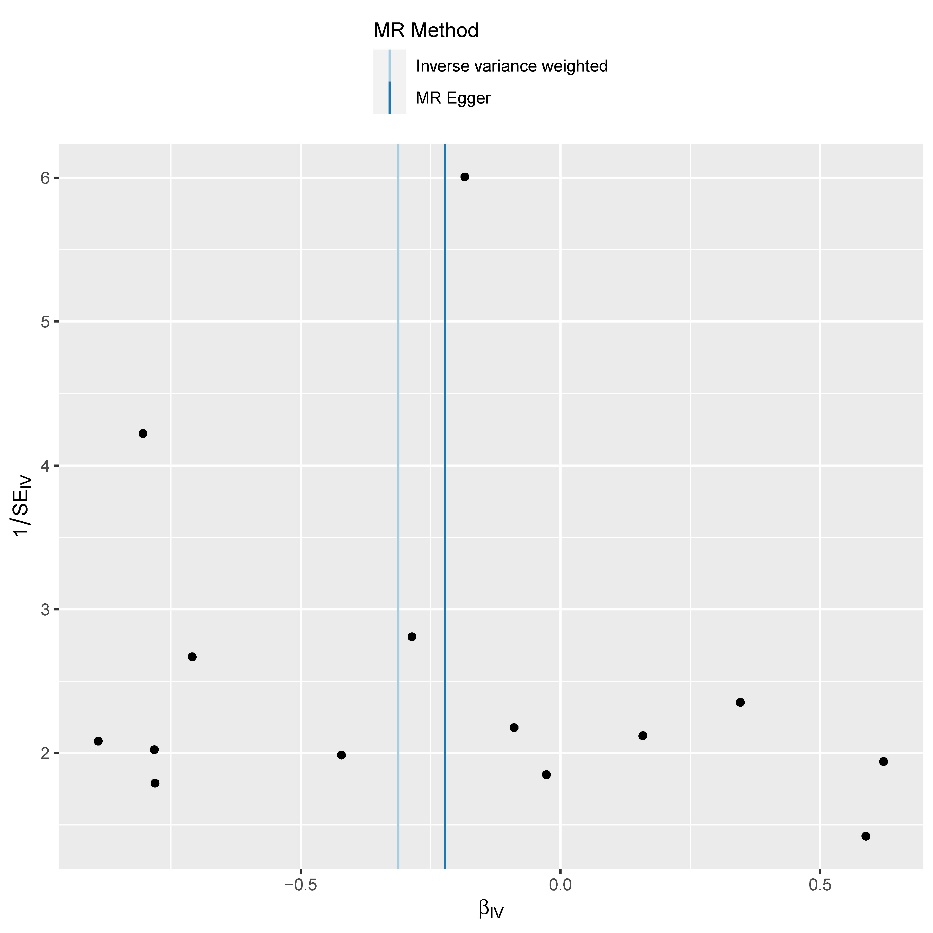


Funnel plot of genetic association estimates for phenylalanylserine on Alzheimer's disease


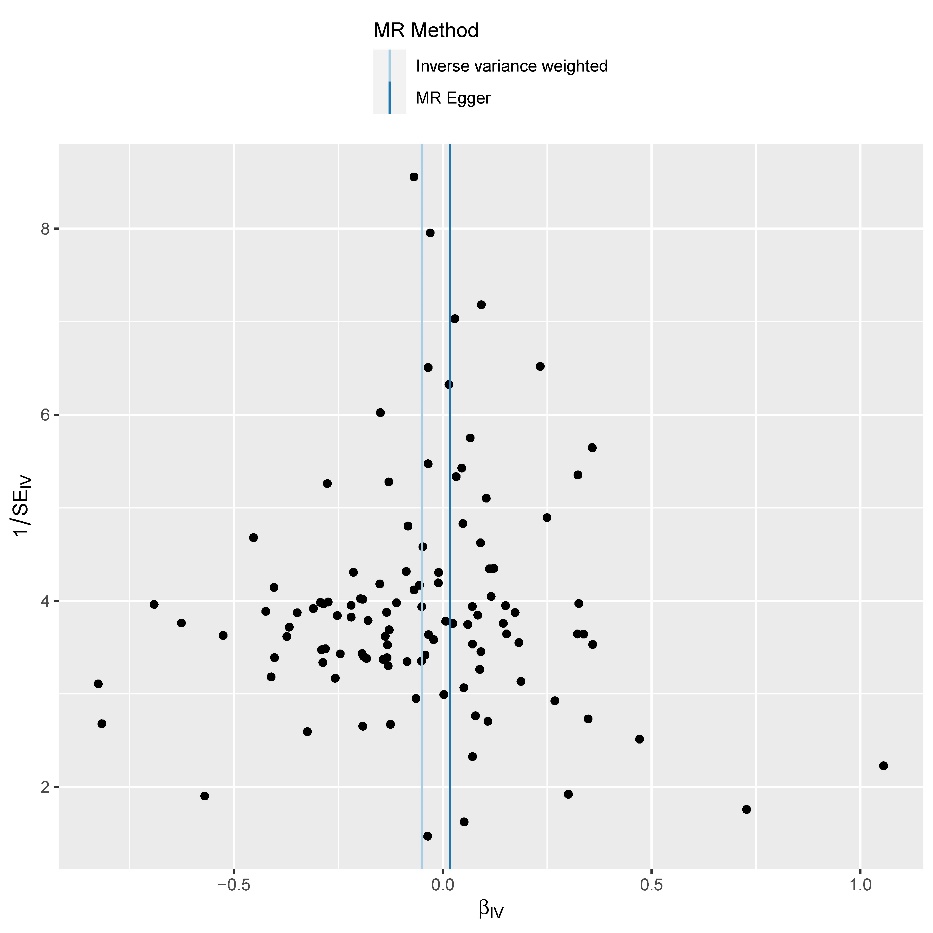


Funnel plot of genetic association estimates for ibuprofen on Alzheimer's disease


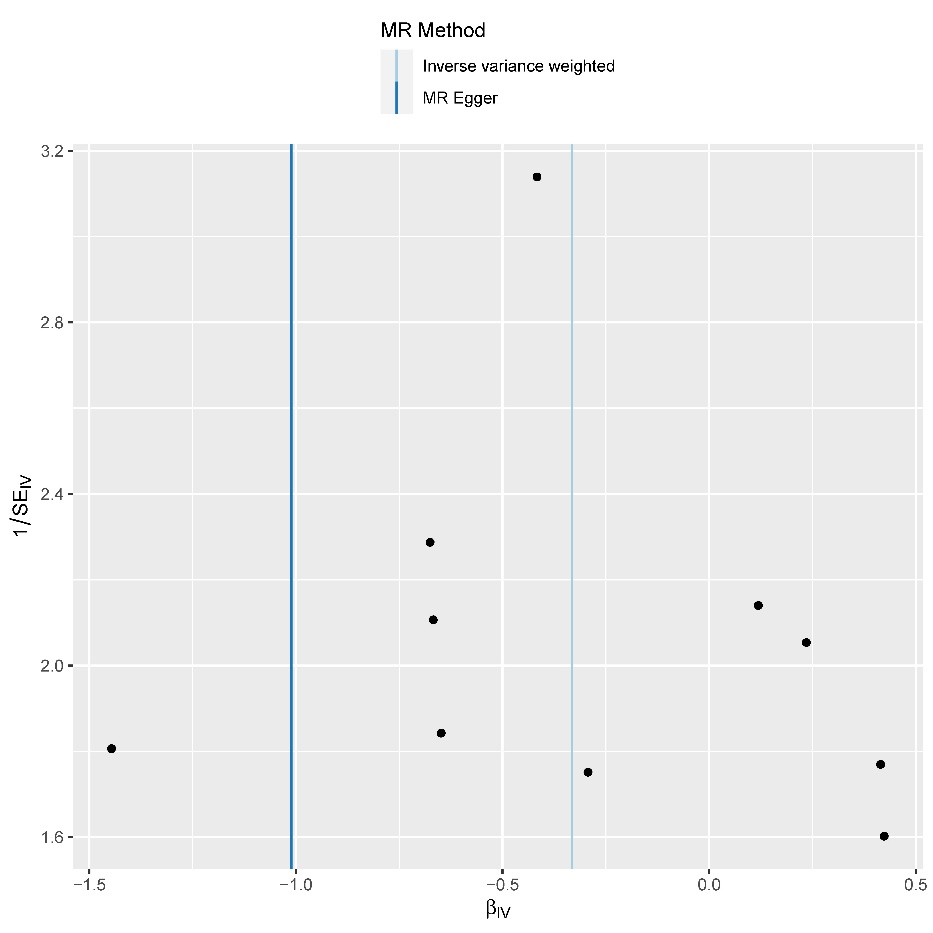


Funnel plot of genetic association estimates for X−09789 on Alzheimer's disease


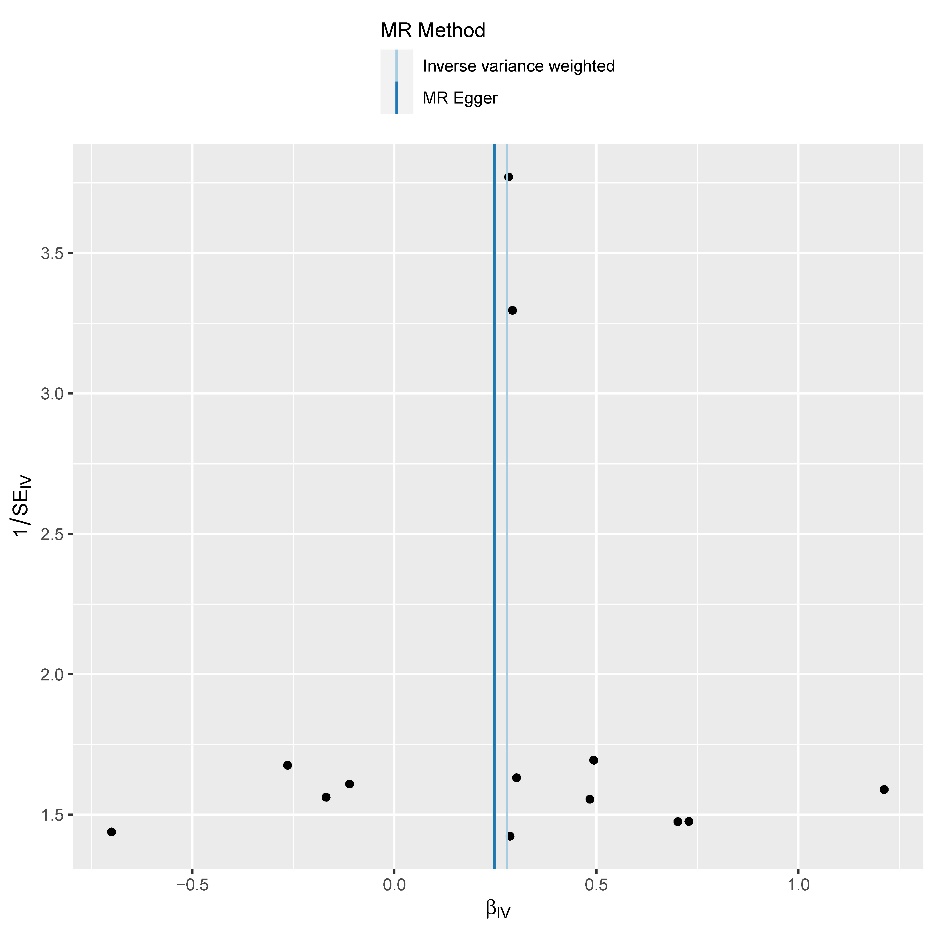


Funnel plot of genetic association estimates for X−11478 on Alzheimer's disease


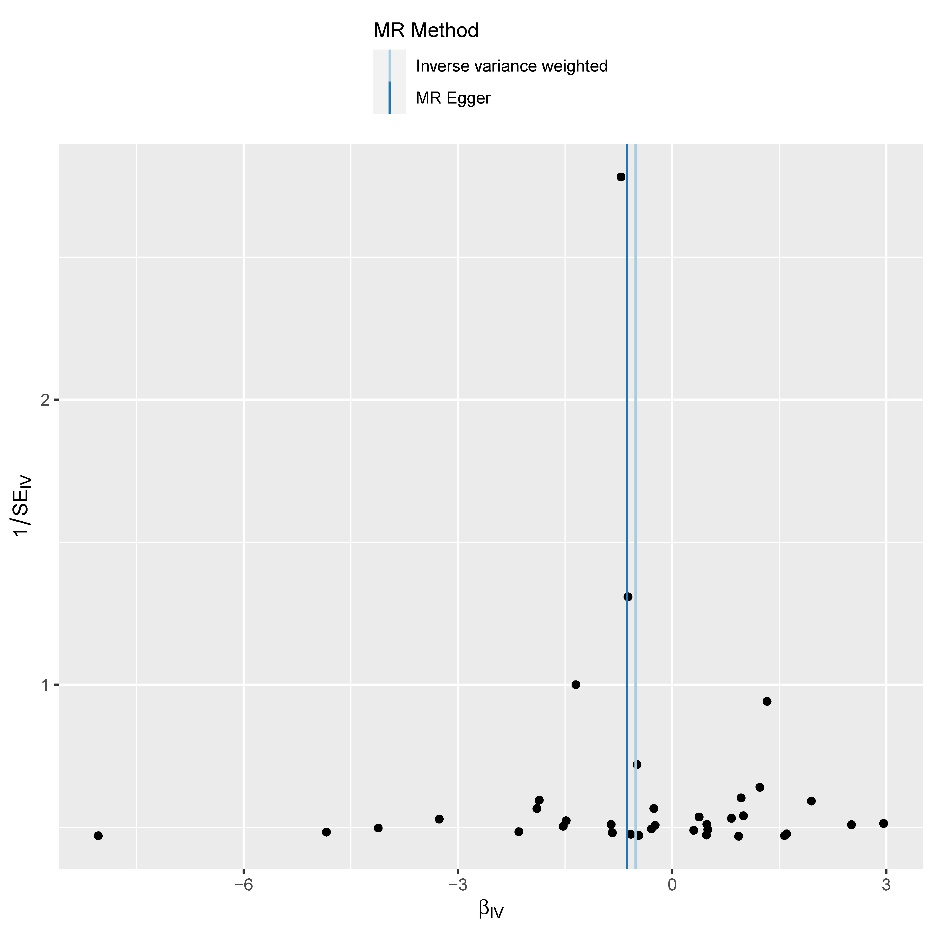


Funnel plot of genetic association estimates for X−12029 on Alzheimer's disease


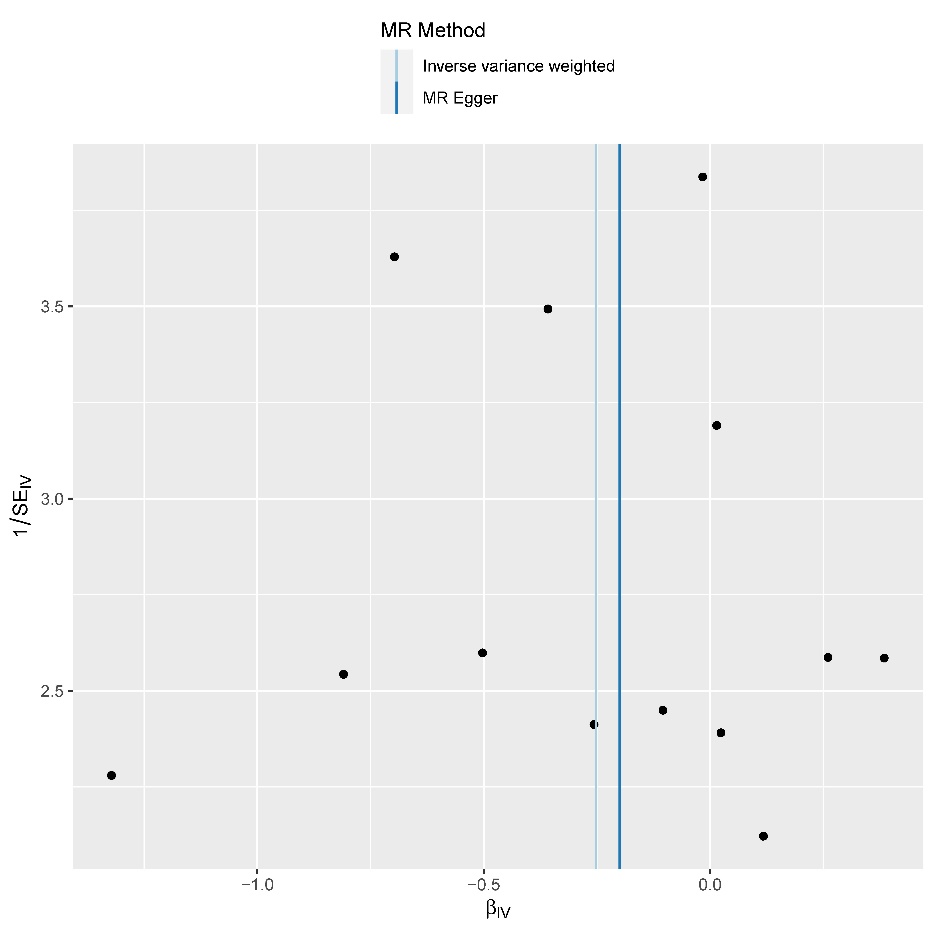


Funnel plot of genetic association estimates for X−12039 on Alzheimer's disease


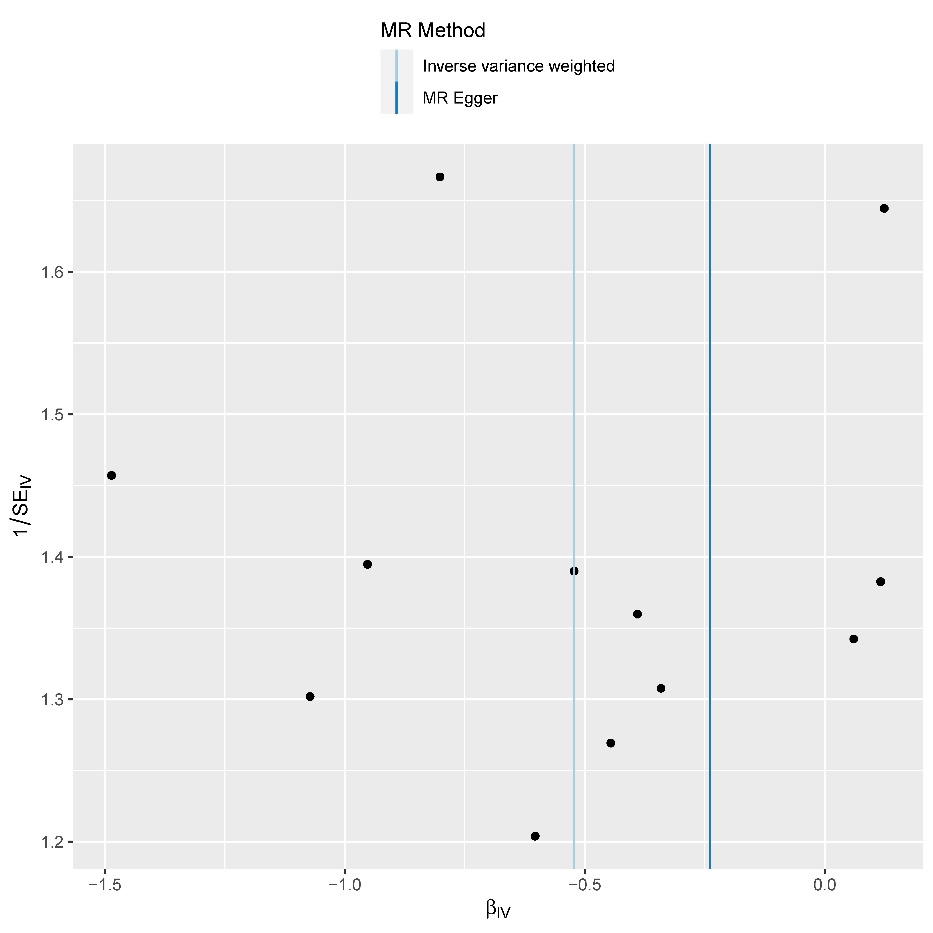


Funnel plot of genetic association estimates for X−12680 on Alzheimer's disease


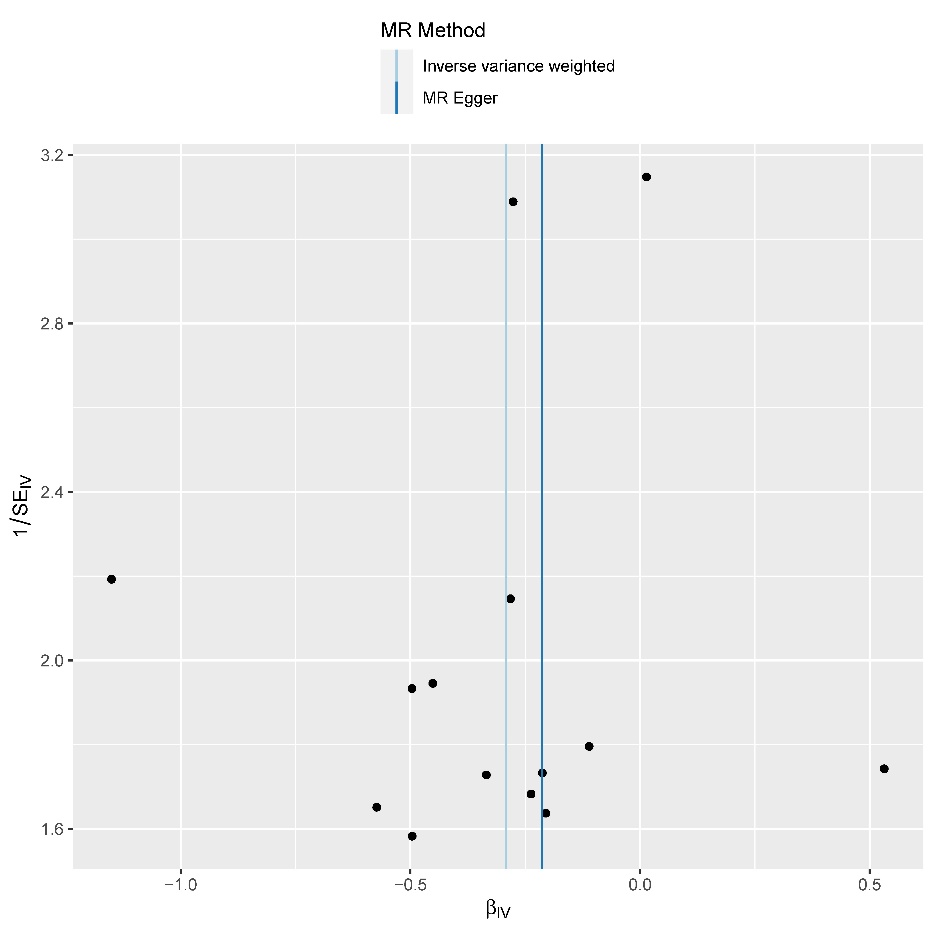


Funnel plot of genetic association estimates for X−12850 on Alzheimer's disease





**Supplementary Figure S3.** Forest plot for the genetic association derived from IVW of the metabolites identified in the replication and meta-analysis on the risk of Alzheimer's disease in EADB stage I. EADB, the European Alzheimer & Dementia Biobank; IVW, inverse variance weighted; OR, odds ratio; 95% CI, confidence interval; NSNPS, number of single nucleotide polymorphisms
